# Gpnmb defines a phagocytic state of microglia linked to cell death in prion disease mouse model

**DOI:** 10.1038/s41467-026-73003-5

**Published:** 2026-05-12

**Authors:** Davide Caredio, Giovanni Mariutti, Lisa Polzer, Beatrice Gatta, Martina Cerisoli, Yasmine Laimeche, Giulia Miracca, Jeanne Droux, Mohamad El Amki, Marc Emmenegger, Marian Hruska-Plochan, Susanne Wegener, Magdalini Polymenidou, Matthias Schmitz, Inga Zerr, Elena De Cecco, Adriano Aguzzi

**Affiliations:** 1 Institute of Neuropathology, University Hospital Zurich, University of Zurich, Zurich, Switzerland; 2 Department of Quantitative Biomedicine, University of Zurich, Zurich, Switzerland; 3 Department of Neurology, University Hospital Zurich, University of Zurich, Zurich, Switzerland; 4 Department of Neurology, National Reference Center for TSE, Georg-August University, Göttingen, Germany; 5 Institute for the Science of the Aging Brain (ISAB), St. Gallen, Switzerland; 6 Present Address: Medical Immunology, Department of Laboratory Medicine, University Hospital Basel, Basel, Switzerland

**Keywords:** Microglia, Cell death in the nervous system, Chronic inflammation, Prion diseases

## Abstract

Neurodegenerative disorders display brain region tropism accompanied by the emergence of distinct cellular states that contribute to disease pathogenesis, with molecular alterations occurring predominantly in glial cells. Here we show the emergence of a microglial state with distinct spatial distribution in the brains of terminally sick prion-infected mice characterized by high expression of Gpnmb (glycoprotein non-metastatic melanoma protein B), transcriptional signatures consistent with phagocytic activity, and increased expression of lysosomal genes in regions undergoing pronounced cell death. We find that this cellular state is not induced by pathological protein aggregates but by soluble factors released by dying cells regardless of the initiating insult. This work defines Gpnmb⁺ microglia as a distinct phagocytic state that links cell death to microglial activation and reveals a generalizable mechanism by which microglia respond to cell loss.

## Abstract (web summary)

Prion diseases cause neurodegeneration associated with microglial alteration. Here, the authors show in a prion disease mouse model that phagocytic Gpnmb⁺ microglial state reflects regional responses to cell death and lysosomal stress rather than direct sensing of amyloid fibrils.

## Introduction

Misfolding of the cellular prion protein (PrP^C^) into aggregation-prone infectious prions (PrP^Sc^) is the causative event of prion diseases (PrDs). These pathologies are heterogeneous but inevitably lethal due to progressive neuronal death, massive glia activation (gliosis) and brain spongiosis^1^. The observation that different cell types exhibit varying levels of susceptibility to infection with distinct prion strains suggests that additional factors may influence prion replication and contribute to toxicity. Some of these factors may be cell-autonomous to neurons. For example, in Creutzfeldt-Jakob disease (CJD) and Gerstmann-Sträussler-Scheinker disease, cortical and hippocampal parvalbumin-expressing interneurons show increased vulnerability and death^2,3^, whereas in fatal familial insomnia, pathological hallmarks mostly occur in the thalamus without any major involvement of these neurons^4^. However, the onset and the progression of motor dysfunctions correlate closely with molecular changes within glial cells and occur long before any neuronal loss is detectable^5–11^. Indeed, mounting evidence highlights non-cell autonomous mechanisms in PrDs progression, suggesting that glial perturbation, rather than neuronal loss, might be the primary driver of the pathology^5,6^.

Microglia are brain-resident immune cells which play a crucial role in the maintenance of central nervous system (CNS) homeostasis and in responses to injury and disease^12^. Reactive microglia are spatially associated with PrP^Sc^ in human and murine PrDs^13–16^. Prion-infected, microglia-depleted mouse brains exhibit increased neurotoxicity and accelerated disease progression, suggesting a role for microglia in prion clearance and containment^17–19^. Recent studies indicate that microglia and astrocytes can cooperate in a highly coordinated manner to maintain tissue homeostasis in PrD, particularly by jointly phagocytosing apoptotic neurons, with microglia actively modulating astrocyte activity^20,21^. However, microglia can also show impaired phagocytic capabilities due to overwhelming accumulation of PrP^Sc^ that cannot be effectively degraded^22^. In the latter case, microglia may even facilitate the spread of prions within the CNS by migrating to different brain regions and releasing extracellular vesicles containing PrP^Sc^^23^. Additionally, oligodendrocyte precursor cells (NG2 glia) play a neuroprotective role in prion diseases by regulating microglial activity. Their selective depletion exacerbates prion-induced neurodegeneration, driven by increased prostaglandin E2 production by microglia^10^. These mechanisms underscore the role of microglia not only in responding to prion infection but also in propagating it, highlighting their dual role in PrDs pathogenesis^24^.

Previous studies investigating transcriptional changes in prion diseases have typically analyzed either the entire mouse brain or specific brain regions, without considering prion tropism for specific brain regions and cell types^25,26^. The spatiotemporal transcriptomic analysis presented here provides a map of the molecular landscape across brain regions as prion disease progresses. Among the most prominent changes, we identify the emergence of a distinct microglial state characterized by expression of *Gpnmb*, arising at 30 weeks post-prion infection and preferentially occupying regions undergoing severe neurodegeneration. Gpnmb has been linked to disease-associated microglia (DAM) in Alzheimer’s disease^27^, multiple sclerosis^28^ and frontotemporal dementia^29^, where it is associated with lysosomal and phagocytic programs, although its precise functional contribution remains unclear. Consistently, *Gpnmb*⁺ microglia in our dataset showed increased expression of the lysosomal stress marker *Lgals3* and the lysosomal proton pump subunit *Atp6v0d2*, responsible for the acidification of the luminal environment. This transcriptional profile points to an enhanced role in phagocytosis and metabolic adaptation to the engulfment of cell debris. Importantly, the late appearance and regional confinement of *Gpnmb*⁺ microglia argue against a direct response to prion replication and instead point to induction by local tissue injury. Here, we demonstrate across multiple complementary models that cues from dying cells are sufficient to trigger the Gpnmb program in microglia, establishing this lysosomal and phagocytic microglial state as a causal response to cell death in degenerating brain regions.

## Results

### A unique transcriptional signature in prion-infected thalamus

Eleven-week-old C57BL/6 mice were injected intraperitoneally with prion-containing brain homogenate (RML6 strain, the 6th consecutive mouse-to-mouse passage of mouse-adapted Rocky Mountain Laboratory sheep scrapie prions). Non-infectious brain homogenate (NBH) was injected as a control. At 24 weeks post-inoculation (wpi), prion-infected mice began exhibiting symptoms, including weight loss, motor impairment and increased lethargy^5^. Brain samples were harvested at both 30 wpi and the terminal stage of the disease (32-33 wpi, the latest timepoint at which mice could be humanely euthanized). We performed the experiment twice for each condition (prion or NBH).

**Fig. 1:**
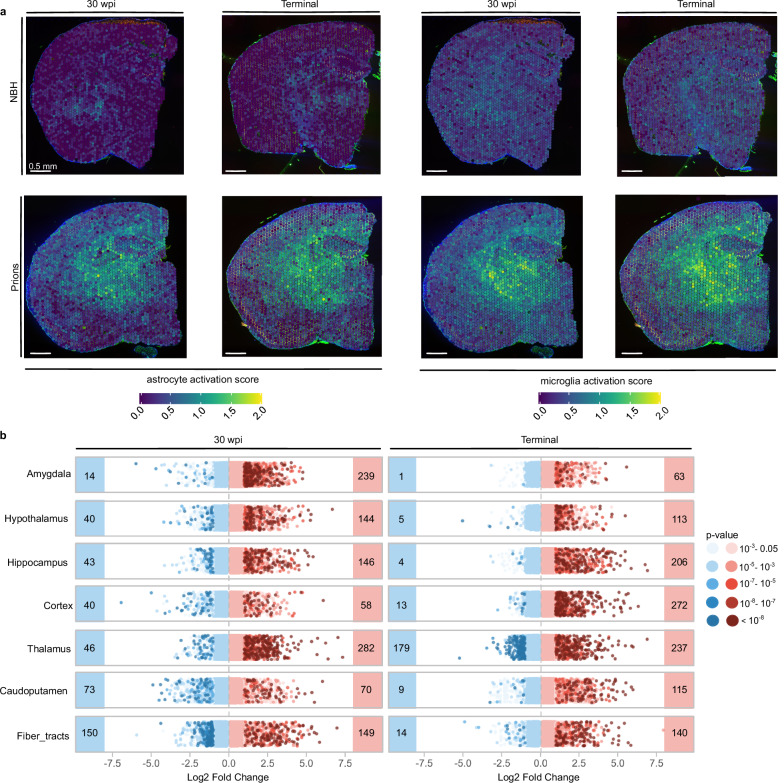
Progressive spatiotemporal glial activation and transcriptional remodeling in prion disease. **a** Spatial transcriptomic slices comparing prion-infected vs. uninfected mice at 30 wpi and terminal disease. Heatmaps: astrocyte (mean expression levels of *Gfap*, *Vim*, *Serpina3n*, *C4b*, *B2m*) and microglia (mean expression levels of *Apoe*, *C1qa*, *C1qb*, *C1qc*, *Cd68*) activation. Scale bars represent 0.5 mm. **b** Upregulated (red) and downregulated (blue) DEGs across brain regions at 30 wpi and terminal disease. Dots indicate genes with an absolute log2 fold change > 1, color-coded according to their adjusted *P*-values. Differential expression was assessed using DESeq2 (negative binomial distribution model and two-sided Wald tests), *p*-values were adjusted using the Benjamini–Hochberg method to control the false discovery rate (FDR). Genes with adjusted *p *< 0.05 were considered significant.

Spatial transcriptomics (ST) analysis revealed a conspicuous progressive intensification of astroglial and microglial activation during the symptomatic stage of PrD. For each ST map spot, we calculated an activation score as the mean expression levels of established markers of glial reactivity^30^: *Gfap*, *Vim*, *Serpina3n*, *C4b* and *B2m* for astrocytes and *Apoe*, *C1qa*, *C1qb*, *C1qc* and *Cd68* for microglia (Fig. 1a). To ensure reproducibility, ST brain slices from two mice per condition were analyzed for consistency of cellular responses (Fig. 1a and Supplementary Fig. 1a).

Next, we investigated how distinct brain regions respond transcriptionally to prion infection. We performed differentially expressed gene (DEG) analysis between prion-infected brain regions and NBH controls (Fig. 1b, and Supplementary Data 1 and 2). At 30 wpi, we observed progressive transcriptomic deviations across multiple brain regions, including the amygdala, hypothalamus, hippocampus, and thalamus, with upregulated genes predominating (27 % downregulated vs. 73 % upregulated). At the terminal stage, also the cortex started showing an increasing number of upregulated genes. At the terminal stage, the thalamus showed more downregulated than upregulated genes, whereas amygdala, hypothalamus and hippocampus maintained the same trend observed at 30 wpi.

For each filtered set of regional DEGs (|log2FC| > 0.5 and FDR < 0.05), shared modulated genes between timepoints were analyzed using over-representation analysis to identify enriched pathways (Supplementary Figs. 1b and 2, and Supplementary Data 3). Most dysregulated genes across all analyzed brain regions were associated with glial cells and were related to neuroinflammatory processes, gliosis, or astrocyte development. In the hypothalamus, we detected upregulation of genes controlling cytokine regulation and leukocyte activation. In stark contrast with the rest of the brain, the thalamus displayed a unique profile of enriched immune-related pathways, with a pronounced focus on tumor necrosis factor (TNF) production, cytokine regulation and antigen presentation. This peculiar transcriptional profile, together with the extent of transcriptional dysregulation observed during disease progression, suggests that the thalamus is the brain region most affected by infection with RML6 prions.

### Distinct Gpnmb^+^ cell-type identity characterizing the late PrD

**Fig. 2:**
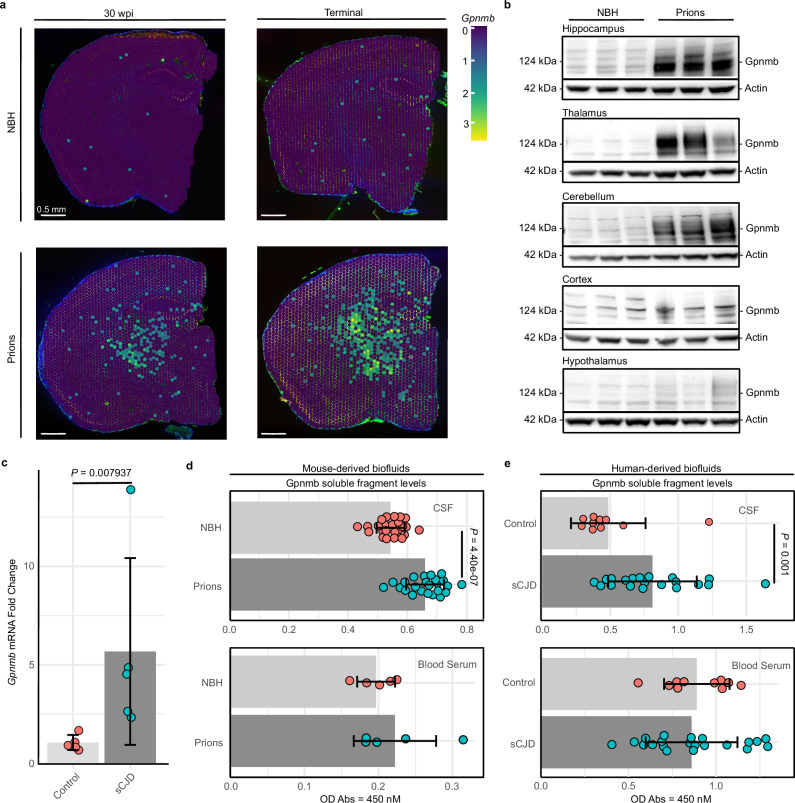
Spatiotemporal increase of Gpnmb expression during prion disease progression. **a** Progressive upregulation of Gpnmb mRNA in spatial transcriptomic slices of prion-infected vs. noninfected (NBH) mouse brains at 30 wpi and terminal disease. Color code: heatmap as indicated. Scale bars represent 0.5 mm. **b** Western blot analysis of terminally prion-sick mice displaying Gpnmb protein levels in hippocampus, thalamus, cerebellum, cortex, hypothalamus. **c** *GPNMB* qPCR on prefrontal cortex from sCJD and sex/age-matched control patients. Each dot represents one patient (*n* = 5). **d** Gpnmb soluble fragment levels in cerebrospinal fluid (CSF) and blood serum from prion-infected mice and NBH controls collected at the terminal stage. Each dot represents one mouse (CSF: prion *n* = 24, NBH *n* = 25; blood: prion and NBH *n* = 5). **e** Gpnmb soluble fragment levels in CSF and blood serum from sCJD patients and controls. Each dot represents one patient (CSF: sCJD *n* = 20, control *n* = 10; blood: sCJD *n* = 21, control *n* = 10). Data in (**c**–**e**) are presented as mean ± s.d., and statistical significance was assessed using a two-sided Wilcoxon rank-sum test.

To identify the cell populations responsible for the observed transcriptional alterations, we applied the STdeconvolve algorithm^31^, which infers gene expression profiles by analyzing the transcripts covariance and proportion of each profile at each ST pixel (Supplementary Fig. 3). The algorithm then associates these profiles with cell-specific markers, hence enabling the characterization of cell-type abundance and spatial distribution across the brain region during disease progression^31^. At 30 wpi, no common gene expression profile emerged in two independent experiments in the prion condition (Supplementary Fig. 4 and Supplementary Data 4). However, in terminally sick mice, we noted the appearance of a cell-type identity characterized by the expression of *Gpnmb* in prion-infected samples (Supplementary Fig. 5 and Supplementary Data 5). *Gpnmb* upregulation was already detectable at 30 wpi, particularly in the thalamus, despite not being among the most enriched gene of any cell identity (Fig. 2a and Supplementary Fig. 6a). Western blots on terminally sick mice confirmed that Gpnmb was upregulated in the thalamus, hippocampus and cerebellum of prion-infected mice, but not in the hypothalamus and cortex (Fig. 2b and Supplementary Fig. 6b). Regions with high Gpnmb levels also showed strong PrP^Sc^ deposits (Supplementary Fig. 6c).

### Gpnmb and its shed fragment are increased in CJD patients

We assessed *GPNMB* mRNA levels in the autopsied prefrontal cortex of five patients suffering from sporadic CJD (sCJD) and in five age and sex-matched controls (Supplementary Data 6), as this region is highly affected in sCJD^1,32^. qPCR analysis showed a significant upregulation of *GPNMB* in the prion condition (Fig. 2c), underscoring the similarity of this trait in humans and mice^33^.

GPNMB is known to undergo shedding by the metalloprotease ADAM10, with its soluble N-terminal domain being released in biofluids (CSF^34–37^, serum^38,39^, and urine^40^). Increased shed GPNMB levels in biofluids are considered a valid biomarker of biological aging^41^, but its usefulness as a biomarker for neurodegenerative diseases is still debated^42–44^. We observed a significant increase in shed Gpnmb levels in the CSF of prion-infected mice (control *n* = 25, prion *n* = 24; *p*-value = 4.4 × 10⁻⁷, Fig. 2d), as well as in the CSF from sCJD patients (control *n* = 10, sCJD *n* = 20; *p*-value = 1.3 × 10⁻^3^, Fig. 2e), while levels in the serum were unchanged in both mice (NBH *n* = 5, prion *n* = 5) and humans (control *n* = 10, sCJD *n* = 21) (Supplementary Data 7). These data emphasize a significant alignment in pathophysiological characteristics between human and murine PrDs.

### Gpnmb expression is increased in microglia during PrD progression

**Fig. 3:**
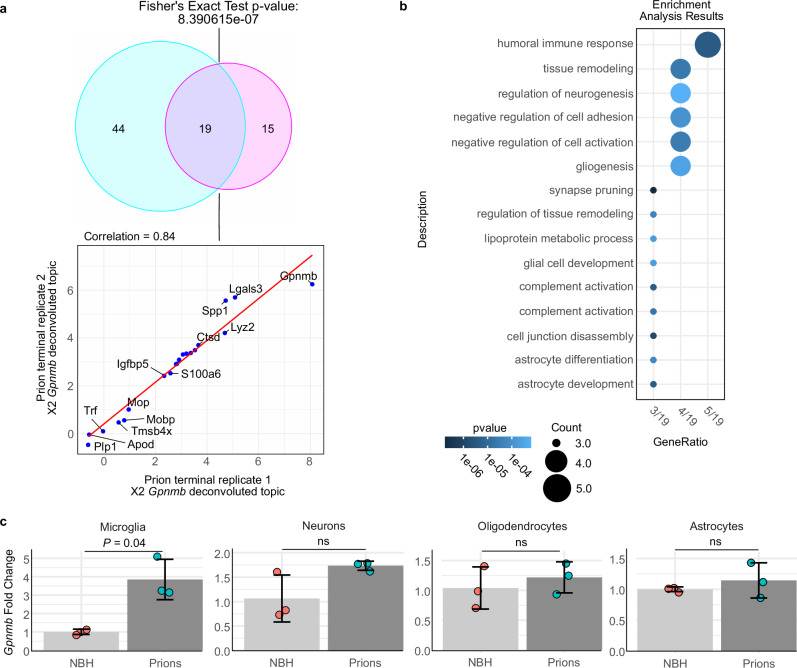
Gpnmb upregulation in prion disease linked to microglial transcriptional programs. **a** Venn diagram and correlation analysis of the ST deconvoluted Gpnmb^*^ cell types identified in prion terminal replicates. The Venn diagram shows a significant overlap of nineteen genes (Fisher’s Exact Test, *p* = 8.39 × 10⁻⁷), which are displayed in the scatter plot below; the plot indicates a strong correlation (R = 0.84) between the gene expression profiles of these cell types across both replicates. **b** Over-representation analysis (ORA) results of the nineteen overlapping genes, highlighting biological processes. Significance was assessed with Fisher’s Exact Test, and *p*-values were adjusted using the Benjamini–Hochberg method to control FDR (**c**) qPCR showing relative mRNA abundance of *Gpnmb* in sorted CNS cell types (microglia, neurons, oligodendrocytes and astrocytes) between NBH and prion-infected mice. Each dot represents one mouse (*n* = 3). Data are presented as mean ± s.d., and statistical significance was assessed using two-sided unpaired Welch’s *t* tests.

To better understand the source of Gpnmb upregulation, we analyzed the genes co-segregating with *Gpnmb* in the molecular profiles listed by STdeconvolve (Supplementary Fig. 5). The two prion-infected samples shared 19 genes belonging to the *Gpnmb*^+^ molecular profile (Fig. 3a); most of them were associated with synaptic pruning and immune response, suggesting microglial involvement (Fig. 3b and Supplementary Data 8). STRING pathway analysis of these genes recognized three clusters, two of which were associated with oligodendrocytes (*Mbp*, *Mobp*, *Plp1*) and microglia (*Apod*, *Apoe*, *Trf*, *Serpina3n*, *C1qc*, *C1qb*, *C1qa*, *Lyz2*, *Tyrobp*, *Ctsd*), while the third (*Gpnmb*, *Spp1*, *Lgals3*, *Vim*, *Igfbp*, *S100a6*, *Tmsb4x*) was unassigned (Supplementary Fig. 7a). Therefore, we isolated and FACS sorted nuclei from major CNS cell types (microglia, neurons, oligodendrocytes and astrocytes) from prion-infected and NBH control mouse brain samples (*n* = 3; Supplementary Fig. 7b, c) and assessed Gpnmb expression levels. qPCR analysis revealed that prion-induced *Gpnmb* upregulation is driven primarily by microglia (Fig. 3c).

### Gpnmb^+^ microglia transcriptionally resemble phagocytic microglia

**Fig. 4:**
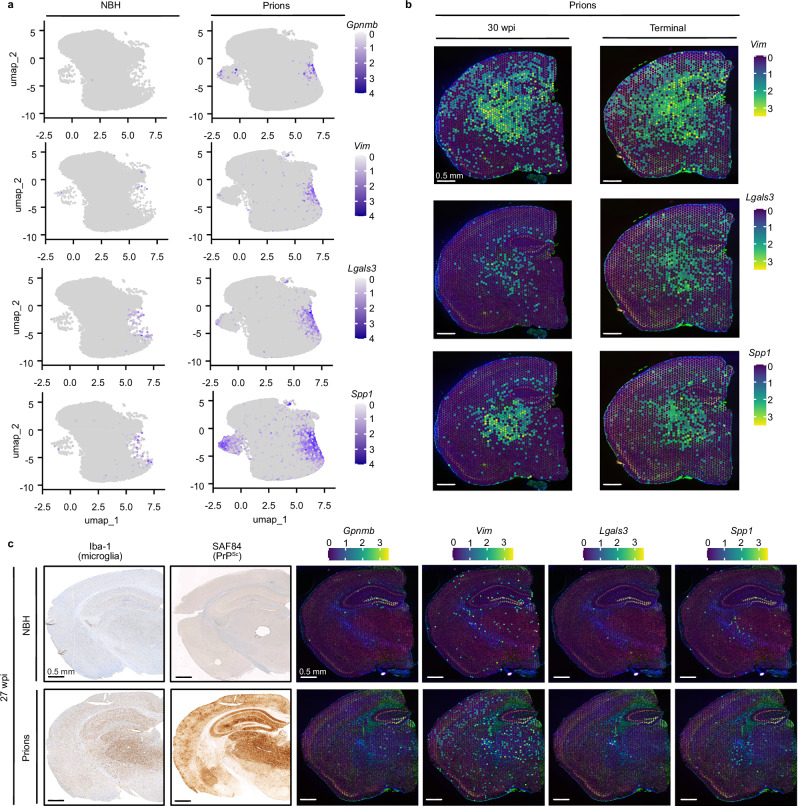
Single-cell and spatial association of Gpnmb with disease-associated microglial markers in prion disease. **a** UMAP plots from a published dataset (Slota et al., 2022) displaying gene expression of *Gpnmb*, *Vim*, *Lgals3* and *Spp1* in control (NBH) and prion-infected conditions. Each plot shows the distribution of these genes within different cell populations, with higher expression indicated by darker shades. **b** Spatial distribution of *Vim*, *Lgals3* and *Spp1* in prion-infected brain sections at 30 wpi and terminal disease. Heatmap: gene expression levels. Scale bars represent 0.5 mm. **c** Immunohistochemical (IHC) staining of brain sections at 27 wpi shows the microglial marker Iba-1 and PrP^Sc^ (SAF84), with spatial transcriptomics data illustrating the expression of *Gpnmb*, *Vim*, *Lgals3* and Spp1. Scale bars represent 0.5 mm.

Microglia exist in distinct transcriptional states associated with various degrees of activation and phagocytic capacity. To better characterize the identified Gpnmb^+^ microglial population at the transcriptional level, we relied on a dataset of single-cell RNA sequencing of terminally ill prion-infected mice and non-infected controls^25^. Unbiased subclustering analysis of the dataset^25^ (Supplementary Fig. 8a–c) revealed that *Gpnmb* was predominantly expressed by cells belonging to the phagocytic microglia subpopulation (cluster 6) and, to a lesser extent, to MHC type-II microglia (cluster 10) only in the prion-inoculated condition (Supplementary Fig. 8d, e). Among the 19 shared genes belonging to the ST *Gpnmb*^+^ profiles identified in the two prion-infected samples, only *Spp1*, *Lgals3* and *Vim* were specifically upregulated in clusters 6 and 10, whereas the others were also expressed by other microglial subclusters (Fig. 4a and Supplementary Fig. 8d, e). We then analyzed the spatial expression of *Spp1*, *Lgals3* and *Vim* in our ST dataset. While *Vim* showed a broader and less specific spatial expression already at 30 wpi, *Lgals3* and *Spp1* exhibited noticeable spatial co-localization with *Gpnmb* in the thalamus and, to a lesser extent, in the hippocampus at both 30 wpi and the terminal stage in the prion-infected condition (Fig. 4b), as confirmed also by Western Blot of prion-infected and control mouse brains (Supplementary Fig. 9a, b). We hypothesized that *Gpnmb*^+^ microglia is induced by the extreme neuroinflammation characteristic of late-stage prion infections. We then conducted a targeted spatial transcriptomics experiment at 27 wpi, a stage when prion-infected mice exhibit symptomatic manifestations, including weight loss (Supplementary Fig. 9c) and motor impairments, alongside ongoing microgliosis^5^. Immunohistochemistry showed reactive microglia and prion deposits, particularly in the thalamus, but among the four genes, only *Vim* was upregulated at this stage (Fig. 4c). This suggests that the appearance of *Gpnmb*^+^ microglial subpopulation is not a consequence of increased microgliosis.

We conclude that the identified *Gpnmb*^+^ microglial subpopulation emerging during late PrD progression shares key similarities with phagocytic microglial subpopulations and is transcriptionally defined by the co-upregulation of *Lgals3* and *Spp1*. Its appearance at later stages of disease may represent a response to drastic changes in the surrounding environment and/or to unidentified secreted stimuli.

### Cell loss rather than prion deposition induces Gpnmb^+^ microglia

**Fig. 5:**
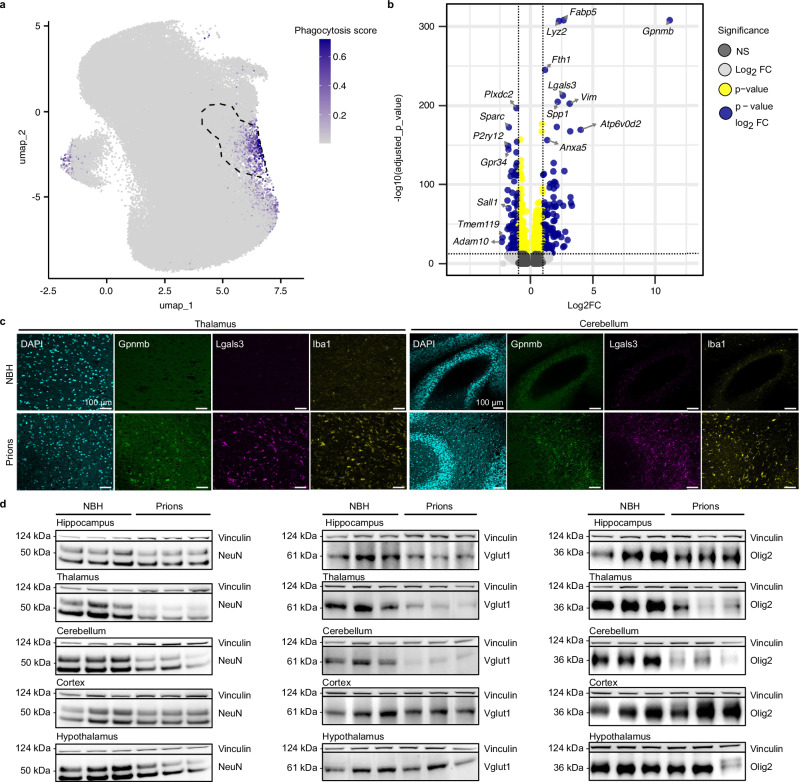
Concordant single-cell and tissue-level association of Gpnmb with phagocytic microglia. **a** UMAP plot illustrating the distribution of the Phagocytosis score in microglial cells from single-cell RNA sequencing data, with clusters showing high phagocytic activity. Purple color coding represents higher phagocytic score expression. The dotted line represents the overall density of cluster 6. **b** Volcano plot showing the differential gene expression analysis between *Gpnmb*⁺/Phagocytic Cluster 6⁺ and *Gpnmb*⁻/Phagocytic Cluster 6⁺ microglial cells, derived from scRNA-seq data (Slota et al. 2022). Differential expression was assessed using DESeq2 (negative binomial distribution model and two-sided Wald tests), *p*-values were adjusted using the Benjamini–Hochberg method to control the false discovery rate (FDR). Genes with adjusted *p *< 0.05 were considered significant. **c** Brain sections of terminally ill prion-infected mice stained with DAPI (cyan), Gpnmb (green), Lgals3 (magenta) and Iba1 (yellow). Scale bars represent 100 μm. **d** Western blot showing the levels of NeuN, a neuronal marker, Vglut1, an excitatory neuronal marker, and Olig2, mature oligodendrocytes marker, across different brain regions in controls (NBH) and prion-infected (Prions) samples at terminal disease.

Neuronal loss is a hallmark of late PrD that becomes widespread after the onset of cognitive and behavioral deficits^45^. To test if sustained neuronal death and/or prion deposition is a trigger of *Gpnmb*^+^ microglia, we analyzed a published RNA sequencing dataset of microglial cells treated with apoptotic neurons^46^. We extracted the DEGs from the phagocytic and non-phagocytic population^46^, and compared it with the previously analyzed single-cell dataset on prion-infected mouse brains^25^. We expressed their similarity as a Phagocytic Score. More specifically, for each microglial cell, we extracted the counts of genes associated with phagocytic and non-phagocytic conditions. The mean expression levels for phagocytic (*µ*_*phagocitic*)_ and non-phagocytic *(µ*_*non-phagocitic*_) genes were then calculated. The Phagocytosis score’ was determined by the ratio: *µ*_*phagocitic*_ /*µ*_*non-phagocitic*_. This ratio was visually represented as a color-coded scale to indicate the relative phagocytic activity of the microglial cells. This analysis revealed that microglia that specifically respond to neuronal loss formed a distinct subset (highlighted in purple) within broader phagocytic Cluster 6 (outlined in black) (Fig. 5a). Hence, microglial phagocytic responses vary depending on the nature of the engulfed material (apoptotic debris vs. prion aggregates). The subset of phagocytic microglia reacting to apoptotic stimuli overlapped with those upregulating *Gpnmb*, supporting that *Gpnmb* upregulation in microglia is driven by apoptotic neurons.

We then examined gene expression in *Gpnmb*⁺ vs *Gpnmb*⁻ microglia within phagocytic Cluster 6 (Supplementary Data 9). Known phagocytosis markers were upregulated (Fig. 5b), including the receptor of phosphatidylserine *Anxa5* (annexin V), an “eat me” signal present on apoptotic bodies. *Fth1* (ferritin), which is associated with ferroptosis, a form of programmed cell death induced by iron overload during extensive phagocytosis^47^, was also upregulated in *Gpnmb*^+^ microglia. The most upregulated gene in the *Gpnmb*^+^ phagocytic cluster was *Atp6v0d2*, a vesicular ATPase essential for lysosomal acidification and enzymatic activity, along with other lysosomal/phagosomal genes (Supplementary Fig. 10a and Supplementary Data 9). Building on the observation that *Gpnmb*⁺ microglia in the phagocytic cluster exhibit enhanced lysosomal activity, we used Lgals3 as a marker of lysosomal dysfunction^48–50^, to assess lysosomal stress in *Gpnmb*⁺ microglia. Immunofluorescence staining of terminal stage prion-infected mice revealed a robust spatial co-upregulation of Gpnmb and Lgals3 in thalamus, cerebellum and hippocampus (mostly close to the alveus and corpus callosum), but not in cortex or hypothalamus (Fig. 5c and Supplementary Fig. 10b, c). Although correlative, these observations indicate that exposure to apoptotic stimuli triggers the emergence of a *Gpnmb*^+^ microglial subpopulation with enhanced phagocytic activity, closely resembling that seen in prion infection. However, prion infection also leads to the presence of additional subpopulations with lower phagocytic capacity and reduced *Gpnmb* expression.

We then quantified neuronal loss in specific brain regions using the same samples shown in Fig. 2b. The neuronal marker NeuN (Fig. 5d left panel, and Supplementary Fig. 11d) was conspicuously reduced in the thalamus, cerebellum, and hippocampus, where Gpnmb, Lgals3, and Spp1 expression levels were highest (Supplementary Fig. 9a, b). In contrast, NeuN levels in the cortex and hypothalamus did not show significant changes. Consistent with recent findings^45^, our data indicated that the degenerating neurons were primarily excitatory (Vglut1^+^) neurons (Fig. 5d middle panel, and Supplementary Fig. 11d), while inhibitory (Vgat^+^) neurons were largely unaffected (Supplementary Fig. 11b, d). The most affected brain regions also exhibited marked synaptic degeneration (Supplementary Fig. 11a, d), reflected by a strong reduction in the presynaptic marker Syp, likely driven by the loss of Vglut1^+^ excitatory neurons. Furthermore, we observed significant loss of oligodendrocytes, assessed by reduced Olig2 signal, in the cerebellum and thalamus, accompanied by extensive microgliosis in the latter (Fig. 5d right panel, and Supplementary Fig. 11c, d). Finally, GFAP immunoreactivity broadly increased throughout the brain (Supplementary Fig. 11c, d).

To identify the primary driver of Gpnmb upregulation, we treated murine BV2 cells (microglia/macrophage-like cells) with prion-infected brain homogenates, lipopolysaccharide (LPS, an inducer of general neuroinflammation), or conditioned medium from apoptotic cells. Treatment with prion-infected or NBH control brain homogenates did not modify *Gpnmb* levels (Supplementary Fig. 12a). Since the total prion content in brain homogenates is stoichiometrically low, we enriched PrP^Sc^ from infected brains by NaPTA precipitation^51^ (Supplementary Fig. 12, b). Non-infectious brain homogenates served as controls. However, even after exposure to increasing concentrations of NaPTA-purified prions (0.25 %, 0.5 %, and 1 % w/v of the initial brain homogenate), Gpnmb upregulation was not observed (Supplementary Fig. 12, c). BV2 cells treated with conditioned medium from stably prion-infected but non-degenerating GT1-7 cells also failed to show any increase in Gpnmb expression (Supplementary Fig. 12, d). Hence, neither prion deposits nor soluble factors released during prion replication are responsible for the upregulation of Gpnmb.

To determine whether microglial Gpnmb upregulation is part of a broader inflammatory response, we treated BV2 cells with LPS, a well-known inducer of microglial activation and pro-inflammatory cytokine release. LPS exposure induced only Spp1 upregulation (Supplementary Fig. 13a, b), suggesting that Spp1 plays a broader role in neuroinflammation.

**Fig. 6:**
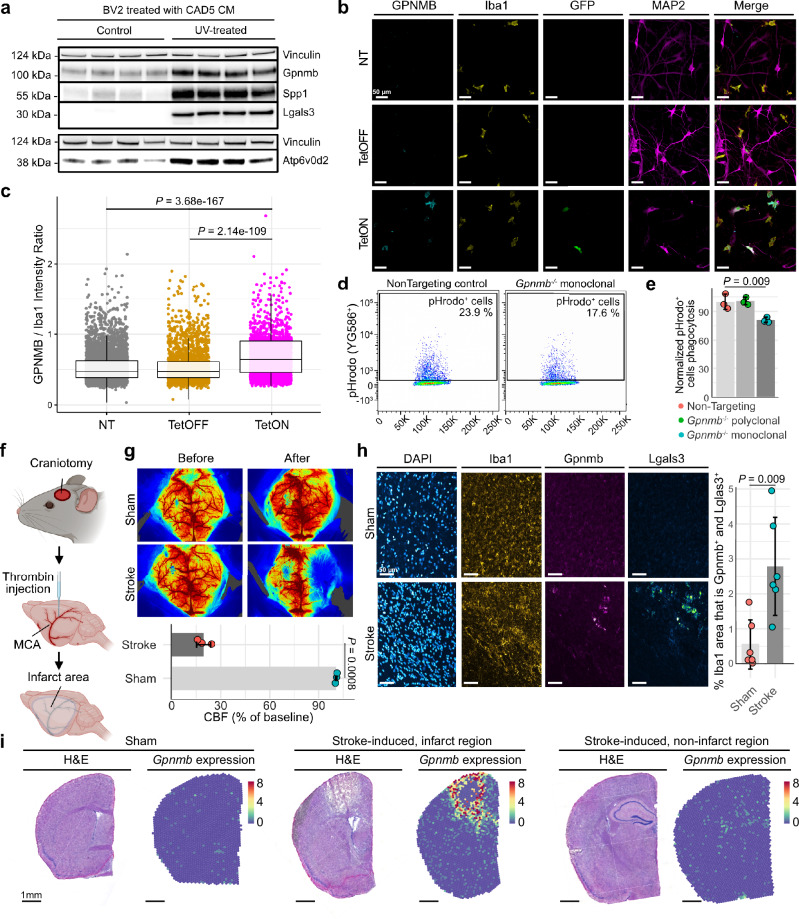
Gpnmb expression associated with apoptotic sensing and ischemic injury in microglia. **a** Western blot of BV2 cells treated with conditioned medium (CM) from UV-treated CAD5 cells (apoptotic bodies) or healthy CAD5 cells (control), showing Gpnmb, Spp1, Lgals3 and Atp6v0d2 protein levels relative to Vinculin. **b** Immunofluorescence of hiPSC-derived neuron–microglia co-cultures showing Gpnmb (cyan), Iba1 (yellow, microglia marker), EGFP (green) and MAP2 (magenta, neuronal marker) under non-treated (NT), TetOFF and TetON conditions. Doxycycline induces EGFP expression and neuronal death. Scale bars, 50 μm. **c** Quantification of Gpnmb fluorescence from panel b) normalized to Iba1 across conditions. Each data point represents a single microglial cell from two biological replicates (*n* = 2). Box plots show median, interquartile range (25th–75th percentiles), and whiskers extending to the most extreme values within 1.5 x the interquartile range. Pairwise comparisons were performed using two-sided Wilcoxon rank-sum tests with Benjamini-Hochberg correction. **d** Flow cytometry analysis of phagocytic activity in control (non-targeting, NT) and Gpnmb-depleted BV2 cells, showing percentage of positive cells following pHrodo-labeled apoptotic CAD5 cells. **e** Quantification of phagocytic activity (pHrodo^+^ cells) in non-targeting control, Gpnmb-depleted polyclonal and monoclonal BV2 cells. Each dot represents an assayed aliquot of that cell preparation in three independent experiments. **f** Schematic of the middle cerebral artery occlusion (MCAO) stroke model (Created in BioRender. De Cecco, E. (2026) https://BioRender.com/5vig2sa). **g** Cerebral blood flow (CBF) measurements before and after MCAO, displayed as heatmaps and quantified relative to baseline. Each dot represents one mouse (*n* = 3). **h** Immunofluorescence of sham and stroke brain sections showing DAPI (blue), Iba1 (yellow), Gpnmb (magenta) and Lgals3 (green) expression, with related triple-pixel colocalization quantification. Each dot represents one brain slice (two slices per mouse, three mice per condition; *n* = 3). Scale bars, 50 μm. **i** Representative H&E-stained sections and spatial *Gpnmb* expression maps from sham and stroke mice (strokemap.cn). Scale bars, 1 mm. Data in (**e**,** g**, and **h**) are presented as mean ± s.d. Statistical significance in (**e**) was assessed using two-sided one-way ANOVA followed by Dunnett’s multiple comparisons test; in (**g**, **h**) was using two-sided Welch’s* t* tests.

To model an apoptotic environment, conditioned medium from UV-irradiated murine catecholaminergic CAD5 cells was added to BV2 cells (Supplementary Fig 13c). This resulted in a pronounced increase in Gpnmb, Spp1, Lgals3, and Atp6v0d2 expression (Fig. 6a and Supplementary Fig. 13d), while Vim levels remained unchanged (Supplementary Fig. 13e). Similar results were observed when BV2 cells were treated with conditioned medium from UV-irradiated BV2 cells (Supplementary Fig. 13f), showing that the identity of the dead cells is not critical. Notably, exposure of BV2 cells to different fractions of the apoptotic CAD5 conditioned medium (Supplementary Fig. 14a) showed that robust upregulation of Gpnmb in BV2 cells was driven by soluble factors released during apoptosis, rather than by direct phagocytosis of apoptotic bodies (Supplementary Fig. 14a–c).

We then utilized a human co-culture model combining iNets (induced neuronal networks)^52^ with human microglia^53^. iNets, derived from iCoMoNSCs^54^, self-organize into synaptically connected, electrophysiologically active neuronal networks that mature into long-lived functional systems, including mixed neuronal and glial populations but lacking microglia. We then induced neuronal death by overexpressing toxic levels of EGFP^55,56^ via a doxycycline (TetON)-responsive lentiviral vector. To distinguish the effects of transgene activation, cultures were analyzed either in the presence of doxycycline (TetON), in its absence (TetOFF), or without viral transduction (NT). This enabled us to investigate the microglial response to neuronal death in a controlled environment that recapitulates human biological complexity. After 12 days of EGFP activation, we observed significant (p = 1.49e-23) neuronal loss (Supplementary Fig. 15a, b). In response, microglia exhibited a morphological shift from a ramified to an amoeboid shape (Supplementary Fig. 15c), indicative of activation, and displayed a robust upregulation of Gpnmb (Fig. 6b, c).

### Aβ fibrils do not induce GPNMB in absence of cell death

Because Gpnmb⁺ microglia have been reported in proximity to amyloid plaques in Alzheimer’s disease^27^, we investigated whether exposure to Aβ aggregates alone is sufficient to induce Gpnmb expression in the absence of overt cell death.

BV2 cells were treated with increasing concentrations of Aβ pre-formed fibrils (PFFs; Supplementary Fig. 16a) for 48 h. To exclude potential confounding effects arising from Aβ PFFs-induced cell death (Supplementary Fig. 13f), cell viability was monitored under all conditions. Gpnmb expression remained unchanged across all Aβ PFFs concentrations (Supplementary Fig. 16b, c) when cell viability remained unaffected (Supplementary Fig. 16d). For control, BV2 cells exposed to conditioned medium from UV-irradiated CAD5 cells showed significant Gpnmb upregulation (Supplementary Fig. 16e, f) despite no change in cell viability (Supplementary Fig. 16g).

To validate these observations in a higher-order system, human iPSC-derived neuronal–microglial co-cultures were treated with Aβ PFFs or LPS. Again, no increase in GPNMB expression was observed in any condition (Supplementary Fig. 17a, b) when neuronal number remained unchanged (Supplementary Fig. 17c).

To further corroborate our findings, we analyzed single-cell RNA sequencing data from microglia sorted from the AD mouse model 5XFAD and wild-type controls across 1, 3, 6, and 8-9 months of age^57^. In this model, Aβ aggregation starts at 1.5 months, with plaques appearing after 2-3 months and symptoms emerging after 6 months, while overt cell death occurs only at terminal disease stages (9 months)^58,59^. Canonical DAM microglia markers (*Apoe*, *Clec7a*, *Cst7*, and *Trem2*) were upregulated at early stages of disease (1-3 months of age) and increased with disease progression (Supplementary Fig. 18a). In contrast, *Gpnmb* was expressed only in a small subset of microglial cells at the terminal stage of disease (9 months), when massive cell death is ongoing. Upregulation of the other transcripts associated to *Gpnmb*^+^ profile (*Lgals3*, *Spp1*, and *Vim*) was also confined to small subsets of microglial cells, but increased at earlier timepoints (Supplementary Fig. 18b).

Together, these data indicate that, in the absence of cell death, neither Aβ fibrils nor LPS induce Gpnmb upregulation in microglia.

### Gpnmb depletion impairs phagocytosis of apoptotic cells

We then directly assessed the role of Gpnmb in microglial phagocytosis. Apoptotic cells from UV-irradiated CAD5 cells were labeled with pHrodo-PE and added to wild-type or Gpnmb-deficient BV2 cells (Supplementary Fig. 19a). Complete depletion of Gpnmb in BV2 cells significantly reduced the percentage of pHrodo^+^ cells (*p* < 0.05) (Fig. 6d, e and Supplementary Fig. 19b, d), whereas partial depletion (Supplementary Fig. 19a) did not. Hence, Gpnmb enhances microglial phagocytosis, with even low expression levels being sufficient to sustain its function.

### Cell death drives Gpnmb upregulation in vivo

To move beyond the spatiotemporal correlation between Gpnmb⁺ microglia and severe neurodegeneration observed in prion disease, we directly tested whether cell death – in absence of pathological protein aggregates – is sufficient to drive the emergence of this microglial state in vivo. We employed a focal cortical stroke model in which ischemia was induced by stereotactic injection of thrombin into a cerebral artery to generate a clot in situ (Fig. 6f). Sustained reduction in cerebral blood flow was confirmed by laser speckle imaging (Fig. 6g and Supplementary Fig. 20). Immunofluorescence imaging showed increased expression and co-localization of Gpnmb and Lgals3 in microglia located at the center of the infarcted region of stroke mice compared with the corresponding region of sham controls (*n* = 3; Fig. 6h).

To confirm our results, we explored a publicly available spatial transcriptomic dataset of photo-induced stroke mice and sham controls^60^. In this model, cell death was massive, and *Gpnmb* as well as *Lgals3* showed strong upregulation all along the penumbra region surrounding the necrotic core of the infarcted region (Fig. 6i), thus supporting our previous findings.

Together, our results indicate cell death as a direct driver of the Gpnmb⁺Lgals3⁺ microglial state beyond protein misfolding disorders and establish a generalizable microglial response mechanism to cell death-inducing insults.

## Discussion

Microglial activation is a hallmark of prion disease, yet the molecular diversity of reactive microglial states remains poorly defined. Our spatial transcriptomic analysis of prion-infected mouse brains revealed that transcriptional changes were dominated by glial cells, whereas neuronal signals were less apparent, likely masked by extensive gliosis. Among brain regions, the thalamus exhibited the most pronounced microglial changes, with gene ontology analysis highlighting enrichment of TNF-related pathways, previously linked to other neurodegenerative diseases^61–64^.

Deconvolution of spatial data uncovered the emergence of a discrete *Gpnmb*^+^ microglial state at the terminal stage of the disease, predominantly localized within the most severely affected areas. Gpnmb and its shed extracellular fragment were increased in the cerebrospinal fluid from both prion-infected mice and sCJD patients. These results indicate that this microglia program is a conserved and clinically relevant feature of prion pathology. *Gpnmb*^+^ microglia displayed increased expression of DAM-associated genes such as *Spp1*, *Lgals3* and *Vim*^46,57,65^, accompanied by repression of homeostatic markers including *P2ry12*, *Sall1* and *Tmem119*. Functional annotation highlighted pathways involved in apoptotic cell recognition, lipid metabolism, and lysosomal function were strongly represented, in agreement with observations from other pathological contexts^27,66,67^. Among the most strongly upregulated transcripts we identified *Atp6v0d2*, a core component of the V-ATPase machinery, *Anxa5*, the phosphatidylserine receptor, and the ferroptosis-associated genes *Fabp5* and *Fth1*. V-ATPase has been implicated in neurodegenerative diseases^68–74^ where it enables microglia to digest apoptotic neurons^75^. Given that *Atp6v0d2* is required for phagolysosomal maturation^76^, the induction of the Gpnmb program in microglia may promote its lysosomal degradative capacity. This hypothesis aligns with evidence that Gpnmb deletion disrupts V-ATPase assembly in endothelial cells^77^.

Notably, *Gpnmb*-associated transcriptional profile changes emerged only at late stages of the disease, when prion deposition and gliosis were already widespread, pointing to secondary consequences of tissue injury rather than direct responses to prion replication. Since massive neuronal loss is a feature of advanced stage prion disease^45^, we hypothesized that apoptotic burden may represent the primary trigger. Indeed, regions enriched for Gpnmb⁺ microglia corresponded to areas with the greatest neuronal and oligodendroglial loss. Neuronal vulnerability appeared to be largely restricted to excitatory neurons, as reflected by the loss of Vglut1, while inhibitory neurons were comparatively preserved, consistent with recent reports^45^. The concomitant decrease in presynaptic markers in the same areas likely reflects the depletion of excitatory inputs. Together, these data indicate that the local apoptotic burden is a primary determinant of Gpnmb^+^ microglial induction.

A similar association between Gpnmb expression and microglial clearance of cell debris has been described in developmental white matter remodeling^78,79^, demyelinating disease^28,79,80^, rheumatoid arthritis^28,81^, ischemic injury^82^ and in *Grn*^*-/-*^ mouse model of frontotemporal lobar degeneration (FTLD)^83^. We therefore directly tested whether apoptotic cues are sufficient to induce Gpnmb. In BV2 cells, Gpnmb and its other associated markers were robustly upregulated upon exposure to conditioned medium from apoptotic UV-irradiated catecholaminergic cells, whereas treatment with prion-infected brain homogenates, purified prions, or conditioned medium from non-degenerating prion-propagating cell failed to induce Gpnmb expression. The effect persisted after removal of apoptotic bodies from the conditioned medium, indicating that soluble factors released by dying cells are sufficient to trigger a shift towards a highly phagocytic *Gpnmb*^+^ state. The same response occurred in our human iPSC-derived model, where neuronal death elicited strong microglial GPNMB induction accompanied by a morphological transition from ramified to amoeboid cells. These results demonstrate that Gpnmb program and its mechanism of induction are conserved across species and holds under near-physiological conditions.

Reports from Alzheimer’s disease suggested that Gpnmb⁺ microglia localize around Aβ plaques^27^ and that Aβ monomers may trigger Gpnmb upregulation in microglia in vivo^84^. However, other studies indicate that Gpnmb induction occurs primarily in response to neuronal or myelin debris and not to Aβ aggregates^85,86^. Using two independent in vitro models, we found that microglia failed to upregulate Gpnmb in response to Aβ fibrils when cell viability was preserved. In agreement, reanalysis of microglia from 5XFAD mice showed early activation of canonical DAM programs during plaque development, whereas Gpnmb expression appeared restricted to late stages marked by severe neurodegeneration. Importantly, careful control of toxicity proved essential, as microglia respond not only to apoptotic neurons but also to other dying neighboring cells, including oligodendrocytes and even microglia themselves. Consequently, apparent aggregate-driven Gpnmb induction may therefore reflect secondary cell death.

Functionally, impaired phagocytosis in Gpnmb-deficient microglia indicates a direct contribution of this protein to debris clearance and emphasizes its relevance for diseases characterized by progressive tissue destruction.

Finally, to causally demonstrate that pathological protein aggregates are not required for the emergence of Gpnmb⁺ microglia we employed two independent models of acute ischemic injury. In both cases, Gpnmb⁺Lgals3⁺ microglia accumulated in areas of extensive injury, paralleling our in vitro observations and prion disease spatial transcriptomic data. Importantly, they indicate that the association between Gpnmb⁺ microglia and amyloid or prion deposits reported in neurodegenerative settings likely reflects the presence of ongoing degeneration rather than a direct response to aggregated proteins and support the idea that cell death itself is sufficient to trigger the Gpnmb program.

While we demonstrate that Gpnmb promotes microglial clearance functions, the molecular mechanisms underlying this effect, the identity of the apoptotic cues inducing its expression, and the functional role of the shed ectodomain remain unresolved. In addition, current spatial transcriptomic approach is limited by spot-level resolution, which, even with deconvolution, cannot fully resolve single-cell states in densely populated regions. This is particularly relevant for markers such as *Serpina3n* and *C4b*, which are also associated with disease-associated oligodendrocyte states and may complicate cell-type attribution. Future studies integrating higher-resolution spatial methods with single-cell transcriptomics and orthogonal functional validation will be required to precisely define cell-type–specific roles of Gpnmb in neurodegenerative tissue remodeling. Furthermore, the sex of mice was not recorded and considered as a biological variable, which may represent an additional limitation of this study.

Collectively, our findings establish *Gpnmb*⁺ microglia as a distinct phagocytic state in prion disease that emerges when clearance demand becomes extreme and provide causal evidence linking tissue degeneration to its induction in vivo. The increase of *GPNMB* transcripts in the prefrontal cortex and soluble protein in the CSF of sCJD patients suggests potential translational relevance and raises the possibility that GPNMB may act as a marker of neurodegeneration.

## Methods

### Study approvals

Animal experiments were approved by the Veterinary Office of the Canton Zurich (animal permits ZH243/2018, ZH064/2022, ZH030/2023) and carried out in compliance with the Swiss Animal Protection Law. In Switzerland, permit numbers are equivalent to approval numbers.

For the experiment in Fig. 2c, prefrontal cortex cDNA from sCJD patients and age-matched controls was kindly provided by Prof. Legname's laboratory. These samples were previously used in one of his published studies^87^ under approved ethical permits. In brief, the control frontal cortex samples, obtained from individuals who died of a non-neurological condition, were generously provided by the MRC Edinburgh Brain Bank. RNA extraction was performed at the International School for Advanced Studies (Trieste, Italy), following a previously established protocol^88^. Ethical approval for the use of these control samples was granted by the East of Scotland Research Ethics Service REC 1 (reference number 16/ES/0084) and informed consent for the research use of post-mortem tissue was obtained from the relatives. For the sCJD frontal cortex samples, the study was approved by the institutional review board of the Carlo Besta Neurological Institute and conducted in compliance with ethics committee guidelines. Written informed consent was obtained in accordance with the Declaration of Helsinki (1964–2008) and the Additional Protocol on the Convention of Human Rights and Biomedicine concerning Biomedical Research (2005).

The study on sCJD and related control patients (Fig. 2e) conforms to the Code of Ethics of the World Medical Association. Informed consent was obtained from all prion disease patients or their legal next of kin, and the study was approved by the local ethics committee University Medical Center Göttingen, Von Siebold-Str. 3, 37075 Göttingen (No. 24/8/12, No. 11/11/93). Control samples were obtained through other observational studies that were also approved by the same ethics committee at the University Medical Center Göttingen (No. 19/11/09; No. 9/6/08). All samples were blinded to the investigators with respect to personal identifiers.

### Mice

#### Mouse husbandry

Mice were kept in a conventional hygienic grade facility, constantly monitored by a sentinel program aimed at screening the presence of all bacterial, parasitic and viral pathogens listed in the Federation of European Laboratory Animal Associations (FELASA). The light/dark cycle consisted of 12/12 h with artificial light (40 Lux in the cage) from 07:00 AM to 07:00 PM. The temperature in the room was 21 ± 1 °C, with a relative humidity of 50 ± 5 %. The air pressure was controlled at 50 Pa, with 15 complete changes of filtered air per hour (HEPA H 14 filter; Vokes-Air, Uster, Switzerland). Up to five mice were housed in IVC type II long cages with autoclaved dust-free Lignocel SELECT Premium Hygiene Einstreu (140–160 g/cage) (J. Rettenmaier and Söhne GmbH), autoclaved 20 × 21 cm paper tissues (Zellstoff), autoclaved hay and a carton refuge mouse hut as nesting material. Individual housing was avoided, and all efforts were made to prevent or minimize animal discomfort and suffering. Prion-inoculated and control-injected mice were regularly monitored for the development of clinical signs and humane termination criteria were employed.

#### Mouse prion model

For Visium Spatial Transcriptomics, eleven-week-old C57BL/6J mice were intraperitoneally injected with 100 μl of RML6 prions (passage 6 of Rocky Mountain Laboratory strain mouse-adapted scrapie prions) containing 8.02 log LD_50_ of infectious units per ml. Control inoculations were performed using 100 μl of non-infectious brain homogenate (NBH) from CD-1 mice at the same dilution. Intraperitoneal injection was preferred to intracerebral injection because it allows for a slower progression of the disease and makes it possible to detect consistent neuronal loss at later stages. Mice sex was not recorded. After inoculation, mice were initially monitored three times per week. After clinical onset, mice were monitored daily. Mice were sacrificed at pre-defined time points: 27 weeks post inoculation (wpi), 30 wpi and terminal stage (corresponding to 31–34 wpi). Prion-inoculated mice allocated to the terminal group were sacrificed upon clear signs of terminal PrD, including piloerection, hind limb clasping, kyphosis and ataxia. Control-injected mice assigned to the latest time point group were sacrificed at the same time as terminally ill mice.

Mice were sacrificed by 4% isoflurane-induced deep anesthesia followed by decapitation, and brains were then removed from the skull and frozen in tissue embedding medium (OCT) on dry ice.

#### Mouse thrombin stroke model

We employed a previously established stroke model^89,90^. Mice sex was not recorded. Briefly, anaesthetized (mixture of midazolam 5 mg/kg, medetomidine 0.5 mg/kg, and fentanyl 0.05 mg/kg) BALB/c (*n* = 3 stroke and *n* = 2 sham) and C57BL/6J (*n* = 1 sham) mice were head-fixed in a stereotaxic frame, the fur on the head and neck was shaved, and a single midline incision was made between the eyes and ears to expose the skull. The left temporal muscle was gently retracted, and a small craniotomy was created over the M2 branch of the middle cerebral artery (MCA). After carefully removing the dura mater, a hand-pulled glass micropipette was inserted into the MCA. Prior to surgery, the pipette had been loaded with human alpha-thrombin (1.5 IU; HCT-0020, Hematologic Technologies Inc., USA). Alpha-thrombin was slowly injected into the vessel lumen to induce in situ clot formation. Once a stable clot had formed, the pipette was withdrawn, and cerebral ischemia was evaluated using laser speckle imaging (LSI). A reduction in blood flow of at least 50% from baseline within the MCA territory was defined as a successful stroke. Animals were euthanized at the experimental endpoint by transcardial perfusion under deep anesthesia (overdose of pentobarbital 200 mg/kg), in accordance with the approved animal protocols.

### Prion spatial transcriptomic (Visium 10X Genomics)

#### Spatial transcriptomic brain slicepreparation

For RNA quality control, brain tissue from 27 wpi, 30 wpi and terminal mice undergoing ST was processed into sectioning blocks as described above and stored at − 80 °C. OCT-embedded blocks were cryosectioned in a cryostat at − 20 °C to generate 10 μm slices fitting within the fiducial frame of Visium Spatial slides, following the 10x Genomics “Tissue Preparation Guide” (protocol CG000240, rev E). A tissue optimization experiment consisting of fluorescence footprint imaging was performed following the 10x Genomics “Tissue Optimization Guide” (protocol CG000238, rev E) and imaged on Eclipse Ti2-E microscope (Nikon) according to the 10x Genomics “Visium Spatial Gene Expression Imaging Guidelines” (protocol CG000241, rev E). Image analysis was performed using Fiji (ImageJ).

RNA quality of frozen tissue was measured with 4150 TapeStation system (Agilent Technologies) according to the 10x Genomics “Tissue Preparation Guide” (protocol CG000240, rev E). Only samples with RIN > 7.0 were considered for further processing. Visium spatial transcriptomics libraries were prepared according to the 10x Genomics “Visium Spatial Gene Expression Reagent Kits” (protocol CG000239, rev G). Libraries were then quantified using 4150 TapeStation system and sequenced on a NovaSeq 6000 (Illumina).

#### FASTQ File Handling

Raw sequencing data were organized using a custom shell script. This script created symbolic links for the FASTQ files based on metadata, facilitating subsequent processing. The metadata file was parsed to link the original FASTQ files stored in a designated directory to a new working directory. FASTQ files were generated and processed using the 10x Genomics SpaceRanger (v2.0.0) software. The SpaceRanger count function was used to align the reads to the reference genome (GRCm38) and generate spatial gene expression matrices. Default parameters were used for all steps.

#### ST Data pre-processing

We used the Seurat (v4.0.5) R package for downstream analysis of spatial transcriptomic data. The following steps were performed: the output files from SpaceRanger, including the spatial and HDF5 files, were loaded into Seurat using custom R functions. Variable features were identified using the FindVariableFeatures function with the selection method set to "vst" and nfeatures set to 2000. The data were normalized using the NormalizeData function and scaled using the ScaleData function.

#### ST Data integration and dimension reduction

To integrate multiple Seurat objects and perform dimensionality reduction, we first parsed and merged the Seurat objects from previously processed data. Variable features were identified for each sample using the variance stabilizing transformation (vst) method in Seurat, selecting 2000 features for integration. The data were then normalized and scaled to prepare for dimensionality reduction. Principal Component Analysis (PCA) was conducted on the variable features, and the top 30 principal components were used for further analysis. Uniform Manifold Approximation and Projection (UMAP) was then performed using the first 30 principal components to visualize the spatial transcriptomic data in a reduced dimensional space. The integration framework in Seurat was employed to identify clusters and remove batch effects from the data. Clustering was performed using the FindNeighbors and FindClusters functions. The results were visualized using DimPlot and SpatialPlot to generate UMAP plots and spatial maps, respectively. This approach allowed for detailed examination and interpretation of the spatial transcriptomic landscape, providing insights into the underlying biological processes.

#### Astrocytic and Microglial Activation Score Visualization

To assess gliosis levels (Fig. 1a and Supplementary Fig. 1a), we selected a set of genes related to astrocytes (*Gfap*, *Vim*, *Serpina3n*, *C4b*, *B2m*) and microglia (*Apoe*, *C1qa*, *C1qb*, *C1qc*, *Cd68*). The expression data for these genes were extracted from each Seurat object and gliosis scores were calculated as the mean expression level of the selected genes for each cell. These scores were added to the metadata of each Seurat object. Using the svglite package, spatial feature plots were generated to visualize the gliosis scores.

#### DEGs Analysis of different brain regions

For the differential expression analysis, we used the MAST package (v1.12.0) to identify DEGs between different conditions and brain regions. We defined a function to perform DEG analysis for each Seurat object, comparing conditions (prion-derived and NBH control) within each brain region. We cleaned the DEG results by removing genes with patterns such as "*mt*-" and "*Bc1*" to focus on relevant genes.

#### ST Spot deconvolution analysis

ST spot deconvolution was performed by using STdeconvolve^31^ enabling reference-free cell-type deconvolution of multi-cellular pixel-resolution ST data. Code availability here: https://github.com/JEFworks-Lab/STdeconvolve.git.

### Stroke spatial transcriptomic

Spatial transcriptomic data from photothrombotic stroke mice and sham controls were obtained from Han et al.^60^. Visualization of *Gpnmb* expression was produced using the online platform strokemap.cn that accompanied the published dataset.

### Immunohistochemistry of Brain Slices

Mouse brains were fixed in formalin followed by treatment with concentrated formic acid to inactivate prions. Brain sections (5 μm) were generated and deparaffinized using graded alcohols followed by antigen retrieval using 10 mM citrate buffer (pH 6.0).

Antibodies used and their working dilutions were as follows: anti- Prion protein monoclonal SAF84 antibody 1:200 (SPI Bio, A03208), anti- Iba1 1:2500 (Wako, 019-19741), and anti- GFAP 1:1000 (Dako, Z0334). Brain sections were counterstained with haematoxylin and eosin. The images were acquired using NanoZoomer scanner (Hamamatsu Photonics) and visualized using NanoZoomer digital pathology software.

### Immunofluorescence of Brain Slices

40 μm brain slices were washed with PBS three times and permeabilized with 0.2% Tween20 PBS (PBS-T; Sigma-Aldrich) for 30 min. Again, three washes with PBS were performed, after which the slices were incubated in a blocking solution with 4 % donkey serum (Jackson Immuno Research, NC9624464) for 40 min and then in a PBS-T solution containing various primary antibodies (see below) overnight at 4 °C. The samples were washed in PBS three times before and after staining with secondary antibodies in PBS-T for 1.5 h. Finally, they were stained with Hoechst (Thermo Fisher Scientific, 62249) and mounted onto glass slides for confocal microscopy using mounting medium (Dako, S3023). All incubation steps were performed with rocking.

Primary antibodies used and their working dilutions were as follows: anti- Gpnmb 1:200 (Goat, Biotechne, AF2330), anti- Lgals3 1:300 (Rat, Cedarlane, CL8942AP), and anti- Iba1 1:500 (Rabbit, Wako, 019-19741). Secondary antibodies used were: anti- goat IgG -AF488 1:1000 (Thermo Fisher Scientific, A11055), anti- rat IgG -AF647 1:1000 (Thermo Fisher Scientific, A21247), and anti- rabbit IgG -AF555 1:1000 (Thermo Fisher Scientific, A31572).

The samples were imaged using a laser scanning inverse confocal microscope (Leica Stellaris5, with Power HyD S detectors) equipped with a supercontinuum white light laser, which was used to generate excitation light at different wavelengths. The images in each channel were acquired using 1024 pixels in x and y and a line accumulation of 8. The stained slices were imaged using a 10-fold magnification lens (HC PL APO CS2, NA = 0.4, air) to show characteristics typical of each imaged brain region, as well as with a 20-fold magnification lens (HC PL APO CS2, NA = 0.4, air) for higher resolution images. For the colocalization analysis of immunofluorescence, TIFF images containing immunofluorescence data were processed to quantify the overlap between specific protein markers (Gpnmb, Lgals3 and Iba1) across various brain regions. Each image was read, and individual channels were extracted for DAPI, Gpnmb, Lgals3 and Iba1.

For prion-induced brain slices, positive cells expressing these markers were counted by applying a threshold to each channel and a logical mask was used to identify colocalized cells. These results were combined with metadata, enabling statistical analysis. Independent *t* tests were performed to compare the prion and NBH conditions across different brain regions and boxplots were created to visualize the distribution of positive cell counts between these conditions.

For stroke-related slices, maximum-intensity projections of immunofluorescence images were analyzed in R using the EBImage package. Microglia were segmented based on Iba1 immunoreactivity using global Otsu thresholding followed by morphological filtering to remove noise and small objects. Gpnmb and Lgals3 signals were processed by morphological background subtraction and binarized using adaptive local thresholding with fixed parameters across all images. Resulting binary masks were refined by morphological operations and restricted to Iba1-positive regions. Colocalization was quantified at the pixel level as the percentage of Iba1-positive area that was double-positive for Gpnmb and Lgals3. Six independent images per condition were analyzed.

### Single nuclei isolation and FACS sorting

Brains were extracted from RML6 infected terminal-stage mice and immediately flash frozen. Half-brains were used for the isolation of nuclei. Each hemisphere was incubated in 7 ml of lysis buffer (11 ml Nuclei PURE Lysis Buffer (Nuclei Isolation Kit, Sigma-Aldrich, NUC201), 110 μl 10 % Triton X-100 (Nuclei Isolation Kit, Sigma-Aldrich, NUC201), 11 μl 1 M DTT (Sigma-Aldrich), 55 μl 40 U/µl RNAsin Plus (RNAsinPlus RNase Inhibitor, Promega, N2615)) for 10 min on ice. The tissue was then homogenized using a 30 ml Dounce homogenizer and incubated on ice for 5 min. 12.6 ml of 1.8 M Nuclei PURE Sucrose Mastermix (Nuclei Isolation Kit, Sigma-Aldrich, NUC201) was added to the suspension, which was then resuspended and overlaidon top of 7 ml of 1.8 M Nuclei PURE Sucrose Mastermix in polypropylene Beckman tubes (Open-Top Thinwall Polypropylene Tube, Beckman Colter, 326823). The gradient was centrifuged at 30’000 × *g* for 45 min at 4 °C to separate nuclei from myelin debris. The supernatant was discarded and the nuclei were resuspended in 1 ml of Nuclei PURE Storage Buffer (Nuclei Isolation Kit, Sigma-Aldrich, NUC201) and transferred into a 2 % BSA in PBS (Bovine Serum Albumin, Cytiva, SH30574.02) coated FACS polypropylene tube (Falcon Round-Bottom Polypropylene Test Tubes With Cap, Thermo Fisher Scientific, 352063) by filtering through a 30 μm strainer (MACS SmartStrainers 30 μm, Miltenyi Biotec, 130-098-462). The strainer was then washed with 1 ml of Nuclei PURE Storage Buffer four times. The suspension was then centrifuged at 500 × *g* for 5 min at 4 °C to pellet nuclei. Nuclei were resuspended in 2 ml FACS buffer (2 % BSA in PBS), divided equally into two new FACS tubes and incubated for blocking with anti- mouse TruStrain FcX PLUS CD16/32 antibody 1:10 (Biolegend, 156604) on ice for 15 min.

Antibodies used for nuclear staining and their working dilutions were as follows: anti- NeuN AF488 1:100 (for neuronal nuclei; Millipore MAB377X), anti- PU.1 -PE 1:50 (for microglia nuclei; Cell Signaling Technologies, 81886S), and anti- Olig2 -AF647 1:2000 (for oligodendrocytes nuclei; Abcam, ab225100), anti- LHX2/LH2 1:500 (for astrocytes nuclei; Abcam, ab219983), anti- rabbit IgG -AF647 1:1000 (Thermo Fisher Scientific, A21244).

One tube was stained for neurons, microglia, and oligodendrocytes makers, while the other for neurons and astrocytes markers. Staining lasted for 45 min on a rotating wheel at 4 °C. Nuclei were pelleted by centrifugation at 500 × *g* for 5 min at 4 °C, resuspended in 1 ml of FACS buffer and counterstained with Hoechst (1:2000) for 5 min at 4 °C. Nuclei were washed twice by centrifugation at 500 × *g* for 5 min at 4 °C and resuspension in 1 ml FACS buffer, and finally resuspended in 300 μl of FACS buffer before sorting. Sorting was performed following the gating strategy described by Nott et al. using a BD FACSAria III Cell Sorter (BD Biosciences)^91^.

### Single-cell RNA sequencing analysis

#### Prion-infected mouse dataset

We obtained the single-cell RNA sequencing dataset from prion-infected mice and controls from Slota et al.^25^. The data was accessed from the single cell portal at the Broad Institute: SCP1962. Upon importing the raw single-cell RNA sequencing data into R, the removal of doublets and ambient RNA was performed using scrublet^92^ and decontX^93^, respectively. Subsequently, cells with nFeature_RNA less than 1000 or more than 7000 or mitochondrial RNA percentage exceeding 5 %, were excluded through Seurat^94^. Following data normalization and regression of mitochondrial genes and cell cycle effects with Seurat, integration of data from different animals was achieved using Harmony^95^. Post data integration, cell clustering was performed using UMAP and the resulting clusters were annotated based on known cell-type markers.

For the phagocytic score (Fig. 5a), we utilized a previously published transcriptomic data where the authors injected apoptotic neurons into cortex and hippocampus of mice^46^. Next, they sorted two microglial population: P2ry12a^+^Clec7a^-^ (non-phagocytic) and P2ry12a^-^Clec7a^+^ (phagocytic) microglia. Sorted cells underwent bulk RNA sequencing analysis to identify phagocytic vs non-phagocytic markers. The dataset was processed to calculate log2 fold change (log2FC) values, defining phagocytic markers as those with log2FC > 1.5 and homeostatic markers as those with log2FC < − 1.5. To proceed, we selected a set of genes excluding those identified as unavailable in the microglial cluster from a published, prion-induced scRNAseq data^25^. We extracted expression data for these genes from a subset of microglial cells. Cluster identities were appended to this data and the data was transformed into a long format for visualization. Each gene was categorized as either phagocytic or homeostatic based on the established markers. We then quantified the activity of phagocytic and non-phagocytic markers by calculating mean expression scores for each marker type within each cell cluster. These scores were integrated into the metadata of the Seurat object. For each microglial cell, the average expression of phagocytic and non-phagocytic markers was determined and matched with cluster identities, enriching the Seurat object with phagocytic_score and non-phagocytic_score. Cell IDs were extracted and matched with their corresponding cluster information. Each gene expression data point was associated with a cell ID and categorized as either phagocytic or non-phagocytic. Mean scores for each cell were calculated separately for phagocytic and homeostatic markers and added as metadata in the Seurat object. Finally, we visualized the distribution of phagocytic and non-phagocytic scores across the microglial cell population using UMAP plots. Feature plots were created to color-code cells based on their phagocytic and homeostatic scores, highlighting regions of high and low activity.

#### 5XFAD mouse microglia dataset

We obtained the single-cell RNA sequencing dataset from sorted microglia from 5XFAD mice and controls from Keren-Shaul et al.^57^. The data was accessed from GEO database: GSE98969.

Filtering of the raw single-cell RNA sequencing data into R using the Seurat package was performed according to the original analysis: empty wells and cells with nCount_RNA less than 500 were excluded, then non-microglia myeloid contaminant cells were removed by filtering out cells that express monocyte markers *Ly6c2* and *Ccr2*, and perivascular macrophage markers *Mrc1*, *Cd163*, and *Lyve1*. Cell clustering and UMAP visualization was performed using Seurat.

### Cell linesculture

BV2 and CAD5 cells were cultured in DMEM/F12 (Gibco, Thermo Fisher Scientific) supplemented with 10% heat-inactivated fetal bovine serum (FBS, Cytiva), 1% GlutaMAX (Gibco), and 1% penicillin/streptomycin (P/S, Thermo Fisher Scientific). For lentiviral delivery, BV2 cells were seeded in antibiotic-free medium and placed under antibiotic selection 24 h after transduction.

GT1-7 cells were grown in OptiMEM (Gibco) supplemented with 10 % FBS, 1 % GlutaMAX, 1 % P/S and 1 % MEM Non-Essential Amino Acids (NEAA, Gibco). Stable infection of GT1-7 cells with mouse-adapted Rocky Mountain Laboratory sheep scrapie strain prions passage 6 (RML6) was achieved as previously described^96^. Briefly, cells were seeded in a 6-well plate and exposed to either 0.25 % (weight/volume) brain homogenate containing prions or to 0.25 % (w/v) non-infectious brain homogenate (NBH) as a control. Cells were incubated with infectious material for 3 days, followed by a full medium change. Cells were kept in culture for at least 8 passages to ensure persistent prion replication. Stably infected GT1-7 RML and NBH cells were seeded at 70% confluency and conditioned medium was collected after 3 days from plating.

For lentivirus packaging, HEK-293T cells were cultured in DMEM supplemented with 10% FBS.

### Generation of Gpnmb^-/-^ BV2 line

Genetic ablation of Gpnmb in BV2 cells was achieved using the CRISPR/Cas9 system. Briefly, BV2 cells were transduced with lentiCas9-blast plasmid (Addgene, plasmid # 52962) and, 24h later, selected with 10 μg/ml blasticidin (Thermo Fisher Scientific). BV2 cells stably expressing Cas9 were transduced with CRISPR guide RNA (gRNA) targeting mouse Gpnmb (GenScript, Gpnmb-1_pLentiGuide-Puro GenCRISPR Nickase gRNA Construct, SC1786) or non-targeting (NT) control (listed as Control_16 in ref. ^97^). 24 h after transduction, cells (referred to as “Gpnmb-depleted BV2 polyclonal”) were selected with 1.0 μg/ml puromycin (Thermo Fisher Scientific). A Gpnmb^-/-^ monoclonal line (referred to as “Gpnmb-depleted BV2 monoclonal”) was isolated via limited dilution, and complete knockout was confirmed via immunoblotting.

### Cell lines assays

For immunoblotting and qPCR experiments, BV2 cells were seeded in 6-well plates at 50’000 cells per well and treated the day after. For exposure to prion-infected mouse brain material, BV2 cells were treated with 0.5 % or 1 % (w/v) NBH and RML for 48 h. For prion-containing conditioned medium experiments, BV2 cells were treated with 1.5 ml/well of GT1-7 RML and NBH conditioned medium for 48 h. For treatment with NaPTA-precipitated RML or NBH material, BV2 cells were treated with an amount equivalent to 0.25 %, 0.5 % or 1 % (w/v) of the initial brain homogenate for 48 h. For the lipopolysaccharide (LPS) treatment, BV2 cells were administered with either 2 μg/ml LPS (*E.coli* O111:B4, Sigma-Aldrich) or the equivalent volume of PBS (Gibco) and then incubated for 24 h. For apoptotic bodies treatment and apoptotic bodies fraction treatment, BV2 cells were treated with 1.5 ml/well of conditioned medium from UV-irradiated or healthy cells and then incubated for 48 h. For Aβ PFFs treatment, BV2 cells were exposed to fibrils at monomer equivalent concentrations of 0 nM, 70 nM, 200 nM, 500 nM and then incubated for 48 h. Before harvesting for immunoblotting and qPCR, cells were washed once with PBS.

For viability assay, BV2 cells were seeded in clear-bottom 96-well plates (Revivity, ViewPlate-96 TC, 6005181) 4’000 cells per well and treated the day after. For Aβ PFFs treatment, BV2 cells were exposed to the same fibrils concentrations and incubated for 48 h. For apoptotic bodies treatment, BV2 cells were treated with 200 μl/well of conditioned medium from UV-irradiated or healthy cells and then incubated for 48 h. Cell viability was assessed using CellTiter Glo 2.0 (Promega, G9242) according to the manufacturer’s protocol and measured using GloMax Discover plate reader (Promega).

### iPSC culture

#### iNets generation and subculture

iNets^52^ were differentiated from iPSC-derived self-renewing human neural stem cell line (iCoMoNSCs)^54^ obtained from control human skin fibroblast, as described previously^52^. In brief, 600’000 iCoMoNSCs were plated onto Matrigel-coated 6-well plates (Corning, 354234) in NSC medium – DMEM/F12 medium (Gibco, 11330020); 0.5 x B27 supplement (Gibco, 12587-010); 0.5 x N2 supplement (Gibco, 17502-048); 1 x GlutaMAX (Gibco, 35050-061) and 25 ng/ml bFGF (Gibco, PHG0261) – and complete medium was changed daily until cells reached ~ 95 % confluency. At this stage, NSC medium was switched to D3 differentiation medium – DMEM/F12; 0.5 x B27 supplement, 1 x N2 supplement; 1 x GlutaMAX; 1 x Penicillin/Streptomycin (Sigma-Aldrich, P4333-100ML) – supplemented with 5 μM Forskolin (Cayman, AG-CN2-0089-M050), 1 μM synthetic retinoid EC23 (Amsbio, AMS.SRP002-2), 500 nM Smoothened agonist SAG (Millipore, 5666600) for a total of 5 days. On the days 6–10, synthetic retinoid EC23 was increased to 2 μM. On days 11–25, synthetic retinoid EC23 was decreased to 10 ng/ml, Smoothened agonist SAG to 50 nM, and 20 ng/ml BDNF (PeproTech, 450-02), 20 ng/ml GDNF (PeproTech, 450-10) and 20 ng/ml CNTF (Alomone labs, C-240) were added. From day 26 and onwards, medium was switched to maturation medium – 1:1 DMEM/F12:Neurobasal (Gibco, 21103049) mix; 1 x B27 supplement, 1 x N2 supplement; 1.5 x GlutaMAX – supplemented with 5 μM forskolin, 20 ng/ml BDNF, 20 ng/ml GDNF, 20 ng/ml CNTF, 20 ng/ml NT-3 (PeproTech, 450-03), 20 ng/ml IGF-1 (Stem Cell, 78022), and 10 μM cAMP (Sigma-Aldrich, D0260). One-third of the medium was changed daily between days 0 and day 10, whereas from this point on only two-thirds of the medium was changed 3 times a week. 2-month old iNets were dissociated into a single-cell suspension using Papain Dissociation System (Worthington, LK003150), passed through a 70 μm cell strainer (Falcon, 07-201-431), resuspended in supplemented maturation medium and re-plated (120’000 cells/well) into μ-Plate 96 Well Black (Ibidi, 89626).

#### Macrophage precursors generation and integration in iNets

Macrophage precursors were generated starting from the KOLF2.1J parental line provided by the iPSC Neurodegenerative Disease Initiative (iNDI) from the NIH’s Center for Alzheimer’s and Related Dementias (CARD), adapting the protocol from van Wilgenburg et al., 2013^53^. In short, iPSCs were seeded into AggreWell 800 plates (STEMCELL Technologies, 34811) pre-treated with Anti-Adherence Rinsing Solution (STEMCELL Technologies, 07010) at a density of 120’000 cells/well to initiate embryoid bodies (EBs) formation. Cells were cultured in StemFlex medium (STEMCELL Technologies, A3349401) supplemented daily with 50 ng/mL BMP4 (Miltenyi Biotec, 130-111-165), 50 ng/mL VEGF (Gibco, PHC9391), and 20 ng/mL SCF (CellGenix, 1418-050). After four days, EBs were transferred into T75 flasks (Sigma-Aldrich) at approximately 75 EBs per flask and cultured in X-VIVO 15 medium (Lonza, BE02-060F) supplemented with 100 ng/mL M-CSF (Invitrogen, 130-096-492), 25 ng/mL IL-3 (Peprotech, 200-03), 2 mM GlutaMAX, 1x Penicillin/Streptomycin, and 0.055 mM β-mercaptoethanol (Invitrogen, 31350010). Medium was changed twice a week. After 5 weeks of factories set-up, mature macrophage precursors (pMacpre) emerging in the supernatant were collected and cultures replenished with fresh EBs medium. Harvested pMacpre were then strained through a 40 μm cell strainer (Falcon) and centrifuged at 200 × *g* for 5 min at room temperature. Cells were resuspended in iNets maturation medium supplemented with 100 ng/mL IL-34 (Peprotech, 200-34) and plated on top of previously subcultured iNets (30’000 cells/well) into μ-Plate 96 Well Black (Ibidi, 89626). Two-thirds of the medium was replaced three times per week over 15 days to promote the maturation of pMacpre into microglia-like cells (pMGL).

### Generation of inducible EGFP iNets

Lentiviral transfect vector for the inducible expression of EGFP was generated from the previously described all-in-one monocistronic TetON plasmid^52^ by replacing the TDP-43-HA with the EGFP sequence via HIFI kit (New Englad Biolabs, E5520S). iNets were transduced with lentivirus with GoStix Value of 1000 ng/ml p24 in neural maturation medium 18 days after subculturing and 15 days after macrophage precursors addition to the neural network. The day after transduction, complete medium was exchanged to neural maturation medium containing 1 μg/ml doxycycline (Clontech, 631311) to induce transgene expression. Doxycycline was included in subsequent medium refreshments every other day for the following 12 days, when the cultures were fixed for analysis.

### Immunofluorescence and quantification of iNets and pMGL

For LPS and Aβ fibrils treatment, iNets and pMGL were treated with either 2 μg/ml LPS (*E.coli* O111:B4, Sigma-Aldrich), Aβ PFFs fibrils at a monomer equivalent concentration 70 nM, 200 nM, 500 nM, or the equivalent volume of PBS (Gibco) and then incubated for 48 h.

iNets and pMGL were fixed with pre-warmed 16% formaldehyde (Pierce, 28908) added directly into the culture medium to 4% final concentration, and incubated for 20 min at room temperature. Cells were then washed once with PBS (Gibco) for 10 min, permeabilized with PBS with 0.2% Triton X-100 (Sigma-Aldrich) for 10 min, and then blocked with filter-sterilized (Stericup vacuum filter, Millipore, S2GPU02RE) 10% normal donkey serum (Sigma-Aldrich) in PBS 0.2% Triton X-100 for 1 h. Primary antibodies were diluted in blocking solution (see below) overnight at 4 °C on an orbital shaker. The samples were washed in PBS three times before and after staining with secondary antibodies and DAPI 1 μg/ml (Thermo Fisher Scientific, 62248) for 1 h at room temperature. Finally, stained cells were stored in PBS at 4 °C.

Primary antibodies used and their working dilutions were as follows: anti-MAP2 1:1000 (chicken, Abcam, ab5392), anti- IBA1 1:500 (rabbit, Abcam, ab178846), anti-GFP 1:1000 (mouse, Proteintech, 66002-1-IG), and anti-GPNMB 1:200 (Biotechne, AF2550). Secondary antibodies used and their working dilutions were as follows: anti-goat IgG AF405 1:1000 (Abcam, ab175664), anti- rabbit IgG AF568 1:1000 (Invitrogen, A10042), anti-chicken IgG AF647 1:1000 (Invitrogen, A78952).

Stained cells were imaged using high content laser scanner confocal microscope (Molecular Devices ImageXpress Confocal HT.ai). Microscope settings were the following: 40x Water Apo LambdaS LWD objective; 50 μm spinning disk; 2048 x 2048 pixels; 30 z-steps per stack and 0.3 μm step size.

The acquired images were analyzed using custom macros written to suit the FIJI distribution of ImageJ2^98^. To facilitate cell segmentation of pMGLs and iNets, several computations were performed on images capturing IBA1 and MAP2 antibody signals, respectively. First, smoothing via Gaussian blur was performed to reduce noise. Subsequently, background subtraction and the use of an automated intensity threshold enabled the capture of cell candidates in a generated image mask. Moreover, brightness and contrast settings were made uniform for all MAP2 and IBA1 images, respectively. Finally, the segmented regions of interest (ROIs) were filtered using criteria tailored to exclude non-cell objects via filters for size and degree of circularity. Using the resulting mask, average fluorescent intensities were computed per ROI via a second automated script. While only MAP2 signals were used to capture iNets intensities, pMGL intensities were derived from the GPNMB and IBA1-signal channels, both by usage of the IBA1-derived ROIs. This ensured that GPNMB-negative pMGLs were also considered within the image analysis. Furthermore, the GPNMB intensity average per each ROI were divided by the corresponding IBA1 intensity average for normalization, ensuring that technical variation during image acquisition was accounted for.

### Lentivirus production

HEK-293T cells were seeded at 60% confluency. 24 h later, the cargo plasmid was co-transfected together with pCMV-VSV-G (Addgene, plasmid #8454) and psPAX2 (Addgene, plasmid #12260) plasmids using Lipofectamine 3000 transfection reagent (Invitrogen). 6 h after transfection, the medium was replaced with DMEM supplemented with 10% FBS and 1% Bovine Serum Albumin (Sigma-Aldrich). 3 days after transfection, supernatant was harvested, centrifuged at 1500 × *g* for 5 min, filtered through 0.45 μm strainer (Whatman), aliquoted and stored at − 80 °C.

For iNets transduction, lentiviral vector mTRE-EGFP was generated, harvested and concentrated as described previously^52^. Lentiviral pellet was resuspended in neural maturation medium containing all supplements except forskolin and cAMP, to obtain 10x concentrated preparations. Lentiviral titer was assessed using Lenti-X GoStix Plus (Takara, 631280). Lentiviral preparations were aliquoted and stored at − 80 °C.

### Generation of apoptotic bodies

To generate apoptotic bodies, CAD5 or BV2 cells were seeded at 60 % confluency. 12 h after seeding, cells were irradiated with UV light (40’000 J/m^2^ using Stratagene UV crosslinker) for 24 h. Cells were then incubated at 37 °C for 24 h, and the apoptotic bodies-containing medium gently collected and used immediately. A twin flask of healthy CAD5 or BV2 cells at matching confluency was used as a control. Presence of apoptotic bodies in the medium was confirmed via flow cytometry. The same protocol to generate apoptotic bodies was employed for the phagocytosis assay.

The apoptotic bodies isolation protocol was adapted from Phan et al., 2018^99^. Briefly, apoptotic bodies from CAD5 cells were generated as described above. After the 24 h incubation at 37 °C, apoptotic bodies were mechanically detached via pipetting, and the cell suspension was centrifuged at 3000 × *g* for 6 min. Supernatant was collected as an apoptotic bodies-depleted conditioned medium (referred to as “Conditioned Medium”). The pellet was resuspended in 2 ml cold PBS and centrifuged at 200 × *g* for 6 min. The top 1 ml supernatant was collected as apoptotic bodies fraction (referred to as “Apoptotic bodies enrichment”) and resuspended in fresh growth medium. A twin flask of healthy CAD5 cells at matching confluency was used as a control and underwent the same isolation protocol. Successful isolation of apoptotic bodies was confirmed via flow cytometry.

### Prion precipitation and purification

Prions were purified from 10 % (w/v) RML or NBH mouse brain homogenate as described by Wenborn et al. 2015^51^. The following reagents and materials were employed: Sodium phosphotungstic acid (NaPTA) (Sigma-Aldrich), OptiPrep density gradient medium (STEMCELL Technologies), Protease type XIV from *Streptomyces griseus* (Sigma-Aldrich), benzonase nuclease (Sigma-Aldrich), EDTA 0.5 M, pH 8.0, RNA-free (Invitrogen), Sodium lauroylsarcosine (Sigma-Aldrich), Dulbecco's phosphate-buffered saline (D-PBS) lacking Mg^2+^ and Ca^2+^ (Gibco), Ultrafree-MC microcentrifuge filtration unit (Millipore, UFC30HV00). Successful prion isolation was confirmed via silver staining and Western blot.

### Aβ PFFs preparation

Aβ PFFs were prepared as described by Stine et al., 2003^100^. In brief, lyophilized recombinant Aβ_1-42_ monomers were reconstituted in 100 % hexafluoro-2-propanol (HFIP; Sigma-Aldrich). Then, HFIP was removed by evaporation, and the peptide film reconstituted in DMSO (Sigma-Aldrich) at a final concentration of 5 mM and sonicated in a water bath sonicator (MRC laboratory instruments) for 10 minutes to ensure complete dissolution. To prepare fibrils, Aβ monomers was diluted to 100 μM in 10 mM HCl, filtered using 0.22 μm filters (Whatman), and incubated at 37 °C with 600 rpm shaking at for 24 h. Finally, fibrils were dialyzed in PBS (Gibco) using Slide-A-Lyzer MINI with a molecular weight cutoff of 3.5 KDa (Thermo Fisher Scientific, 88400). Presence of Aβ PFFs was confirmed by transmission electron microscopy.

### Flow cytometry

To assess the presence of apoptotic bodies in the conditioned medium, apoptotic marker phosphatidyl serine was stained using fluorescently labeled annexin-V (BD biosciences, 556421). Briefly, 5 μl of PE-annexin V were added to 100 μL of conditioned medium from UV-treated and healthy CAD5 conditioned medium. After 15 min of incubation in the dark, samples were washed twice with PBS and resuspended in FACS buffer. Samples were acquired at the LSRFortessa flow cytometer (DB biosciences). The same protocol was used to confirm apoptotic bodies isolation.

For the phagocytosis assay, UV-treated CAD5 cells were conjugated to pHrodo fluorescent dye (pHrodo red SE, Invitrogen) according to the manufacturer’s protocol. Briefly, cells were detached through mechanical pipetting and resuspended at 10^6^ cells/ml in PBS. Substrates were incubated in 1 mM pHrodo for 2 h at room temperature in the dark. After conjugation, substrates were washed three times in PBS and used immediately. BV2 cells (100’000 cells/well in 24-well plate) were treated with 100 μl pHrodo-labeled CAD5 apoptotic bodies (around 100’000 apoptotic cells) and incubated for 1.5 h and 3 h at 37 °C. After incubation, samples were washed twice with PBS and resuspended in FACS buffer. 10’000 events were acquired per sample using LSRFortessa flow cytometer (DB biosciences). Phagocytosis was quantified as percentage of pHrodo^+^ cells compared to untreated sample. The experiment was performed three times, each one in three technical replicates. Each dot in the graph represents the average of 3 technical replicates. All flow cytometry data were analyzed using FlowJo 10 (Tree Star).

### Immunoblotting and silver staining

Mouse samples were homogenized twice at 5000 rpm for 15 s in 1 ml of cell-lysis buffer (20 mM Hepes-KOH, pH 7.4, 150 mM KCl, 5 mM MgCl_2_, 1 % IGEPAL) supplemented with cOmplete mini protease inhibitors (Roche) using a Precellys24 Sample Homogenizer (LABGENE Scientific SA, BER300P24). After 20 min incubation in ice cleared lysates were obtained by centrifugation at 2000 × *g*, 4 °C for 10 min.

Cell extracts and NaPTA-precipitated samples were prepared in lysis buffer (50 mM Tris–HCl pH 8.0, 150 mM NaCl, 0.5 % sodium deoxycholate and 0.5 % Triton-X 100) supplemented with cOmplete mini protease inhibitors (Roche). In the case of proteinase K (PK) (Roche) digestion, proteinase inhibitors were avoided.

Total protein concentration was measured using bicinchoninic acid assay (BCA) according to the manufacturer’s protocol (Pierce). Western blots were performed using standard procedures. Briefly, samples were boiled at 95 °C in 1X NuPAGE LDS sample buffer (Invitrogen) supplemented with 1 mM DTT (Sigma-Aldrich), loaded into NuPAGE Bis-Tris precast PAGE gels (Invitrogen) and transferred on PVDF membranes (Invitrogen).

For NaPTA-precipitated prions, Proteinase K digestion was performed at 10 μg/ml for 1 h at 37 °C. For silver staining, loading of NaPTA-precipitated samples was equivalent to 100 μl of 10 % (w/v) of the initial brain homogenate. As control, 2 μl of 10 % (w/v) normal NBH and RML brain homogenate were loaded. Silver staining was performed using the SilverXpress kit according to the manufacturer’s instructions (Invitrogen).

Antibodies used for immunoblotting and their working dilutions were as follows: anti- Gpnmb 1:1000 (Bio-Techne, AF2330), anti- Lgals3 1:2000 (Abcam, ab2785), anti- Spp1 1:3000 (Abcam, ab11503), anti- Vim 1:3000 (Abcam, ab92547), anti- Atp6v0d2 1:2000 (Novus Biologicals, NBP3-10978), anti- NeuN 1:1000 (Abcam, ab177487), anti- Vglut1 1:1000 (Abcam, ab77822), anti- Vgat 1:500 (Santa Cruz Biotechnology, sc-393373), anti- Olig2 1:1000 (Abcam, ab109186), anti- Iba1 1:1000 (Wako, 016-20001), anti- Gfap 1:10’000 (Dako, Z0334), anti- Syp 1:1000 (BD Biosciences, #611880), anti- PrP POM1 300 ng/ml^101^, anti- Vinculin 1:5000 (Abcam, ab129002), anti- Actin -HRP 1:10’000 (Sigma-Aldrich, A3854), anti- Rabbit IgG -HRP 1:10’000 (Jackson ImmunoResearch, 111.035.045), anti- Mouse IgG -HRP 1:10’000 (Jackson ImmunoResearch, 115.035.003), anti- mouse IgM -HRP 1:1000 (Zymed, 61-6420), anti- Goat IgG -HRP 1:10’000 (Jackson ImmunoResearch, 705.035.147).

### qPCR sample preparation

For CJD patient samples, tissues were lysed in TE buffer (Invitrogen) with the anionic detergent sodium dodecyl sulfate (SDS; Invitrogen) and digested at 50 °C with 2 mg/ml PK (Roche) for 2 h to eliminate solids and release nucleic acids from proteins. Then, TRIzol (Invitrogen) reagent solution was added to the lysate and kept overnight at 4 °C. Guanidine thiocyanate (Gdn-SCN), a chaotropic salt present in TRIzol, denatures proteins and abolishes prion infectivity. At high concentrations, guanidine salts disaggregate PK-resistant PrP^Sc^ fibrils, eliminate PK resistance, and prevent further PrP^Sc^ conversion. Any PK-resistant material that survived the digestion step is inactivated at this stage of the protocol. Subsequently, 0.2 ml ultrapure phenol:chloroform:isoamyl alcohol (Thermo Fischer Scientific) was added to the samples, followed by vigorous shaking and incubation for 5 min at room temperature. Samples were centrifuged step at 12’000 g for 15 min at 4 °C to separate the two phases. The aqueous upper phase was transferred to a fresh tube, mixed with 0.5 ml isopropanol (Sigma-Aldrich) and 1 μl Glycoblue Coprecipitant (Thermo Fisher Scientific), and centrifuged at 12,000 × *g* for 20 min at 4 °C to pellet RNA. The pellet was washed twice with 75 % ethanol. The RNA pellet was dissolved in 11 μl nuclease-free water (Invitrogen, AM9930) at 55 °C.

For mouse samples, tissue was homogenized in TRIzol using a Precellys24 Sample Homogenizer (LABGENE Scientific SA, BER300P24) for 5 min at 50 oscillations/s and incubated overnight at 4 °C. After equilibration to room temperature, 400 μl chloroform (Sigma-Aldrich) was added, samples were vigorous mixed, incubated for 2 min, and centrifuged at 16,000 × *g* for 30 min at 4 °C. The aqueous upper phase was transferred to a new tube, and 0.5 μl GlycoBlue Coprecipitant (Thermo Fisher Scientific) and 500 μl isopropanol (Sigma-Aldrich) were added. Following vortex mixing and 10 min incubation at room temperature, RNA was pelleted by centrifuging at 16,000 × *g* for 30 min at 4 °C. The supernatant was discarded, and the pellet was washed twice with 75 % ethanol (centrifugation at 16,000 × *g* for 10 min at 4 °C), and dissolved in 11 μl RNase-free water (Invitrogen, AM9930) for 10 min at 65 °C.

For BV2 cells, RNA was extracted using the RNeasy kit (Qiagen) according to the manufacturer’s instructions.

RNA concentration was measured with a NanoDrop spectrophotometer (Thermo Fisher Scientific). Reverse transcription was performed using the Quantitect Reverse Transcription kit (Qiagen) following the manufacturer’s instructions. For qPCR, 10 ng of cDNA per sample was loaded in triplicate into 384-well PCR plates (Life Systems Design), and the amplification was performed with SYBR green mastermix (Roche). Reactions were run with ViiA7 Real-Time PCR systems (Thermo Fisher Scientific). RT-qPCR data was analyzed using the 2^-ΔΔCT^ method.

Primer sequences: mouse *Gpnmb* (fw: 5'-TCT GAA CCG AGC CCT GAC ATC-3', rev: 5'-AGC AGT AGC GGC CAT GTG AAG-3'), human *GPNMB* (fw: 5’-ACT GTT GCT CTT GGT GGA CG-3’, rev: 5’-CCA GGA GCA GAA ATC CCA GG-3’), mouse *Gapdh* (fw: 5’-CCA CCC CAG CAA GGA GAC-3’, rev: 5’-GAA ATT GTG AGG GAG ATC CT-3’), human *GAPDH* (fw: 5’-TGC ACC ACC AAC TGC TTA GC-3’, rev: 5’- GGC ATG GAC TGT GGT CAT CAG-3’).

### Sandwich ELISA

For Gpnmb ELISA measurements in murine samples, high-binding 384-well plates (Perkin Elmer, SpectraPlate 384 HB) were coated with 1 μg/ml of anti- Gpnmb antibody (Bio-Techne, AF2330) in PBS overnight at 4 °C. Plates were washed three times with PBS-T (0.2 % Tween20 (Sigma-Aldrich) in PBS) and blocked with 5 % SureBlock (LubioScience, SB232010) in PBS-T for 1 h at RT. The murine CSF (1:20), blood serum (1:5) and recombinant mouse Gpnmb standard (1.9 × 10⁻^6^ μg/ml; Bio-Techne, 2330-AC) were diluted with 1 % SureBlock PBS-T and run in technical duplicates. After 2 h at RT, plates were washed five times with PBS-T and incubated with 1 μg/ml of biotinylated anti- Gpnmb antibody (Bio-Techne, BAF2330) in 1 % SureBlock PBS-T for 1 h at RT. Then plates were washed three times with PBS-T followed by incubation with streptavidin-HRP 1:1000 (Biolegend, 405210) in 1 % SureBlock PBS-T for 30 min at RT. After three washes with PBS-T, TMB (Invitrogen, 10354603) was added for 5 min, the reaction was stopped with 0.5 M sulfuric acid, and absorbance at 450 nm was measured (PerkinElmer, Envision).

For GPNMB ELISA measurements in human samples, plates were coated with 1 μg/ml of anti-GPNMB antibody (Bio-Techne, AF2550) and processed as above. sCJD patient CSF (1:20), blood serum (1:40) and standard (1.9 × 10⁻^6 ^μg/ml; Bio-Techne, 2550-AC) were run in technical triplicates. For detection, we used 1 μg/ml biotinylated anti-GPNMB antibody (Bio-Techne, BAF2550) diluted in 1 % SureBlock PBS-T, streptavidin-HRP 1:1000 (Biolegend, 405210) in 1 % SureBlock PBS-T Absorbance at 450 nm was measured FLUOstar Omega plate reader (BMG Labtech).

### Statistical analysis

Variance of densitometric analysis of all Western blot was plotted as mean ± standard deviation (s.d.). For Supplementary Figs. 6b, 9b, 11d, 12d, 13b, 13d–f, 14c, and 16f statistical significance was assessed using two-sided Welch’s* t* tests. For Supplementary Fig 16c, significance was evaluated by two-sided one-way ANOVA followed by Dunnett’s multiple comparison test.

Variance of all qPCR experiments was plotted as mean ± s.d. For Fig. 3c, and Supplementary Fig 12a, statistical significance was assessed using two-sided Welch’s *t* tests. For data that deviated from normality, such as in Fig. 2c, the two-sided Wilcoxon rank-sum test was employed.

For ELISA analyses (Fig. 2d, e), OD values variance was visualized as mean ± s.d., and statistical significance was assessed using the Wilcoxon rank-sum test.

For cell viability analysis, variance of cell viability assays was plotted as mean ± s.d.. Statistical significance of Supplementary Fig 16d was assessed by two-sided one-way ANOVA followed by Dunnett’s multiple comparison test, and Supplementary Fig 16g by two-sided Welch’s *t* tests.

For phagocytosis assay analysis (Fig. 6e), data are presented as mean ± s.d. and statistical significance was assessed using two-sided one-way ANOVA followed by Dunnett’s multiple comparison test.

For cerebral blood flow measurement (Fig. 6g), data variance was plotted as mean ± s.d., and significance was assessed using two-sided Welch’s *t* tests.

For immunofluorescence experiments, co-localization experiments (iPSC-derived cells: Fig. 6c, and Supplementary Fig. 15b, 17c, d; brain slices: Supplementary Fig. 10c), data distribution was visualized by means of box plot: the central line indicates the median, the box shows the interquartile range (25th–75th percentiles), and whiskers extend to the most extreme values within 1.5x the interquartile range. For brain slices co-localization analysis in Fig. 6h, variance was plotted as mean ± s.d. In all cases, pairwise comparisons between experimental conditions were performed using two-sided Wilcoxon rank-sum tests. For panels with multiple comparisons (Supplementary Figs. 15b, 17c, d, and Fig. 6c), *p*-values were adjusted using the Benjamini-Hochberg method.

For the mice weight analysis (Supplementary Fig. 9c), the mean of body weights for each condition were calculated across weeks to track weight trends over time. Two-sided Welch’s *t* tests were performed at each time point to determine statistical significance.

In the analysis of overlapping genes between conditions (Fig. 3a and Supplementary Fig. 1b), statistical significance was evaluated using Fisher’s exact test.

For differential expression analysis (Figs. 1b and 5b)^94^, which models raw RNA-seq counts using a negative binomial generalized linear model and test significance using a two-sided Wald test. *P*-values were adjusted using the Benjamini–Hochberg method to control the false discovery rate (FDR), and genes with adjusted *P *< 0.05 were considered significant. Log2 fold change values indicate the magnitude of expression differences.

For over-representation analysis of GO terms (Fig. 3b and Supplementary Fig. 2) and KEGG terms (Supplementary Fig. 10a), Fisher’s exact test was used to identify enriched terms, with p-values adjusted using the Benjamini–Hochberg method to control FDR.

### Reporting summary

Further information on research design is available in the Nature Portfolio Reporting Summary linked to this article.

## Supplementary information

Supplementary information

Description of Additional Supplementary Files

Supplementary Data 1

Supplementary Data 2

Supplementary Data 3

Supplementary Data 4

Supplementary Data 5

Supplementary Data 6

Supplementary Data 7

Supplementary Data 8

Supplementary Data 9

Reporting summary

Transparent Peer Review file

## Source data

Source data 1

Source data 2

## Data Availability

The spatial transcriptomics data, as well as related processed data generated in this study, have been deposited in the GEO database under accession code GSE277577. Raw immunofluorescent microscopy images have been deposited in the Zenodo database 10.5281/zenodo.18743243. The single-cell RNA-seq dataset reanalyzed in this study^25^ is available from the Broad Institute Single Cell Portal under study SCP1962. The single-cell RNA sequencing dataset from sorted microglia from 5XFAD mice and controls^57^ is available at GSE98969. The spatial transcriptomics data presented in Fig. 6i, was obtained through the online platform strokemap.cn. The data values generated in this study are provided in the Source Data file. Source data are provided in this paper.

## Code availability

Code used to reproduce the results reported in this study is available at https://github.com/dcare91/ST_prions and archived at Zenodo 10.5281/zenodo.18983911. The STdeconvolve algorithm used in this study is available at https://github.com/JEFworks-Lab/STdeconvolve.git.
