# Supplementary material for: Gpnmb defines a phagocytic state of microglia linked to cell death in prion disease mouse model
Source: Nat Commun. 2026 May 12;17:6138. doi: 10.1038/s41467-026-73003-5 (PMC13365222; doi:10.1038/s41467-026-73003-5)
Supplement: Supplementary file 1 — Supplementary information [file 41467_2026_73003_MOESM1_ESM.docx]

**Gpnmb Defines a Phagocytic State of Microglia Linked to Cell Death in Prion Disease Mouse Model**

Supplementary Information


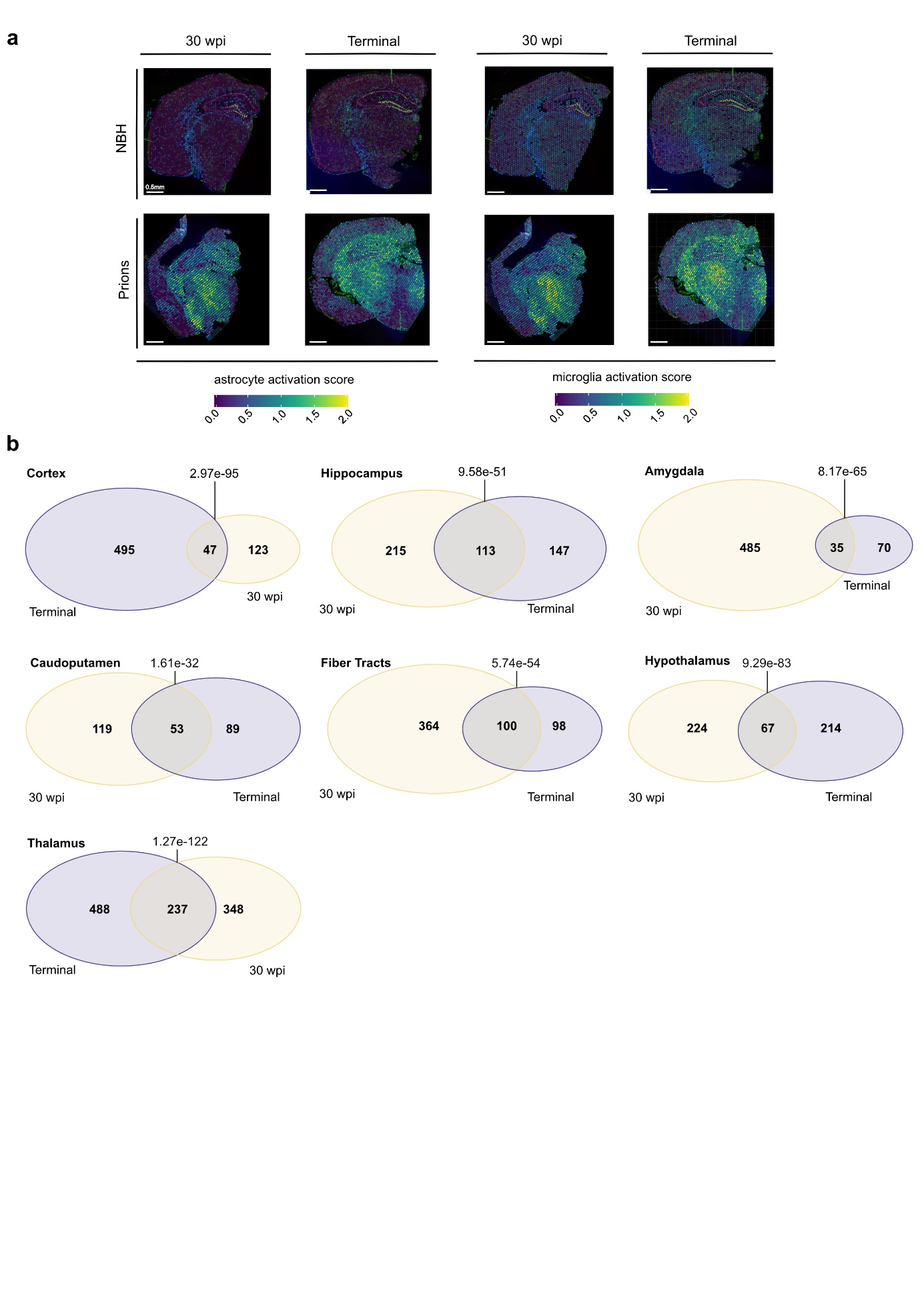


**Supplementary Figure 1. Glial activation scores and DEGs overlap between different timepoints for each brain region. a)** Panels display results from a 10x Genomics spatial transcriptomic assay, comparing brain slices under two conditions (NBH and Prions) at two time points (30 weeks post-infection (wpi) and Terminal). These images are color-coded to illustrate levels of astrocyte and microglia activation, with activation scores shown at the bottom of the panel. The activation levels are indicated by the color spectrum, where cooler colors (blues) represent lower activation and warmer colors (yellows) indicate higher activation. Scale bars represent 0.5 mm. **b)** This figure illustrates the overlap of differentially expressed genes (DEGs) between two critical disease stages - 30 weeks post-inoculation (wpi) and the terminal stage - across various brain regions including the cortex, hippocampus, amygdala, caudoputamen, fiber tracts, thalamus and hypothalamus. Each Venn diagram represents a specific brain region, detailing the number of unique and shared DEGs at each stage. Overlap significance was tested with Fisher’s Exact Test.


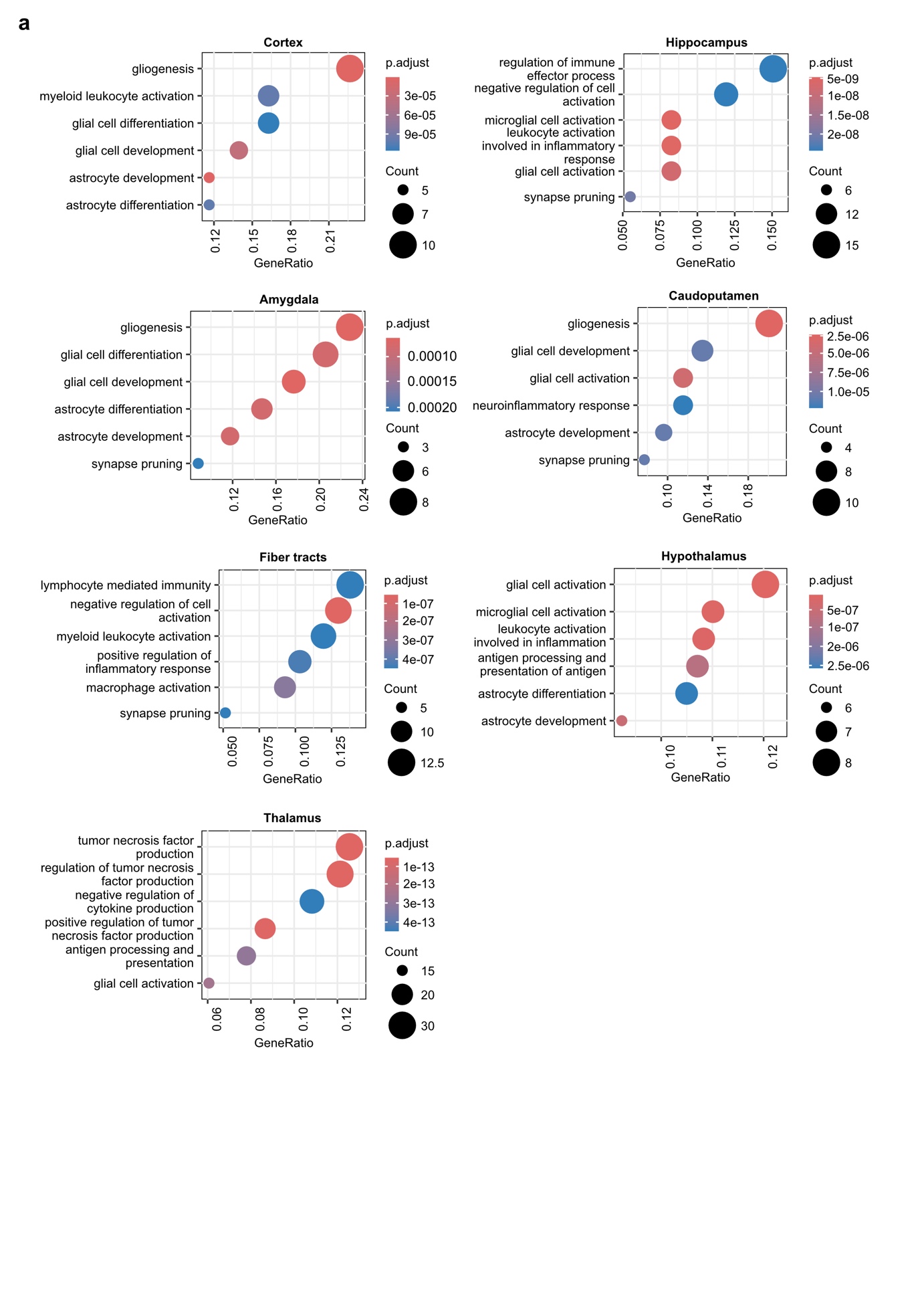


**Supplementary Figure 2.** **Over-representation analysis of overlapping DEGs for each brain region.** Over-representation analysis of genes that overlap between the 30 weeks post-inoculation and terminal stages of PrD across cortex, hippocampus, amygdala, caudoputamen, fiber tracts, hypothalamus and thalamus. Circles represent enriched biological processes; color indicates adjusted p-values and size reflects genes count. Significance was assessed with Fisher’s Exact Test, and p-values were adjusted using Benjamini–Hochberg method to control FDR


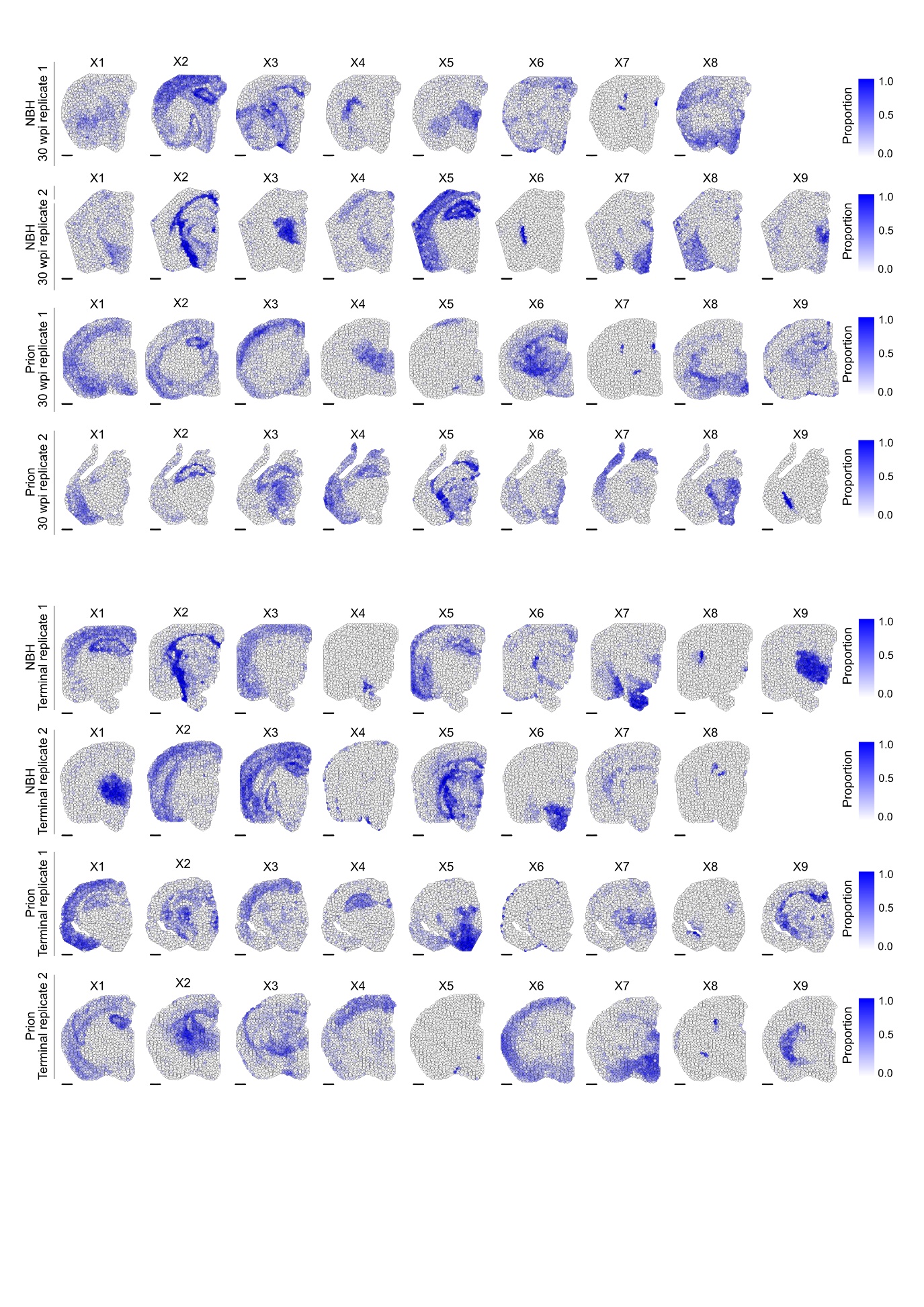


**Supplementary Figure 3.** **Spatial distribution of deconvolved gene expression profiles.** Spatial distribution and relative abundance of various gene expression profiles in brain sections from both control (NBH) and prion-infected samples at 30 wpi and the terminal stages. Each row corresponds to a different sample, mapping the distribution of identified gene expression profiles from X1 to X9. The intensity of the color within each brain section indicates the proportion of specific cell types, with darker shades representing a higher prevalence. Scale bars represent 0.5 mm.


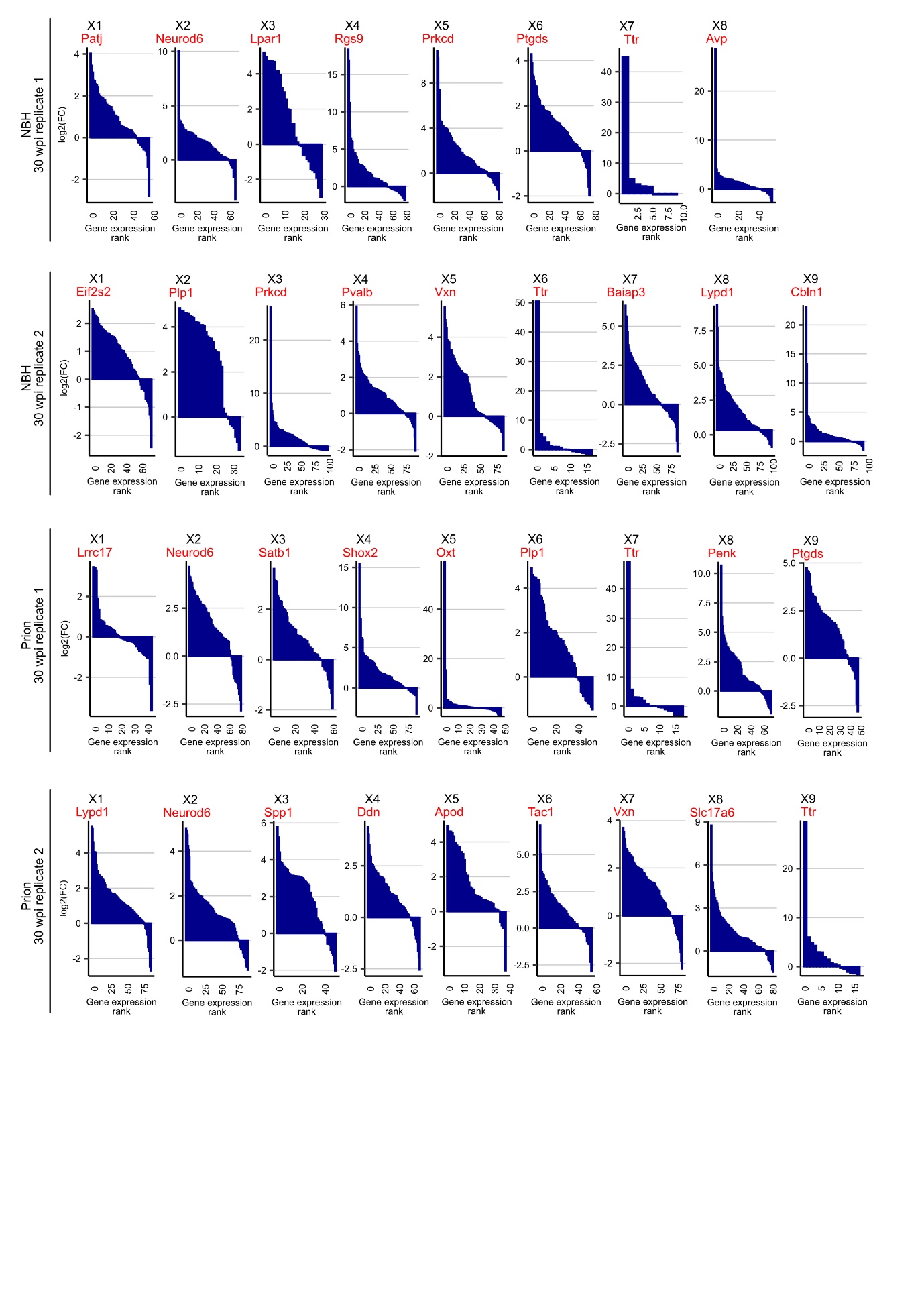


**Supplementary Figure 4.** **Deconvolved gene expression profiles at 30 wpi.** Ranked gene expression profiles (X1 – X9) inferred by STdeconvolve algorithm from control (NBH) and prion-infected samples at 30 wpi. Rows correspond to experimental conditions and replicates. The x-axis shows the rank of genes, and the y-axis depicts the log2 fold change. The plots are labelled after the most enriched gene (in red) within each profile.


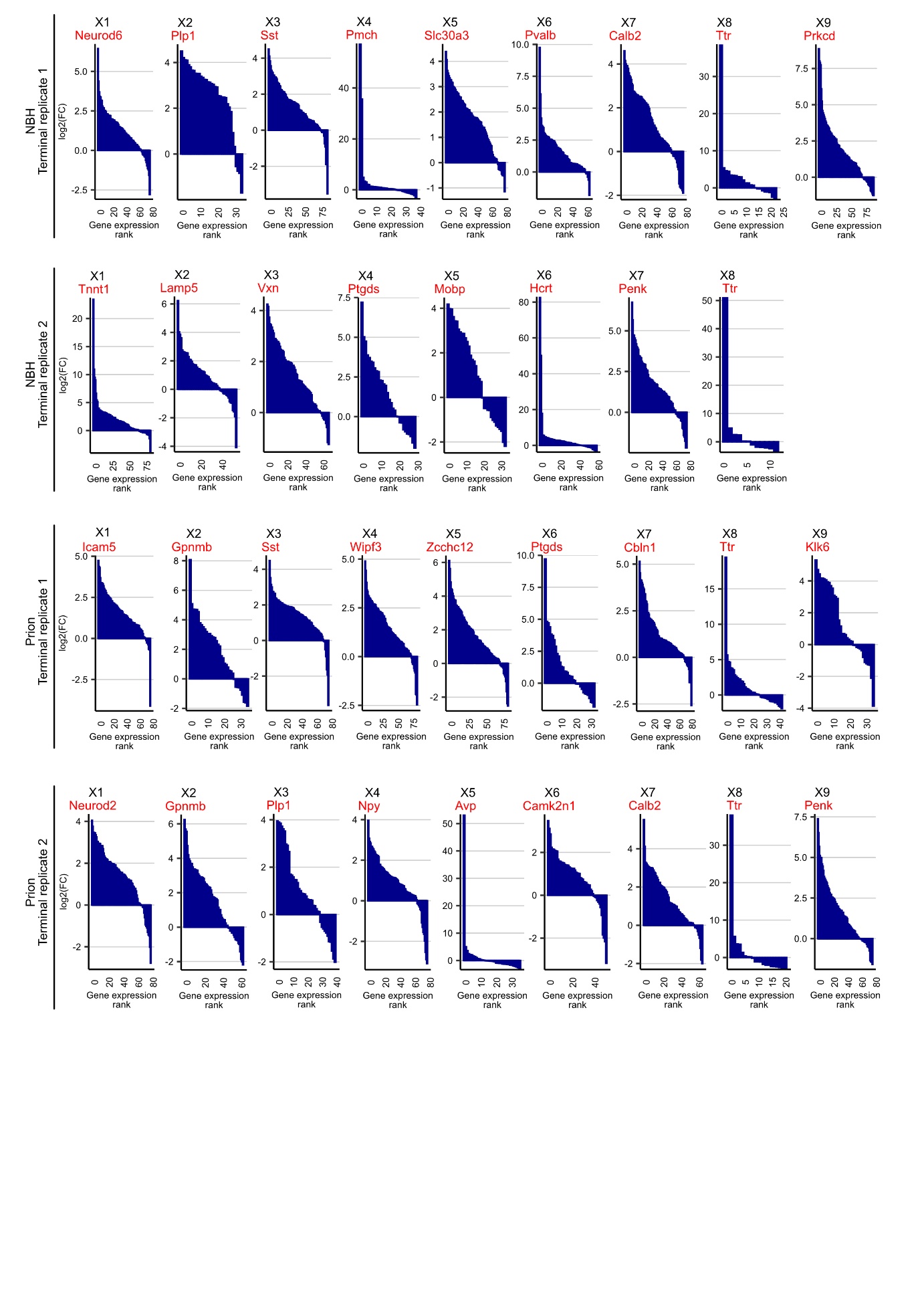


**Supplementary Figure 5.** **Deconvolved gene expression profiles at terminal stage.** Ranked gene expression profiles (X1 – X9) inferred by STdeconvolve algorithm from control (NBH) and prion-infected samples at terminal stage. Rows correspond to experimental conditions and replicates. The x-axis shows the rank of genes, and the y-axis depicts the log2 fold change. The plots are labelled after the most enriched gene (in red) within each profile.


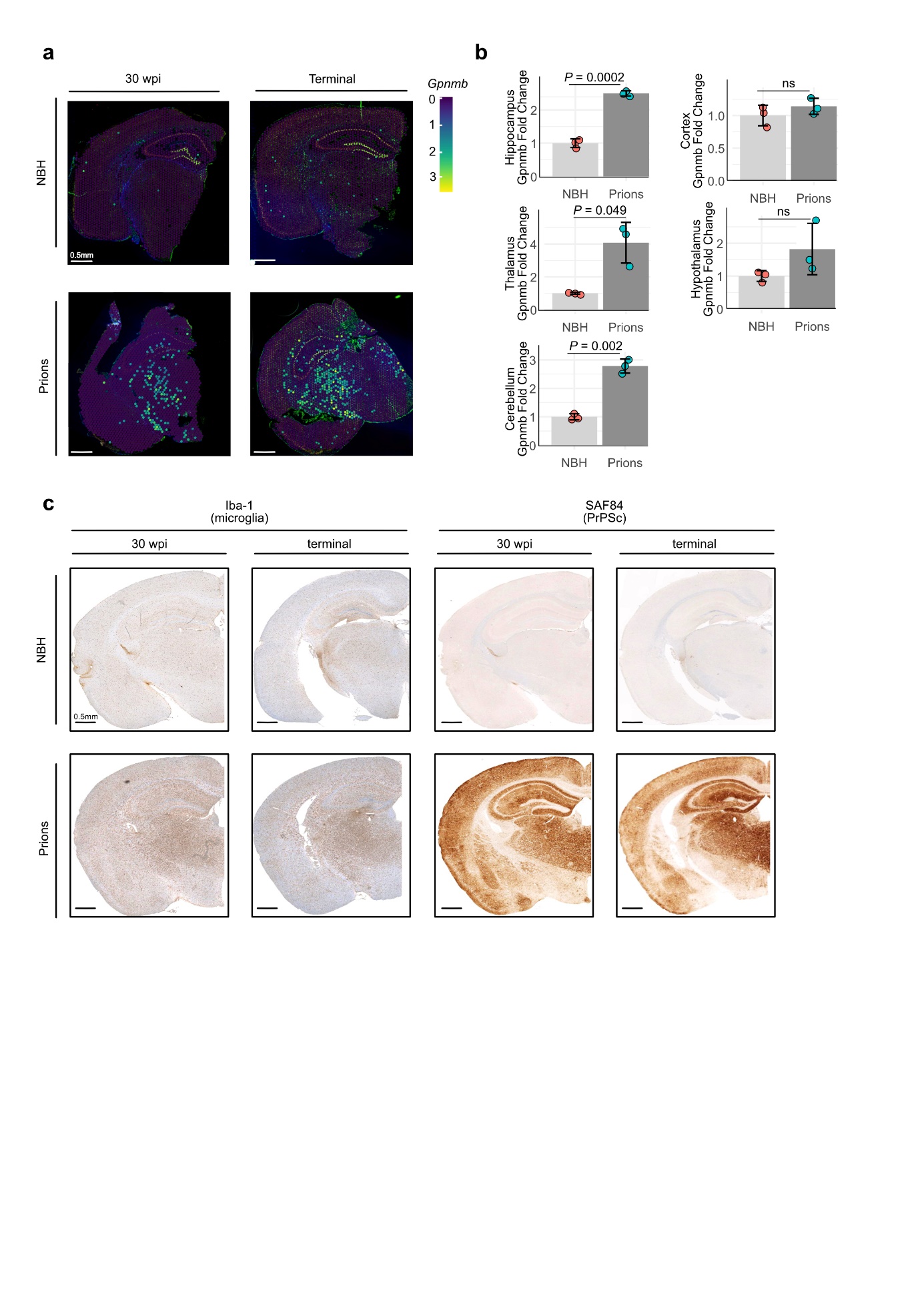


**Supplementary Figure 6.** **Spatial Gpnmb expression in region of intense microgliosis and prion accumulation.** **a)** Spatial transcriptomic maps showing Gpnmb expression in NBH and prions-infected brain slices at 30 wpi and terminal stages from a further biological replicate. Color intensity indicates expression levels, with cooler colors (blue) representing lower and warmer colors (yellow) higher expression. Scale bars, 0.5 mm. **b)** Densitometric quantification of Western Blots shown in Figure 2b. Data are presented as mean ± s.d.; statistical significance was assessed using two-sided unpaired Welch’s t-tests. **c)** Immunohistochemistry of brain sections stained for Iba-1 (microglia marker) and SAF84 (PrP^Sc^ marker) from control (NBH) and prion-infected mice at 30 wpi and terminal stages. Iba-1 intensity shows microglial presence and activation, while SAF84 highlights prion accumulation. Scale bars represent 0.5 mm.


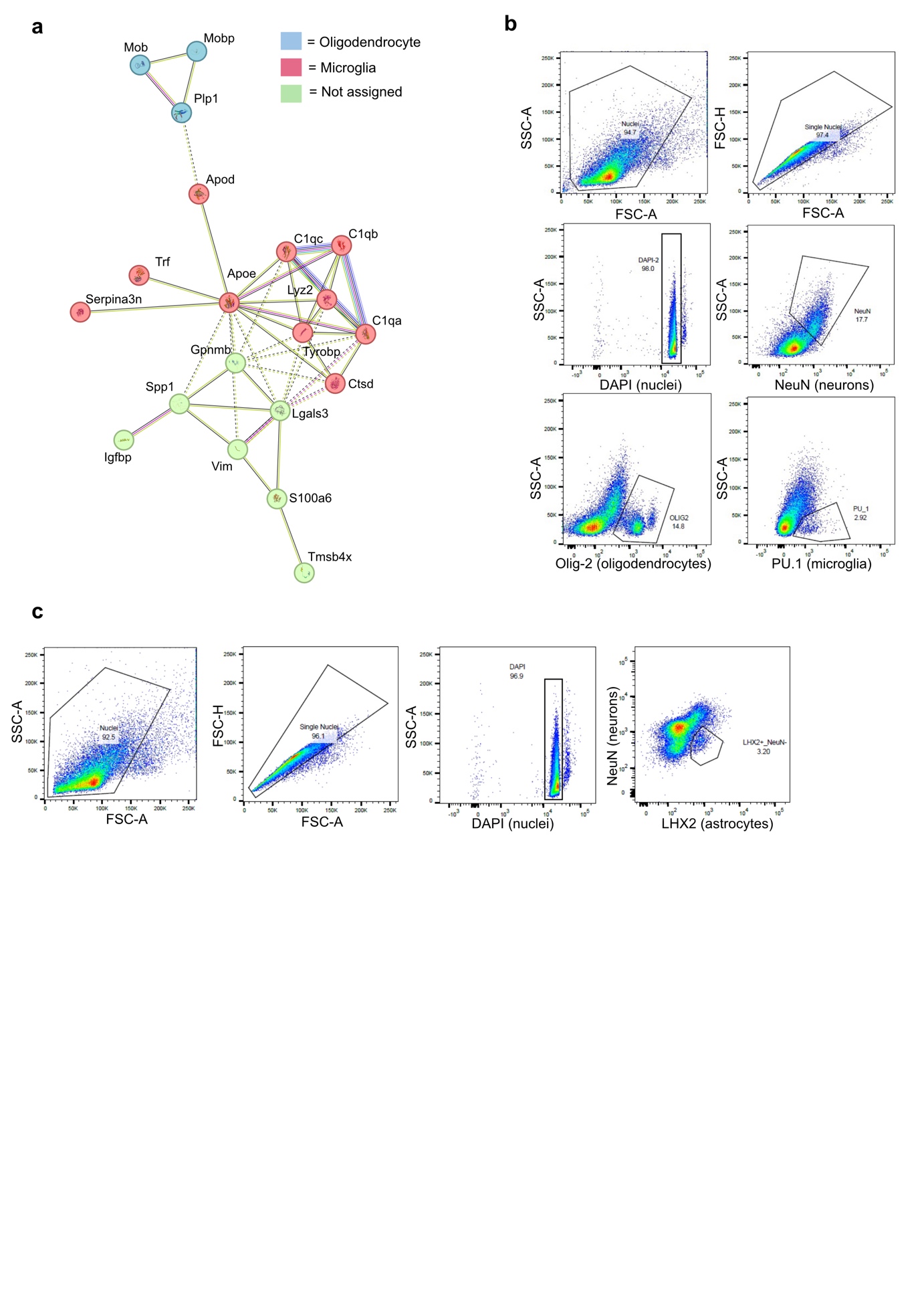


**Supplementary Figure 7.** **Gene network analysis and flow cytometry characterization assign Gpnmb^+^ profile to microglia.** **a)** STRING network of genes associated with the Gpnmb^+^ cell-type identity. Nodes represent genes, and edges indicate interactions. Node colors indicate different cell-type assignments: oligodendrocyte (light blue), microglial (red), or unassigned (green). **b)** Flow cytometry gating of nuclei from NBH and prion-infected brains Plots show selection of nuclei (FSC-A vs. SSC-A), singlets (FSC-H vs. FSC-A), and DAPI⁺ nuclei (Vio 450 vs. SSC-A), followed by gating for NeuN⁺ (neuronal), Olig2⁺ (oligodendrocyte), and PU.1⁺ (microglial) nuclei. **c)** Flow cytometry of astrocyte nuclei. Plots show selection of nuclei, singlets, DAPI⁺ nuclei, and final gating for NeuN⁻ Lhx2⁺ astrocyte nuclei.


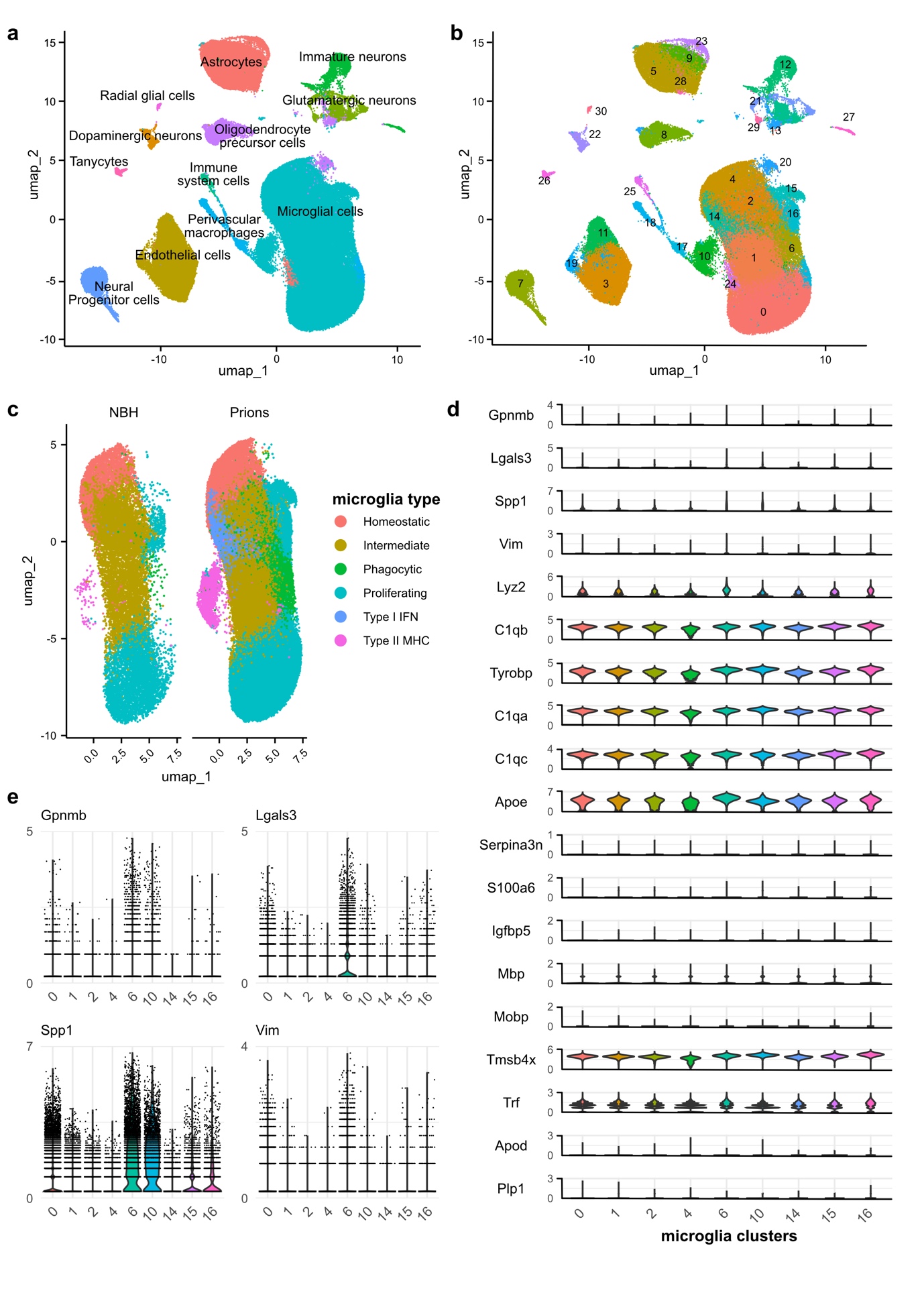


**Supplementary Figure 8**. **Single-cell RNA-seq analysis of microglia in prion disease.** Reanalysis of single-cell RNA sequencing data from terminally ill prion-infected mice. **a)** UMAP plots showing annotation of major CNS cell types. **b)** UMAP plots showing clustering of CNS cell types into distinct subtypes. **c)** UMAP of microglia sub-clusters in NBH and prion-infected conditions. **d)** Violin plots displaying the expression of 19 genes associated to the Gpnmb+ profile across microglial clusters. **e)** Violin plots with overlaid single-cell data points showing expression of *Gpnmb*, *Lgals3*, *Spp1* and *Vim* across microglial clusters.


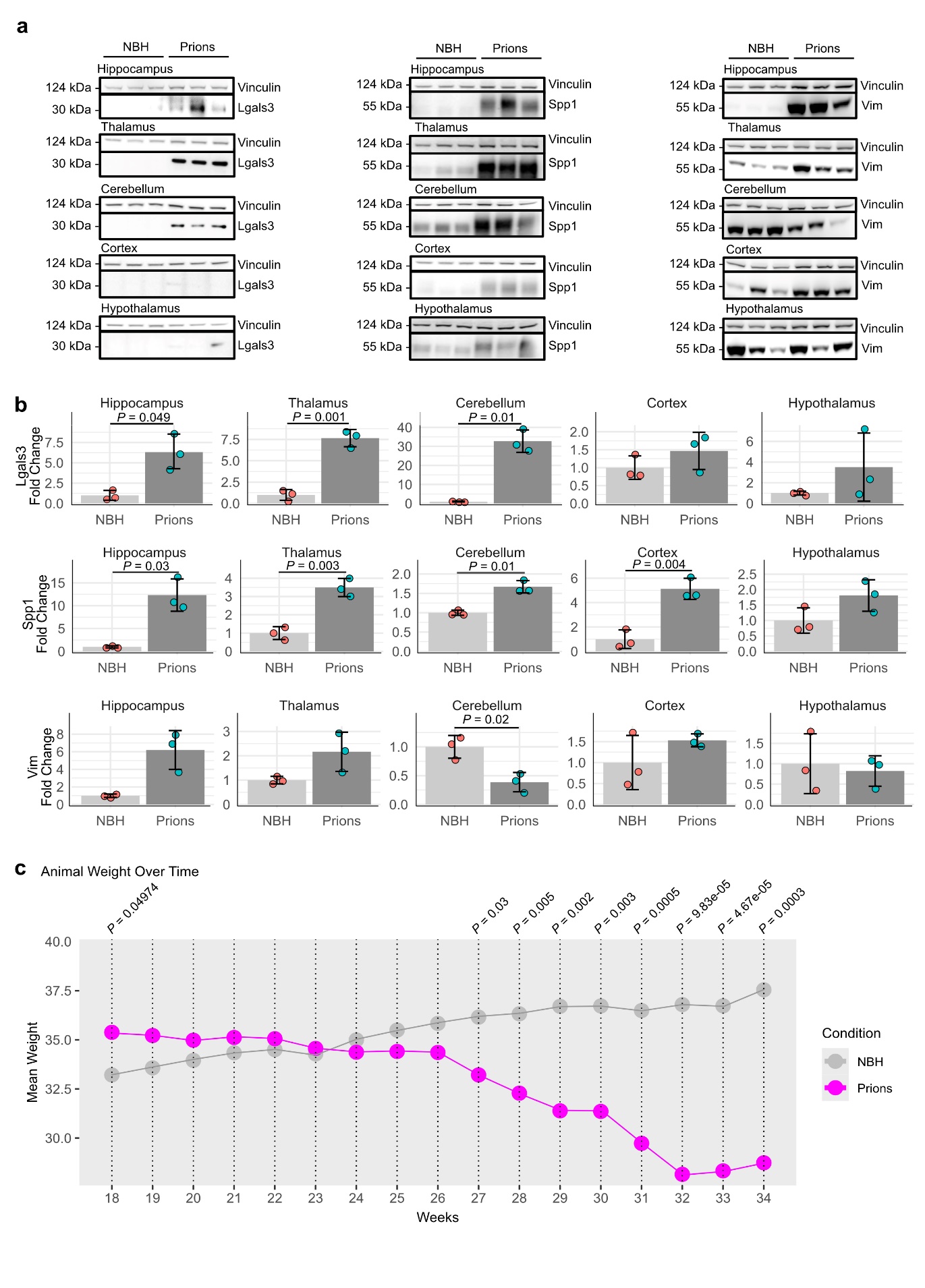
**Supplementary Figure 9.** **Expression of proteins associated to Gpnmb+ profile in different brain region and body weight changes in mice during prion progression.** **a)** Western blot showing Lgals3, Spp1, and Vim across different brain regions in control (NBH) and prion-infected mice at the terminal stage; vinculin serves as loading control. **b)** Densitometric quantification of blots from panel a). Data are presented as mean ± s.d., and statistical significance was assessed using two-sided unpaired Welch’s t-tests. **c)** Line chart showing mean body weight over time in NBH (grey) and prion-infected (magenta) mice (n = 10) assessed by weeks post-inoculation (x-axis). Two-sided Welch’s t-tests were performed at each time point.


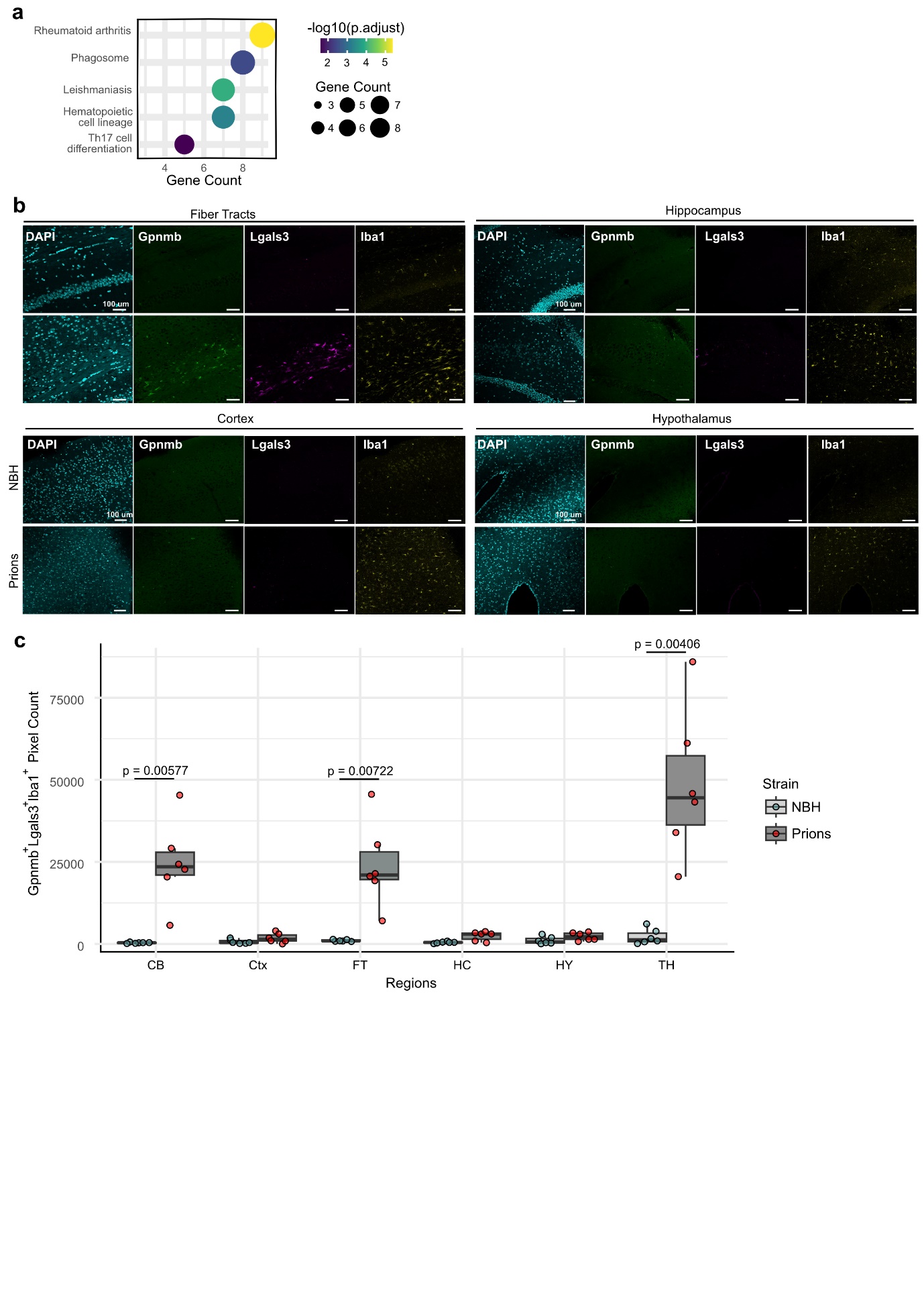


**Supplementary Figure 10. KEGG enrichment of Gpnmb+ microglia and regional Gpnmb and Lgals3 expression in microglia. a)**KEGG enrichment of upregulated genes in *Gpnmb*⁺/phagocytic Cluster 6⁺ versus *Gpnmb*⁻/phagocytic Cluster 6⁺ microglia from scRNA-seq data. Color indicates adjusted P-values (yellow = most significant), size indicates gene count. Statistical significance was assessed using Fisher’s exact test with Benjamini–Hochberg correction. **b)** Immunofluorescence targeting DAPI (cyan), Gpnmb (green), Lgals3 (magenta) and Iba1 (yellow) on terminally ill prion infected mice. Two ROI of different brain regions are shown. Scale bars represent 100 µm. **c)** Boxplot comparing the positive pixel count across different conditions. Each box represents the distribution of positive pixels for a given condition, with custom colors distinguishing the groups. Dots for each regional condition (CB = cerebellum, Ctx = cortex, FT = fiber tracts, HC = hippocampus, HY = hypothalamus, TH = thalamus) represent two technical replicates from three biological replicates (n = 3). The central line in each box indicates the median, box represents the interquartile range (25th–75th percentiles), and whiskers extend to the most extreme values within 1.5x the interquartile range. Outliers are shown as individual points. Pairwise comparison for each brain region was performed using two-sided Wilcoxon rank-sum tests.


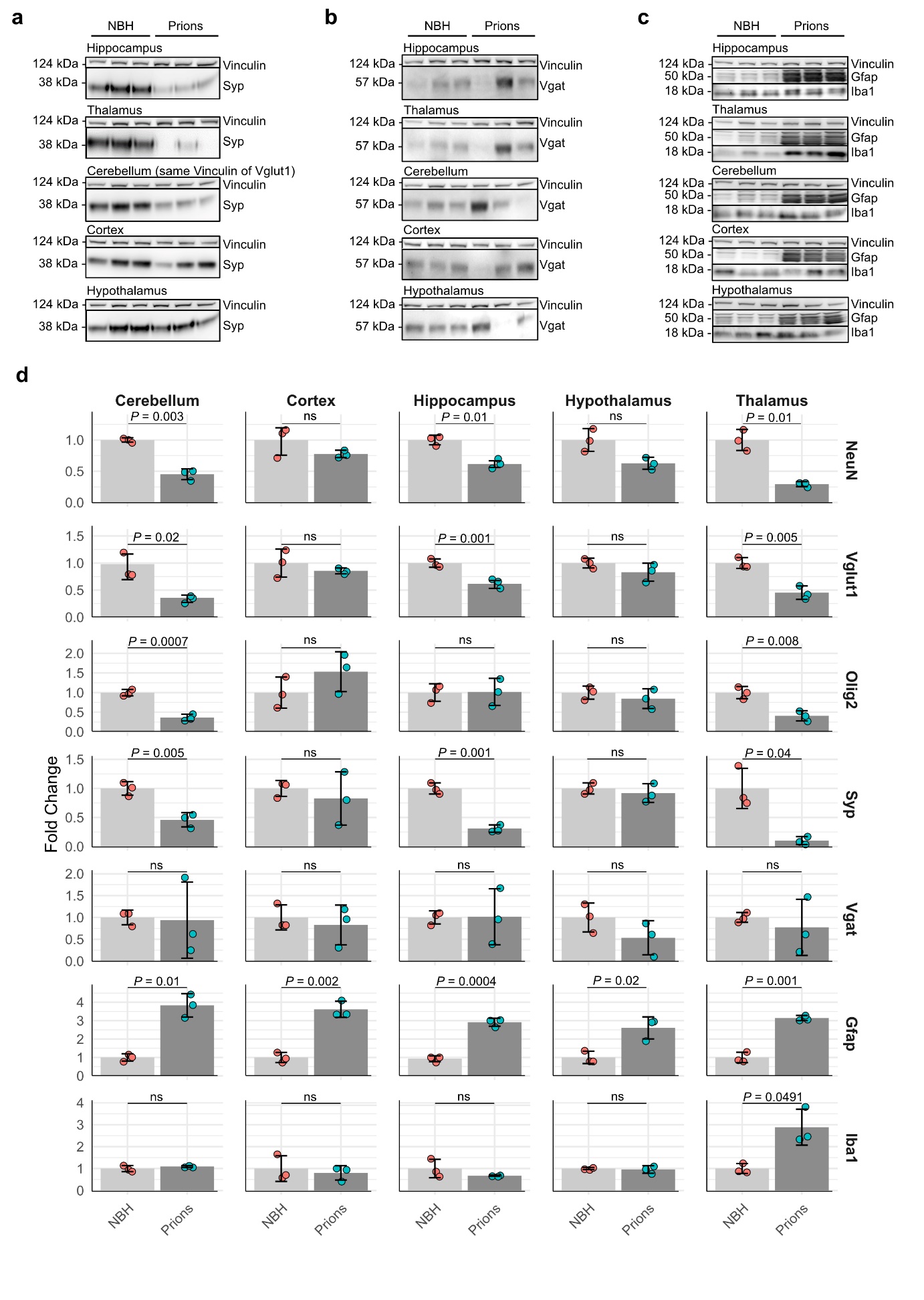
**Supplementary Figure 11**. **Neuronal and glial marker expression across brain regions in prion disease.** Western blot showing **a)** Syp, **b)** Vgat, **c)** Gfap and Iba1 protein levels across brain regions in control (NBH) and prion-infected samples assessed at the terminal stage; Vinculin serves as loading control. **d)** Densitometric analysis of the Western blots in Figure 5d and Supplementary Figure 11a–c, showing fold changes between NBH and prion-infected samples. Data are presented as mean ± s.d.; statistical significance was assessed using two-sided Welch’s t-tests.


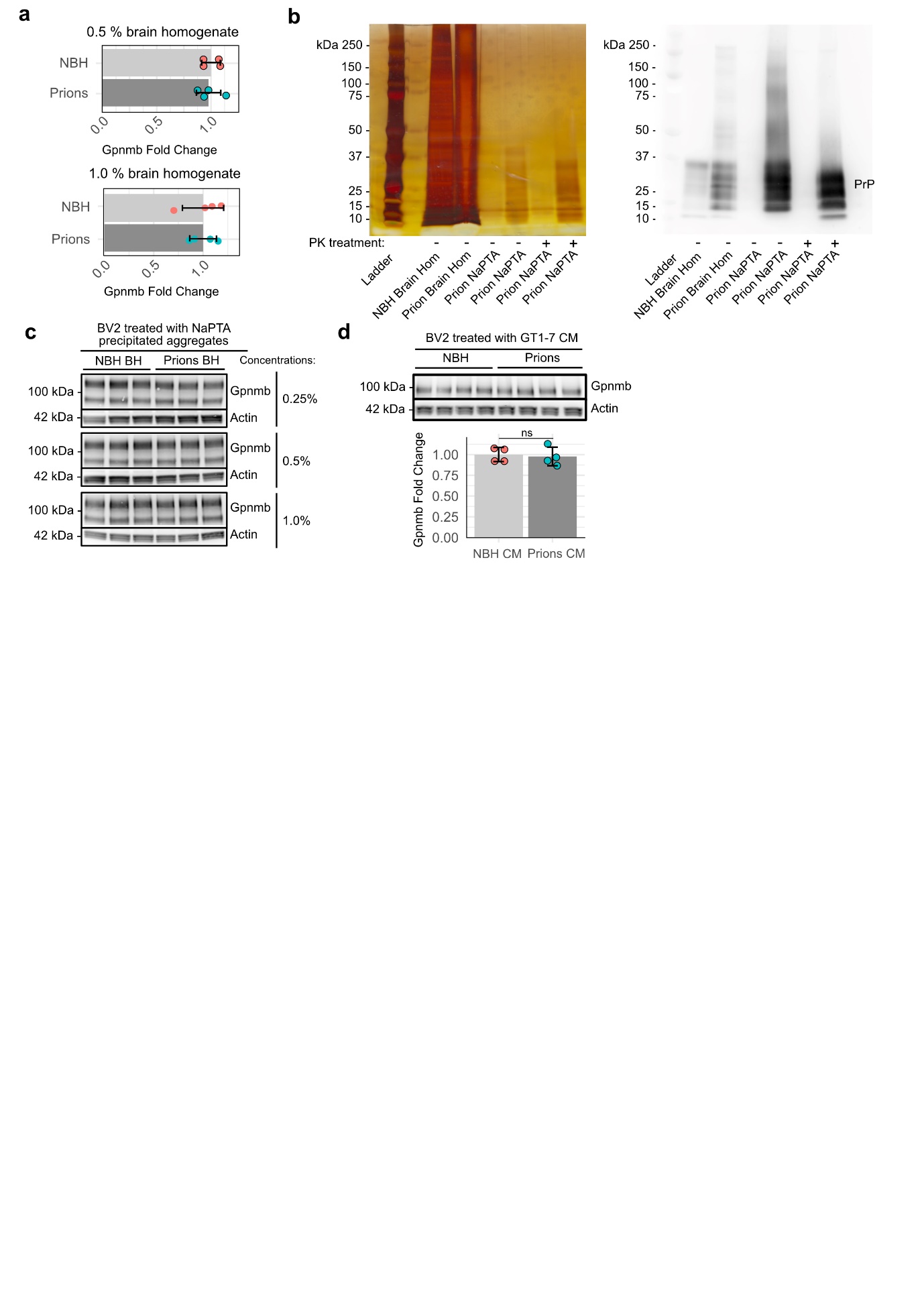


**Supplementary Figure 12.** **Gpnmb expression in BV2 cells following prion exposure. a)** qPCR of *Gpnmb* expression in BV2 cells treated with 0.5% and 1.0% brain homogenates from control (NBH) and RML6 prion-infected mice. **b)** Left, uncut silver-stained 4-12% SDS-PAGE gel showing NBH prion-infected brain homogenate alongside NaPTA-purified fractions. Right, corresponding uncut western blot probed with anti- PrP antibody (POM1). The "+" and "–" indicate the presence or absence of proteinase K (PK) treatment. **c)** Western blots showing Gpnmb expression in BV2 cells treated with NaPTA isolated aggregates from both NBH and prion-containing brain homogenates; Actin serves as loading control. Aggregates corresponding to 0.25%, 0.5% and 1% (w/v) brain homogenate were used as treatment. **d)** Western blot and densitometric quantification of Gpnmb in BV2 cells treated for 48 hours with conditioned media (CM) from prion-propagating GT1-7 cells. Data in **a** and **d** are presented as mean ± s.d., and statistical significance was assessed using two-sided Welch’s t-tests.


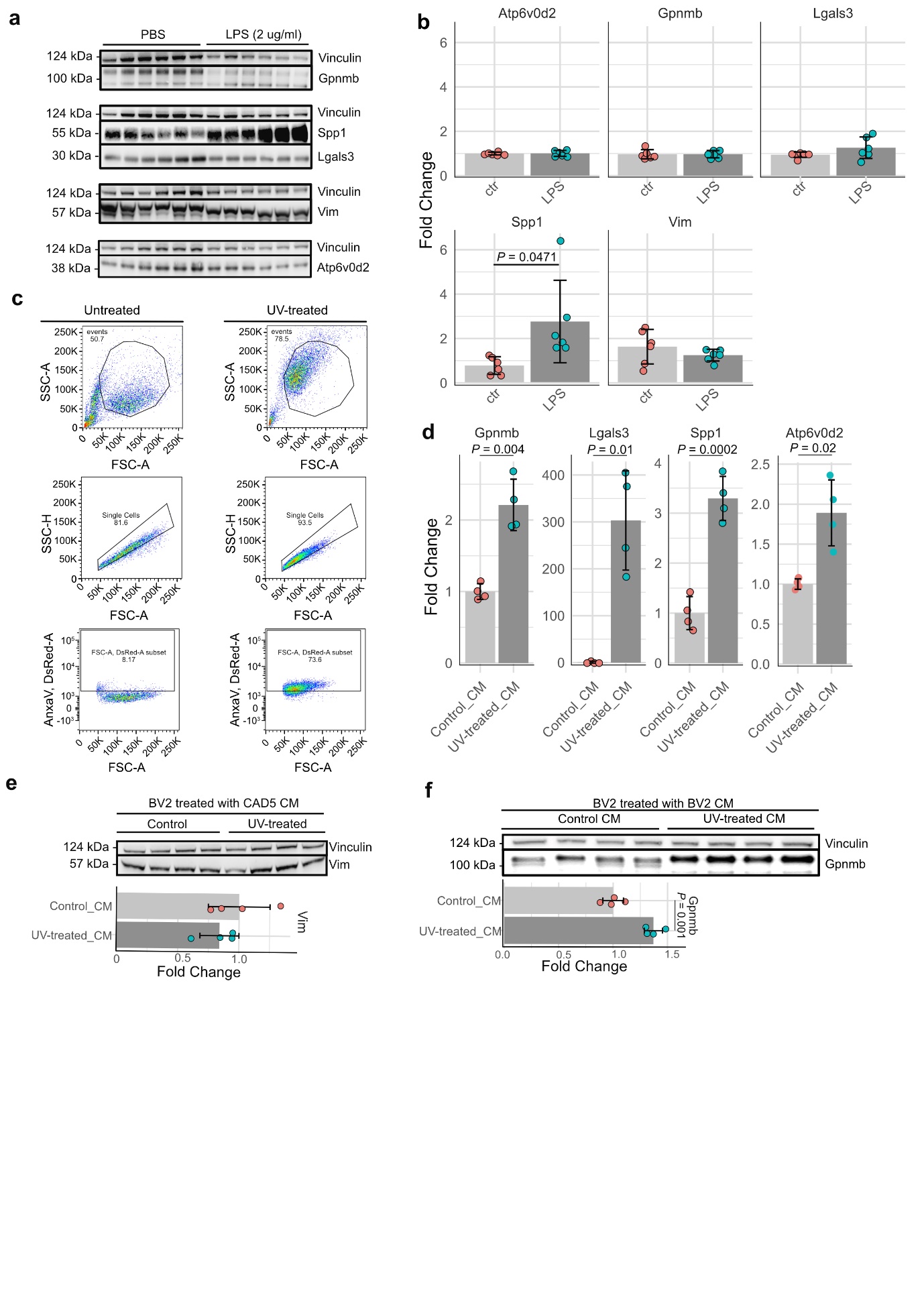


**Supplementary Figure 13. Gpnmb expression in BV2 responses upon LPS and apoptotic stimuli. a)** Western blot of BV2 cells treated with PBS or LPS (2 μg/ml) for 24 hours, probing Gpnmb, Spp1, Lgals3, Vim, Atp6v0d2; Vinculin serves as loading control. **b)** Densitometric quantification of panel a, showing fold changes in protein expression (PBS vs. LPS). **c)** Flow cytometry analysis of Annexin V AnxaV) in UV-treated and untreated CAD5 cells confirming apoptosis. **d)** Densitometric quantification of Western blot from Figure 6a in BV2 cells cultured with conditioned media (CM) from UV-treated and untreated (control) CAD5 cells. **e)** Western blot and densitometric quantification of Vim in BV2 cells treated with CM from UV-treated and untreated CAD5 cells, normalized to Vinculin. **f)** Western blot and densitometric quantification of Gpnmb in BV2 cells treated with CM from UV-treated and untreated (control) BV2 cells, normalized to Vinculin. Data in **b, d, e** and **f** are presented as mean ± s.d., and statistical significance was assessed using two-sided Welch’s t-tests.


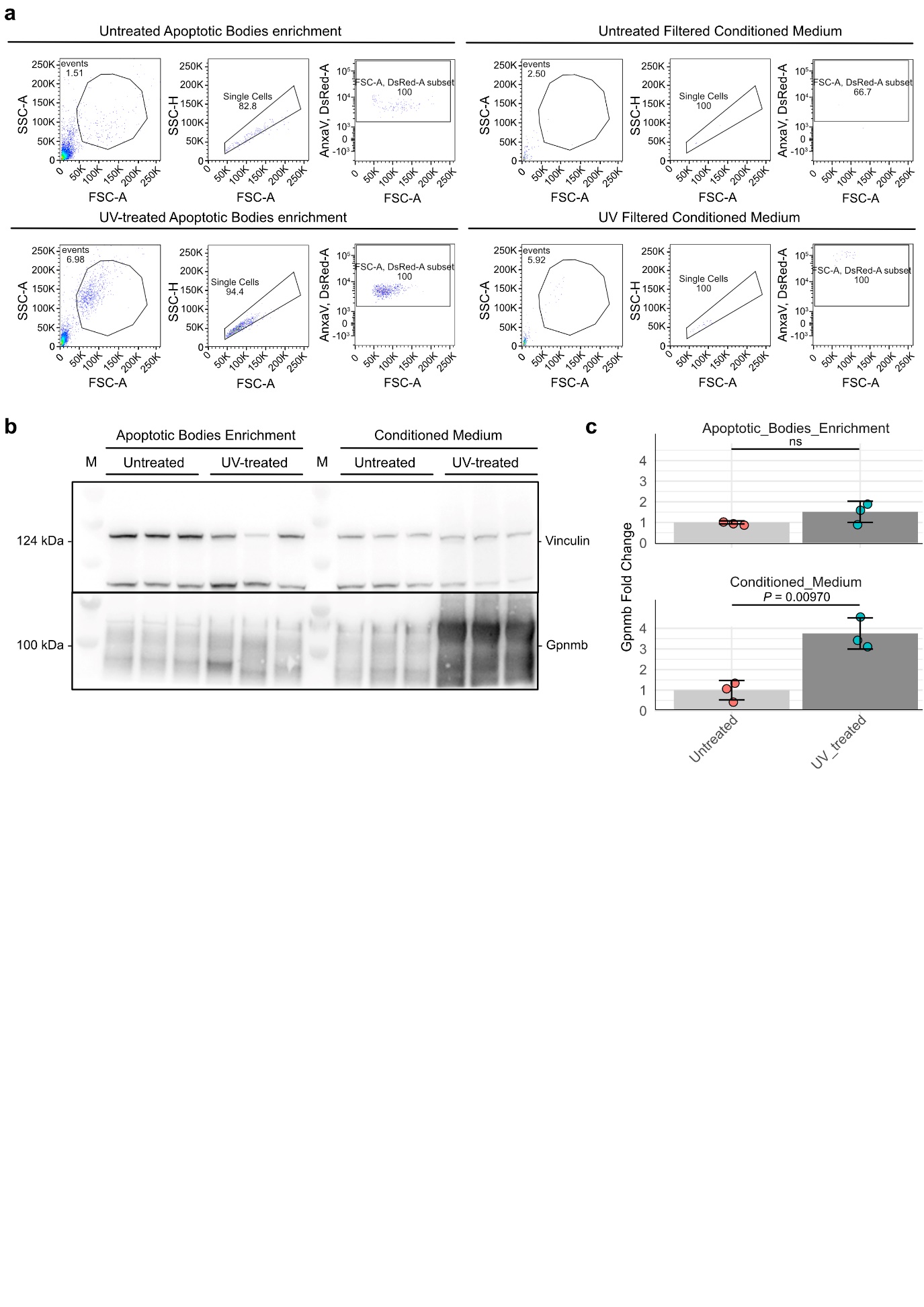


**Supplementary Figure 14. Gpnmb expression in BV2 cells upon treatment with apoptotic bodies enriched and depleted conditioned medium. a)** Flow cytometry analysis of apoptotic bodies and filtered conditioned medium from untreated and UV-treated CAD5 cells. Representative plots show selection of particles (FSC-A vs SSC-A), singlets (FSC-H vs FSC-A), and gating of apoptotic bodies (Annexin V⁺ events). **b)** Uncut western blot of Gpnmb in BV2 cells treated with apoptotic bodies enriched and depleted (filtered) conditioned medium from UV-treated and untreated CAD5 cells; Vinculin serves as a loading control. **c)** Densitometric quantification of Gpnmb from panel b, shown as fold change relative to untreated samples. Data are presented as mean ± s.d., and statistical significance was assessed using two-sided Welch’s t-tests.
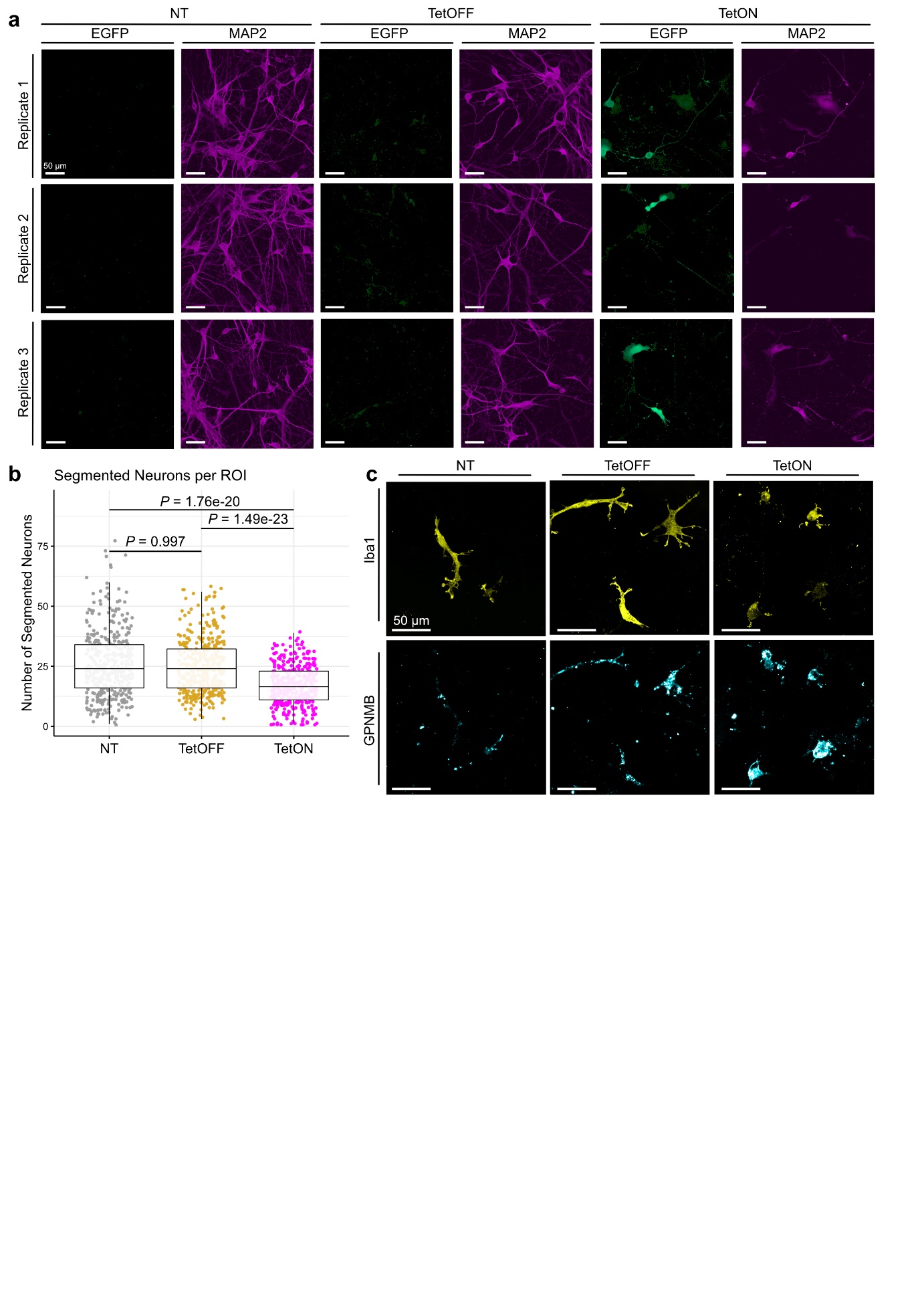


**Supplementary Figure 15. Neuronal cell death and microglial Gpnmb expression in human iPSC-derived co-culture. a)** Representative immunofluorescence images (40x) from three biological replicates of human iNet–microglia co-cultures. EGFP (green) and MAP2 (magenta) channels are shown to visualize neuronal structure and health under NT (non-treated), TetOFF (doxycycline withdrawn), and TetON (doxycycline administered) conditions. Scale bars represent 50 µm. **b)** Quantification of segmented neurons per region of interest (ROI) across all conditions (related to Figure 6b). Each point represents MAP2-derived ROIs from two biological experiments. Box plots show the median, interquartile range (25th–75th percentiles), and whiskers extend to the most extreme values within 1.5x the interquartile range. Pairwise comparisons were performed using two-sided Wilcoxon rank-sum tests with Benjamini-Hochberg correction. **c)** Representative confocal images (63x) showing Iba1 (yellow) and GPNMB (cyan) immunostaining in the NT, TetOFF, and TetON conditions. Scale bars represent 50 µm.


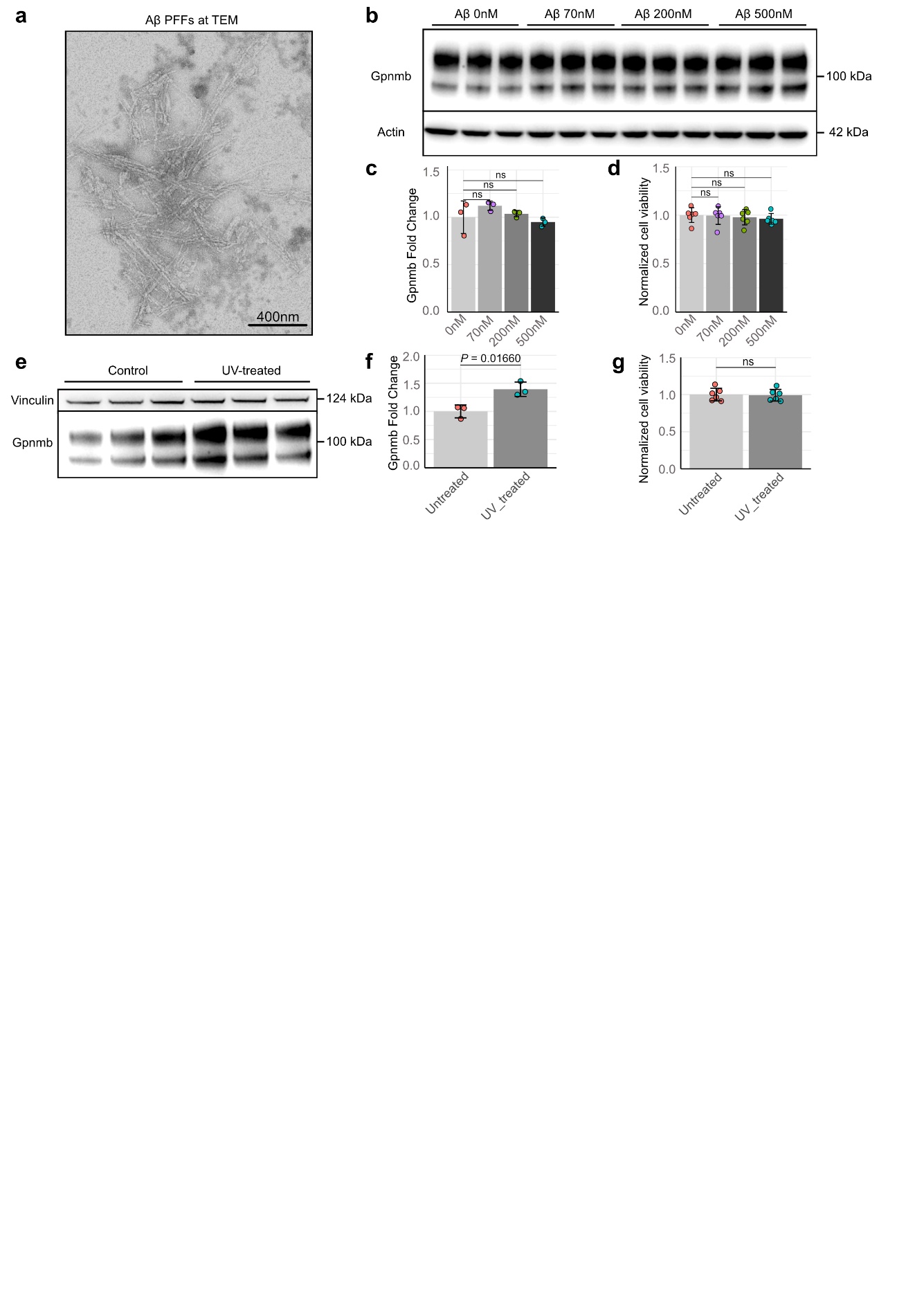


**Supplementary Figure 16. Gpnmb expression in BV2 cells upon Aβ fibrils treatment. a)** Transmission electron microscopy (TEM) image of generated amyloid-β (Aβ) pre-formed fibrils (PFFs). Scale bars represent 400 nm. **b)** Western blot of Gpnmb expression in BV2 cells treated with increasing concentrations of Aβ fibrils (0–500 nM); Actin serves as a loading control. **c)** Densitometric quantification of Gpnmb from panel b, normalized to Actin. **d)** Cell viability assessed by CellTiter-Glo following treatment with increasing concentrations of Aβ fibrils, expressed as fold change relative to the 0 nM condition. **e)** Western blot of Gpnmb in BV2 cells exposed to UV-treated and untreated (control) conditioned medium; Vinculin serves as a loading control. **f)** Densitometric quantification of Gpnmb from panel e, normalized to Vinculin. **g)** Cell viability assessed by CellTiter-Glo in BV2 cells exposed to UV-treated and untreated (control) conditioned medium, expressed as fold change relative to control. Data in **c, d, f** and **g** are presented as mean ± s.d.. For **c** and **d**, statistical significance was assessed using two-sided one-way ANOVA followed by Dunnett’s multiple comparison test; in **f** and **g** using two-sided Welch’s t-tests.


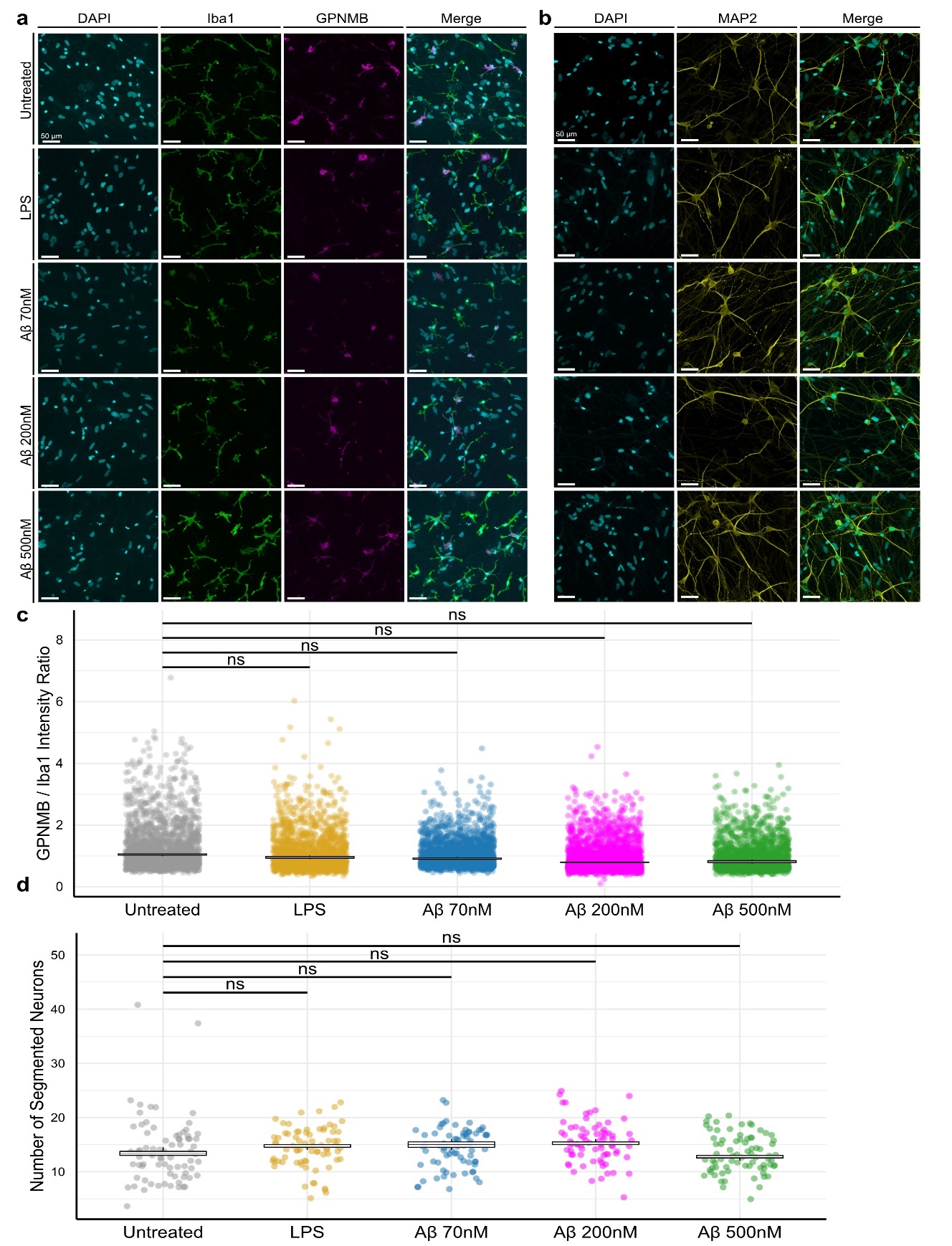


**Supplementary Figure 17. Microglial GPNMB expression and neuronal integrity upon LPS and Aβ treatment in human iPSC-derived co-culture.** **a)** Representative immunofluorescence images of cultured cells stained for DAPI (cyan), Iba1 (green), and GPNMB (magenta) under untreated condition, LPS stimulation, or Aβ treatment (70, 200 and 500 nM). Scale bars represent 50 µm. **b)** Immunofluorescence staining for DAPI (cyan) and MAP2 (yellow) to assess neuronal density and morphology across the same conditions. The same wells imaged in panel a) were re-stained for MAP2 and imaged at the same regions of interest. Minor positional shifts occurred due to remounting; DAPI was re-acquired to document imaging fields. Scale bars represent 50 µm. **c)** Quantification of **GPNMB/Iba1 intensity ratio** from panel a). Each dot represents an individual measurement box plots summarize medians from three biological replicates per condition (n = 3). **d)** Quantification of segmented neurons from panel b). Each dot represents one ROI; box plots summarize replicate-level medians (n = 3). Statistical analysis for panels **c)** and **d)** was performed using two-sided Wilcoxon rank-sum tests on replicate medians versus untreated controls, with Benjamini-Hochberg correction for multiple testing.


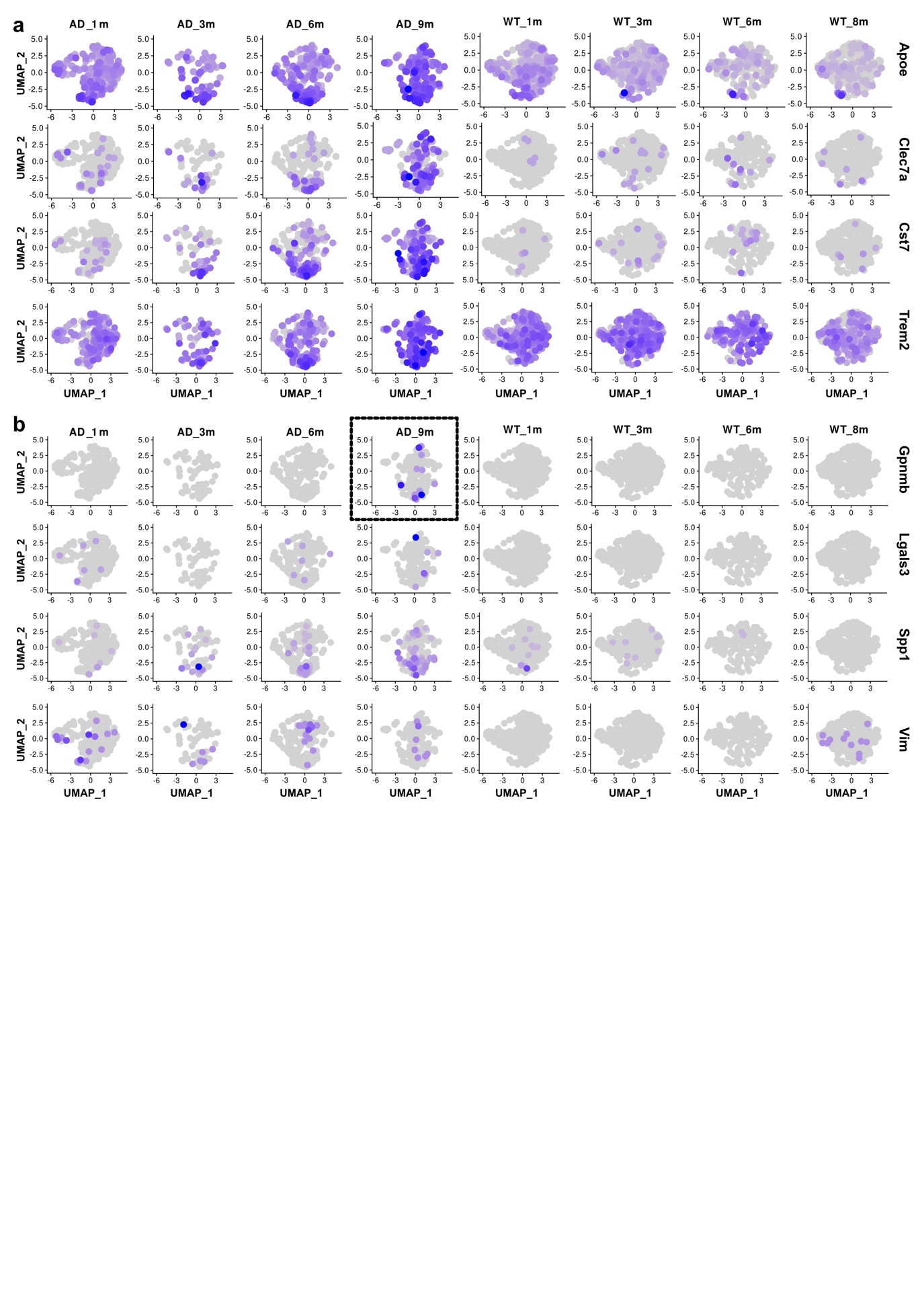


**Supplementary Figure 18**. **UMAP visualization microglia transcripts from Alzheimer’s disease (AD) model mice (5xFAD) and age-matched wild-type (WT) controls across multiple ages**. **a)** Microglial UMAPs colored by expression of canonical DAM and microglial markers (*Apoe*, *Clec7a*, *Cst7*, and *Trem2*) across disease and control conditions, illustrating the progressive activation of DAM-like transcriptional programs in AD but not WT microglia, and **b)** UMAPs showing expression of *Gpnmb*, *Lgals3*, *Spp1*, and *Vim* genes, with *Gpnmb* expression restricted to a small subset of microglia at late AD stages (boxed).


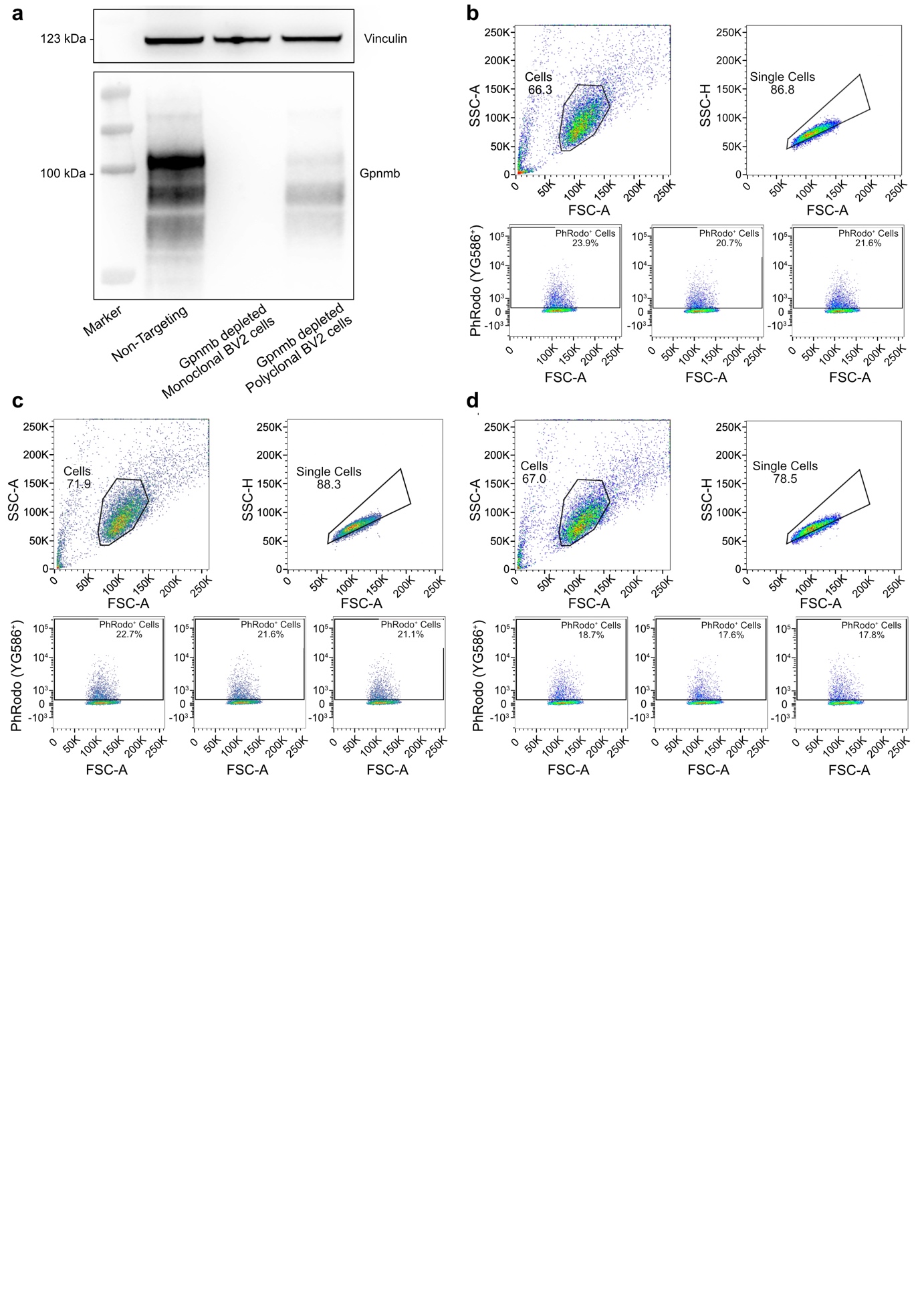


**Supplementary Figure 19. Gpnmb depletion and phagocytic activity in BV2 cells. a)** Western blot showing Gpnmb protein levels in non-targeting control, Gpnmb-depleted monoclonal (complete depletion) and polyclonal (partial depletion) BV2 cells; Vinculin serves as a loading control. **b–d) F**low cytometry gating strategy for phagocytic activity using pHrodo-labeled apoptotic CAD5 cells. Top rows: gating for cells (FSC-A vs SSC-A) and singlets (FSC-H vs FSC-A); bottom rows: gating for pHrodo⁺ cells from three independent experiments. **b)** non-targeting control, **c)** partially Gpnmb-depleted polyclonal, and **d)** fully Gpnmb-depleted monoclonal BV2 cells.


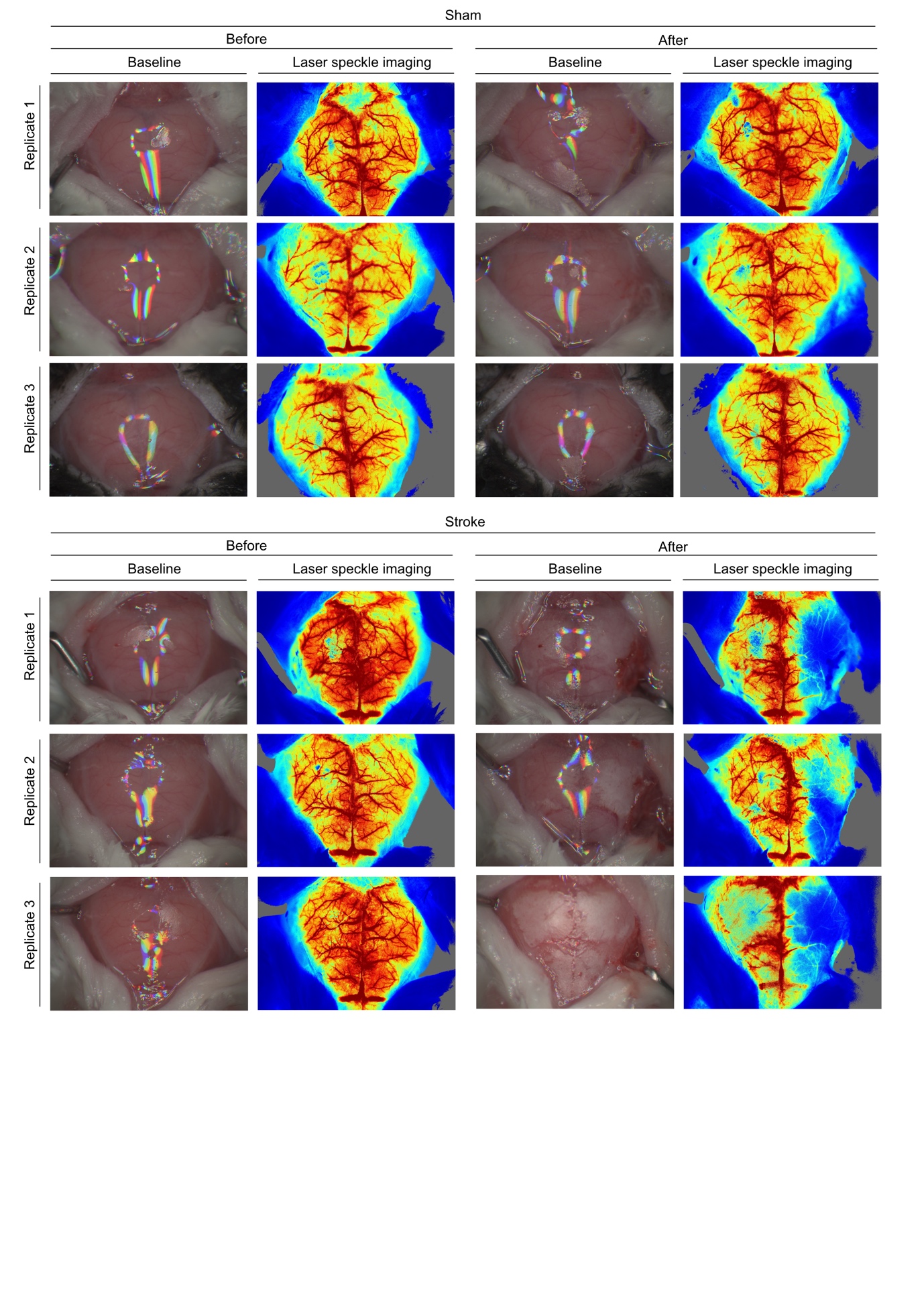


**Supplementary Figure 20**. **Cerebral blood flow before and after sham or stroke procedures.** Images of cranial windows from individual mice before and after sham or stroke procedures. Brightfield images show the exposed cortical surface, while pseudocolor heatmaps depict relative cerebral blood flow via laser speckle contrast imaging (warmer colors = higher perfusion, cooler colors = lower perfusion). Sham animals show stable perfusion patterns before and after the procedure, whereas stroke animals display a marked reduction in cortical blood flow after ischemia induction.


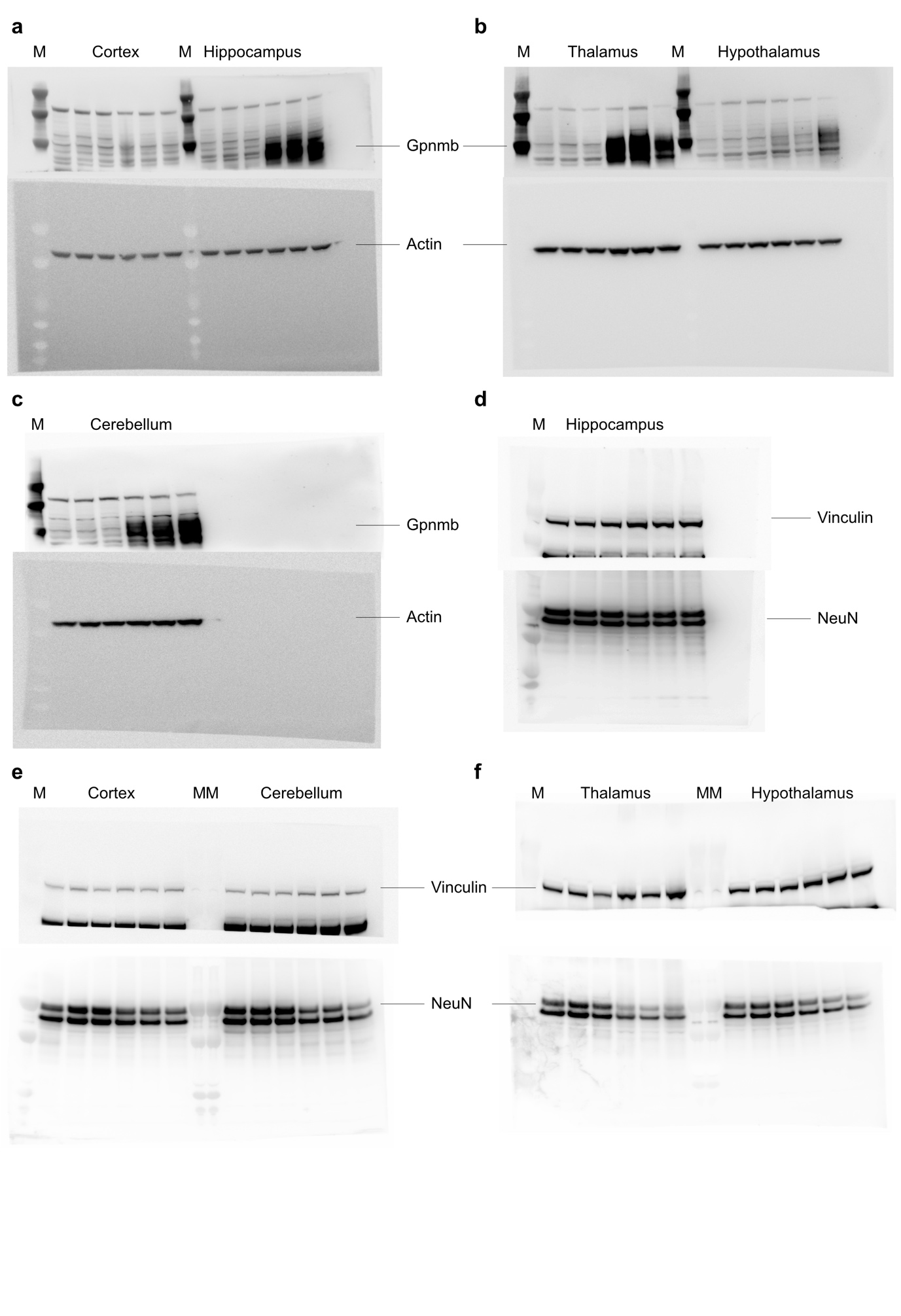


**Uncut and unadjusted Western blots Figure 2b and 5d.** **a–c)** Figure 2b showing Gpnmb and Actin in **a)** cortex and hippocampus, **b)** thalamus and hypothalamus and **c)** cerebellum. **d–e)** Figure 5d left panel showing Vinculin and NeuN in **d)** hippocampus, **e)** cortex and cerebellum and **f)** thalamus and hypothalamus. “M” symbol indicates the molecular weight marker. The membranes were cut into two sections and stained with the indicated antibodies.


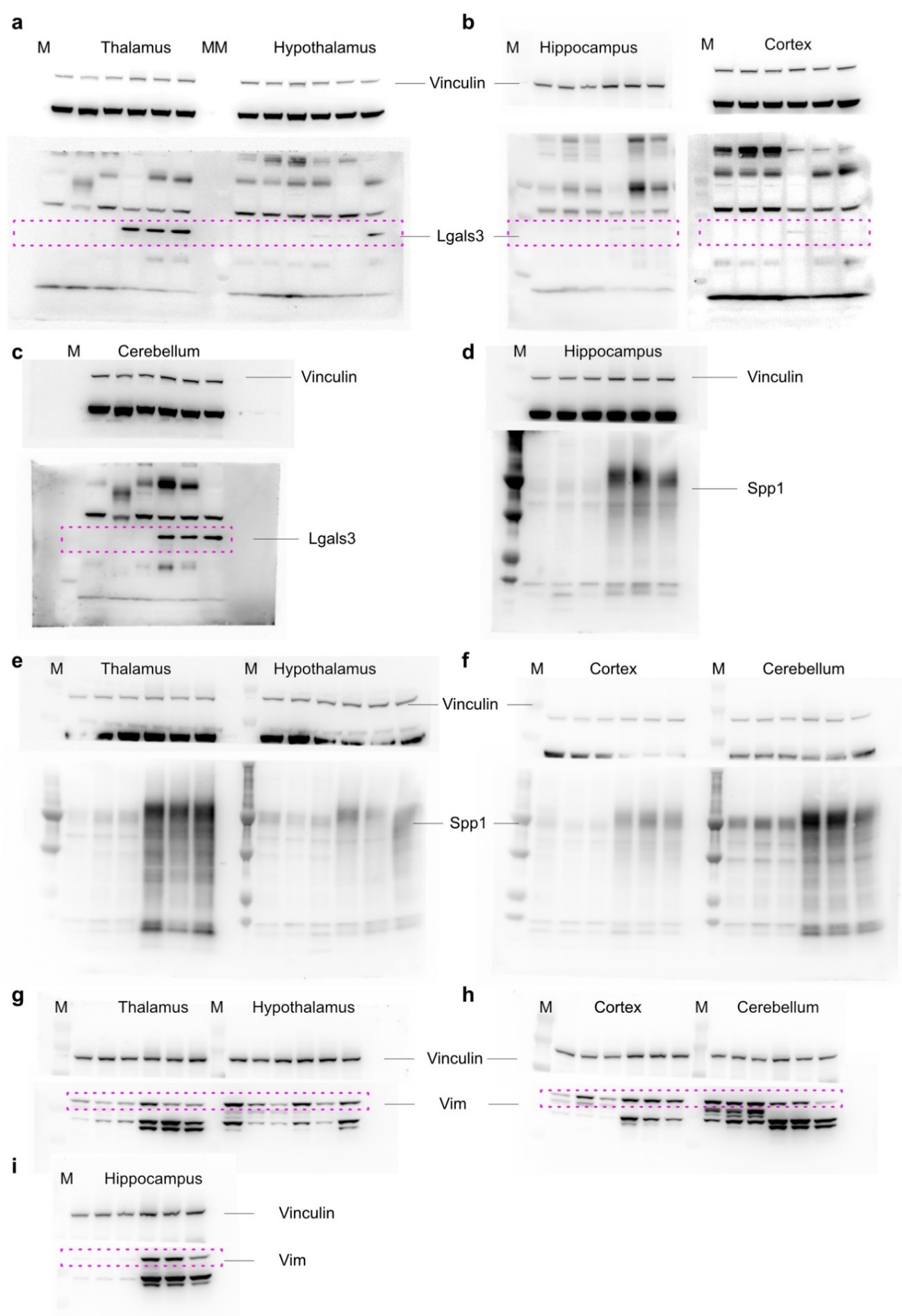


**Uncut and unadjusted Western blots Supplementary Figure 9a.** **a–c)** Figure 9a left panel showing Lgals3 and Vinculin in **a)** thalamus and hypothalamus, **b)** hippocampus and cortex and **c)** cerebellum. **d–f)** Figure 9a middle panel showing Spp1 and Vinculin in **d)** hippocampus, **e)** thalamus and hypothalamus and **f)** cortex and cerebellum. **g–i)** Figure 9a right panel showing Vim and Vinculin in **g)** thalamus and hypothalamus, **h**) cortex and cerebellum and **i)** hippocampus. Pink-dotted rectangles highlight bands corresponding to target-specific signal among other bands. “M” indicates the molecular weight marker. The membranes were cut into two sections and stained with the indicated antibodies.


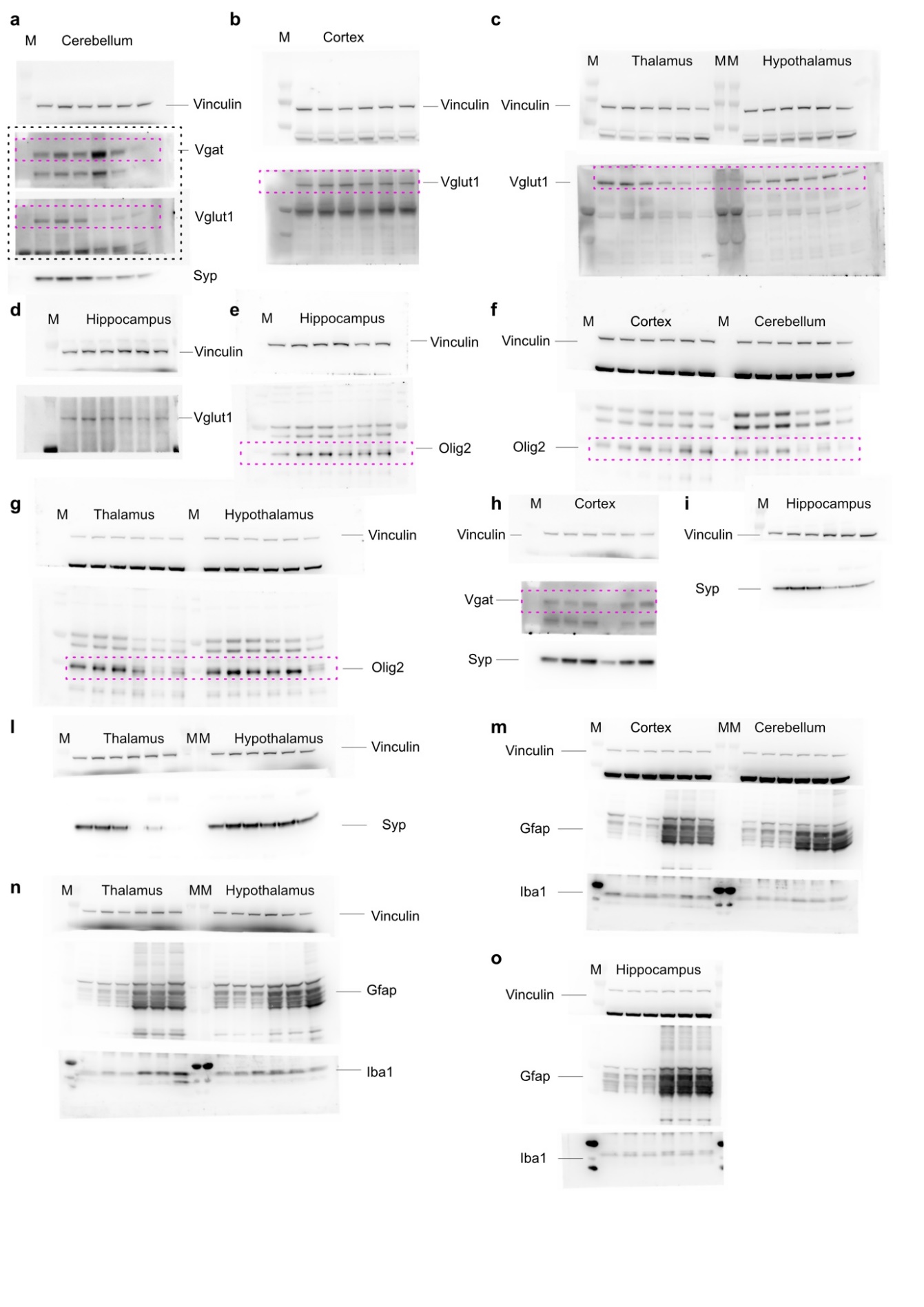


**Uncut and unadjusted Western blots Figure 5d and Supplementary Figure 11a–c.** **a)** Figure 5d middle panel and Supplementary Figure 11a–b: Western blot was divided into three sections and probed for Vinculin (top), Vgat and Vglut1 (middle), and Syp (lower) in the cerebellum. The black-dotted rectangle indicates the same blot section initially stained with Vglut1, then stripped and re-probed with Vgat. **b–d)** Figure 5d middle panel showing Vinculin and Vglut1 in **b)** cortex, **c)** thalamus and hypothalamus, and **d)** hippocampus. **e–f)** Figure 5d right panel showing Vinculin and Olig2 in **e)** hippocampus, **f)** cortex and cerebellum and **g)** thalamus and hypothalamus. **h)** Supplementary Figure 11a–b showing Vinculin, Vgat and Syp in cortex. **I–l)** Supplementary Figure 11a showing Vinculin and Syp in **i)** hippocampus, and **l)** thalamus and hypothalamus. **m–o)** Supplementary Figure 11c showing Vinculin, Gfap and Iba1 in **m)** cortex and cerebellum, **n)** thalamus and hypothalamus, and **o)** hippocampus. Pink-dotted rectangles highlight bands corresponding to target-specific signal among other bands. “M” indicates the molecular weight marker. The membranes were cut into two sections and stained with the indicated antibodies.


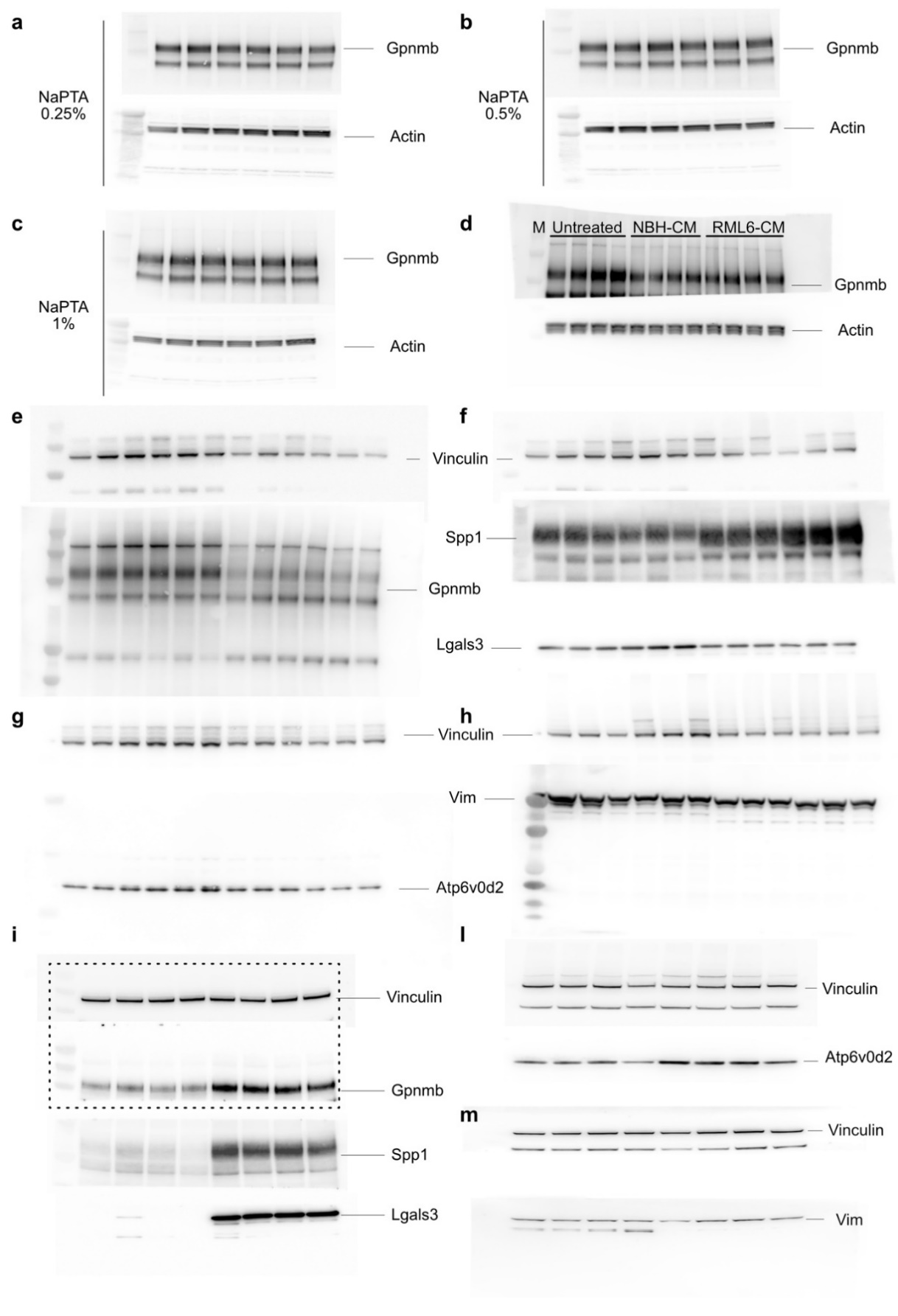


**Uncut and unadjusted Western blots Figure 6a, and Supplementary Figure 12c–d and 13a.** **a–c)** Supplementary Figure 12c showing Gpnmb and Actin in BV2 cells treated with **a)** 0.25%, **b)** 0.5% and **c)** 1% NaPTA-purified prions. **d)** Supplementary Figure 12d showing Gpnmb and Actin in untreated, NBH CM, and RML6 CM treated samples. Untreated samples were not included in the manuscript. **e–h)** Supplementary Figure 13a showing **e)** Gpnmb and Vinculin (membrane was stained for Gpnmb, stripped and stained for Vinculin), **f)** Spp1, Lgals3, and Vinculin (membrane was cut in three sections), **g)** Atp6v0d2 and Vinculin, and **h)** Vim and Vinculin. **i–m)** Figure 6a showing **i)** Gpnmb, Spp1, Lgals3 and Vinculin (membrane was cut into three sections; black-dotted rectangle highlights the same section, which was stained for Gpnmb, stripped and stained for Vinculin), **l)** Atp6v0d2 and Vinculin, and **m)** Vim and Vinculin. “M” indicates the molecular weight marker.


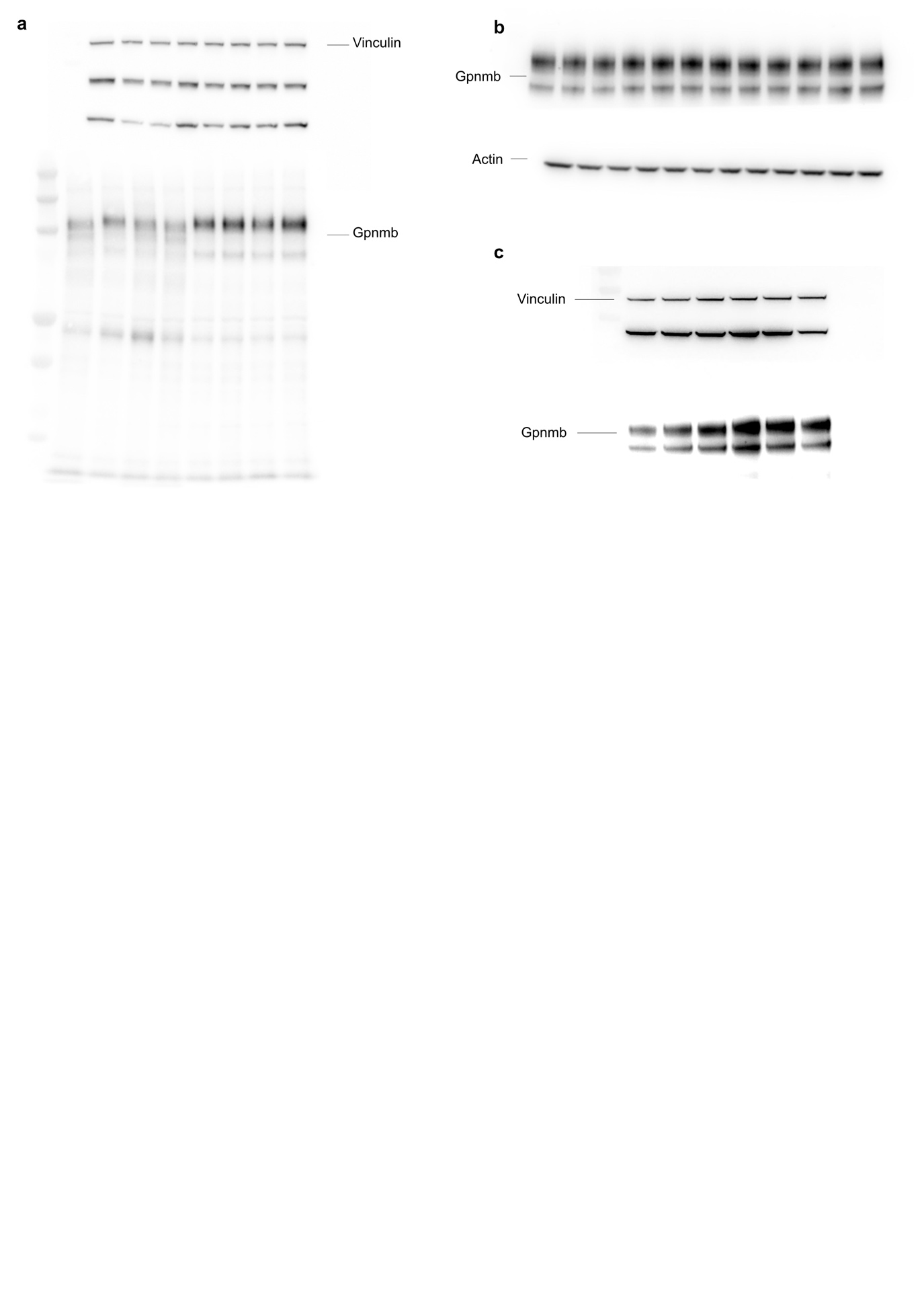


**Uncut and unadjusted Western blots Supplementary Figure 13f and 16b–c.** **a)** Supplementary Figure 13f showing Gpnmb and Vinculin; membrane was stained for Gpnmb, then stripped, further cut and stained for Vinculin. **b)** Supplementary Figure 16b showing Gpnmb and Actin; the membrane was divided into two sections and stained. **c)** Supplementary Figure 16c showing Gpnmb and Vinculin; membrane was stained for Gpnmb, then stripped and stained for Vinculin.
