# Supplementary material for: Gpnmb defines a phagocytic state of microglia linked to cell death in prion disease mouse model
Source: Nat Commun. 2026 May 12;17:6138. doi: 10.1038/s41467-026-73003-5 (PMC13365222; doi:10.1038/s41467-026-73003-5)
Supplement: Supplementary file 2 — Description of Additional Supplementary Files [file 41467_2026_73003_MOESM2_ESM.pdf]

Description of Additional Supplementary Files

Supplementary Data 1. Differentially expressed gene (DEG) analysis between prion-infected brain regions and NBH controls of 30 week post inoculation timepoint. Differential expression was assessed using DESeq2 (negative binomial distribution model and two-sided Wald tests), p-values were adjusted using the Benjamini–Hochberg method to control the false discovery rate (FDR). Genes with adjusted $p < 0.05$ were considered significant.

Supplementary Data 2. Differentially expressed gene (DEG) analysis between prion-infected brain regions and NBH controls of terminal-stage timepoint. Differential expression was assessed using DESeq2 (negative binomial distribution model and two-sided Wald tests), p-values were adjusted using the Benjamini–Hochberg method to control the false discovery rate (FDR). Genes with adjusted $p < 0.05$ were considered significant.

Supplementary Data 3. Shared modulated genes between timepoints and across regions.

Supplementary Data 4. STdeconvolve output results showing enriched genes belogning to each identified cell-type (Topic) of 30 week post inoculation timepoint.

Supplementary Data 5. STdeconvolve output results showing enriched genes belogning to each identified cell-type (Topic) of terminal-stage timepoint.

Supplementary Data 6. Patients' information relative to Figure 2c.

Supplementary Data 7. Patients' information relative to Figure 2e.

Supplementary Data 8. GO terms of the 19 genes shared between the two *Gpnmb*+, STdeconvolved-derived topics identified in two distinct prion-infected biological replicates. Fisher's exact test was used to identify enriched terms, with p-values adjusted using the Benjamini–Hochberg method to control FDR.

Supplementary Data 9. DEGs obtained comparing *Gpnmb*$^{+}$ vs *Gpnmb*$^{-}$ microglia within phagocytic Cluster 6. Differential expression was assessed using DESeq2 (negative binomial distribution model and two-sided Wald tests), p-values were adjusted using the Benjamini–Hochberg method to control the false discovery rate (FDR). Genes with adjusted $p < 0.05$ were considered significant.
