# Supplementary material for: Gpnmb defines a phagocytic state of microglia linked to cell death in prion disease mouse model
Source: Nat Commun. 2026 May 12;17:6138. doi: 10.1038/s41467-026-73003-5 (PMC13365222; doi:10.1038/s41467-026-73003-5)
Supplement: Supplementary file 12 — Reporting summary [file 41467_2026_73003_MOESM12_ESM.pdf]

nature portfolio

Corresponding author(s): Dr. Elena De Cecco & Prof. Adriano Aguzzi

Last updated by author(s): 2026.04.10

Reporting Summary

Nature Portfolio wishes to improve the reproducibility of the work that we publish. This form provides structure for consistency and transparency in reporting. For further information on Nature Portfolio policies, see our Editorial Policies and the Editorial Policy Checklist.

Statistics

For all statistical analyses, confirm that the following items are present in the figure legend, table legend, main text, or Methods section.

n/a	Confirmed	
☐	☒	The exact sample size (*n*) for each experimental group/condition, given as a discrete number and unit of measurement
☐	☒	A statement on whether measurements were taken from distinct samples or whether the same sample was measured repeatedly
☐	☒	The statistical test(s) used AND whether they are one- or two-sided<br>*Only common tests should be described solely by name; describe more complex techniques in the Methods section.*
☐	☒	A description of all covariates tested
☐	☒	A description of any assumptions or corrections, such as tests of normality and adjustment for multiple comparisons
☐	☒	A full description of the statistical parameters including central tendency (e.g. means) or other basic estimates (e.g. regression coefficient) AND variation (e.g. standard deviation) or associated estimates of uncertainty (e.g. confidence intervals)
☐	☒	For null hypothesis testing, the test statistic (e.g. *F*, *t*, *r*) with confidence intervals, effect sizes, degrees of freedom and *P* value noted<br>*Give P values as exact values whenever suitable.*
☒	☐	For Bayesian analysis, information on the choice of priors and Markov chain Monte Carlo settings
☐	☒	For hierarchical and complex designs, identification of the appropriate level for tests and full reporting of outcomes
☒	☐	Estimates of effect sizes (e.g. Cohen's *d*, Pearson's *r*), indicating how they were calculated

Our web collection on statistics for biologists contains articles on many of the points above.

Software and code

Policy information about availability of computer code

Data collection	Data were collected using standard manufacturer-provided acquisition software associated with the respective instruments (e.g. Illumina NovaSeq 6000 sequencing control software; Leica Stellaris confocal microscope acquisition software; Hamamatsu NanoZoomer slide scanner software; Molecular Devices ImageXpress acquisition software; BD flow cytometer acquisition software). No custom software or code was used for data collection.
Data analysis	Sequencing data were processed using 10x Genomics Space Ranger (v2.0.0). Downstream spatial transcriptomics analyses were performed in R using Seurat (v4.0.5) and differential expression testing with MAST (v1.12.0). Single-cell RNA-seq preprocessing included doublet detection with scrublet and ambient RNA removal with decontX, followed by integration with Harmony. Image processing and quantification were performed using Fiji/ImageJ (ImageJ2) with custom macros/scripts. Flow cytometry data were analyzed using FlowJo (v10). Additional statistical analyses and visualizations used standard R/Python packages and GraphPad Prism, and differential expression analyses were performed using DESeq2 where indicated. Custom scripts (shell/R) used for data organization and analysis are publicly available (see Data and Code Availability). Custom code used to reproduce the analyses and figures presented in this manuscript is available at https://github.com/dcare91/ST_prions (https://doi.org/10.5281/zenodo.18983911). STdeconvolve used to deconvolve each ST spot is available at. https://github.com/JEFworks-Lab/STdeconvolve.git.

For manuscripts utilizing custom algorithms or software that are central to the research but not yet described in published literature, software must be made available to editors and reviewers. We strongly encourage code deposition in a community repository (e.g. GitHub). See the Nature Portfolio guidelines for submitting code & software for further information.

Data

Policy information about availability of data

All manuscripts must include a data availability statement. This statement should provide the following information, where applicable:

- Accession codes, unique identifiers, or web links for publicly available datasets
- A description of any restrictions on data availability
- For clinical datasets or third party data, please ensure that the statement adheres to our policy

Raw sequencing data and processed datasets generated in this study have been deposited in the Gene Expression Omnibus (GEO) under accession number GSE277577. All custom code used to reproduce the analyses and figures presented in this manuscript is available at https://github.com/dcare91/ST_prions (https://doi.org/10.5281/zenodo.18983911). STdeconvolve used in this study is available at. https://github.com/JEFworks-Lab/STdeconvolve.git. Raw immunofluorescence microscopy images are publicly available via Zenodo (https://doi.org/10.5281/zenodo.15824298). The single-cell RNA-seq dataset reanalyzed in this study is available from the Broad Institute Single Cell Portal under study SCP1962. The single-cell RNA sequencing dataset from sorted microglia from 5XFAD mice and controls from Keren-Shaul et al. is available at https://www.ncbi.nlm.nih.gov/geo/query/acc.cgi?acc=GSE98969. The spatial transcriptomics data presented in Figure 6i, was obtained through the online platform strokemap.cn. Human-derived prefrontal cortex cDNA samples used in Fig. 2C were obtained from Prof. Legname's laboratory and were previously described (Vanni S, Moda F, Zattoni M, Bistaffa E, De Cecco E, Rossi M, et al. Differential overexpression of SERPINA3 in human prion diseases. Sci Rep. 2017;7(1):15637); access is subject to the original ethical approvals and material availability.

Research involving human participants, their data, or biological material

Policy information about studies with human participants or human data. See also policy information about sex, gender (identity/presentation), and sexual orientation and race, ethnicity and racism.

Reporting on sex and gender

Biological sex was recorded for human samples analyzed in this study, including post-mortem brain tissue (Figure 2C; Supplementary Table 6) and cerebrospinal fluid and serum samples (Fig. 2E;Supplementary Table 7). Sex was defined as a biological attribute and categorized as male or female based on information provided by the originating clinical studies. Due to limited sample sizes and the observational nature of the cohorts, the study was not powered to assess sex-specific effects and no sex-stratified analyses were performed. Gender identity information was not collected.

Reporting on race, ethnicity, or other socially relevant groupings

Information on race, ethnicity, or other socially relevant groupings was not collected or provided for the human samples used in this study, including those analyzed in Figure 2C and Figure 2E. Accordingly, no analyses were stratified by these variables, and they were not used as covariates or proxies for other factors.

Population characteristics

Human-derived samples used in Figure 2C consisted of post-mortem prefrontal cortex cDNA from patients diagnosed with sporadic Creutzfeldt-Jakob disease (sCJD) and age-matched control individuals, obtained through collaboration with Prof. Legname's laboratory as cDNA product. Available covariate-relevant characteristics included biological sex, age at death, disease status (sCJD or control), and PRNP codon 129 genotype (where available), as reported in Supplementary Table 6.

Human-derived samples used in Figure 2E consisted of cerebrospinal fluid and serum samples from patients diagnosed with sCJD and control individuals, collected as part of previously approved clinical studies. Available covariate-relevant characteristics included biological sex, age at sampling, disease status (sCJD or control), biological material (CSF or serum), and PRNP codon 129 genotype (where available), as reported in Supplementary Table 7.

Owing to the observational study design and limited cohort size, these variables were not used for stratified or multivariable analyses.

Recruitment

Human samples were obtained from previously established clinical cohorts and biobanks under independent ethical approvals. For Fig. 2C, samples were provided through collaboration with Prof. Legname's laboratory. For Fig. 2E, cerebrospinal fluid and serum samples were obtained from sCJD patients and control individuals recruited as part of prior clinical studies. No new participant recruitment was performed for this study. The authors had no role in participant recruitment, enrollment, or assignment, and reused existing samples provided in a blinded manner.

Ethics oversight

All studies involving human-derived samples were conducted under prior ethical approvals, as detailed in the Materials and Methods. Informed consent was obtained from all prion disease patients or their legal next of kin, and the study was approved by the local ethics committee University Medical Center Göttingen, Von Siebold-Str. 3, 37075 Göttingen (No. 24/8/12, No. 11/11/93). Control samples were obtained through other observational studies that were also approved by the same ethics committee at the University Medical Center Göttingen (No. 19/11/09; No. 9/6/08). Samples were blinded to investigators with respect to personal identifiers. Additional human samples used in Figure 2C were obtained under approvals from the relevant institutional review boards of the originating studies. All procedures complied with the Declaration of Helsinki.

Note that full information on the approval of the study protocol must also be provided in the manuscript.

Field-specific reporting

Please select the one below that is the best fit for your research. If you are not sure, read the appropriate sections before making your selection.

☒ Life sciences ☐ Behavioural & social sciences ☐ Ecological, evolutionary & environmental sciences

For a reference copy of the document with all sections, see nature.com/documents/nr-reporting-summary-flat.pdf

Life sciences study design

All studies must disclose on these points even when the disclosure is negative.

Sample size	Sample sizes were determined based on prior experience with the respective experimental systems, availability of biological material, and consistency with previously published studies in the field. No formal statistical methods were used to predetermine sample size. For animal, human, and in vitro experiments, sample sizes were chosen to enable detection of biologically meaningful effects while minimizing the use of animals and limited human-derived material.
Data exclusions	No data exclusion was carried out in this article.
Replication	Key experiments were reproduced across independent biological samples, animals, or cell cultures as indicated in the figure legends and Materials and Methods. In vitro experiments were repeated in multiple independent experiments with technical replicates. Findings from animal-derived samples were validated across independent cohorts (as well as human cohorts) or sample types where available (e.g., brain tissue, cerebrospinal fluid, and serum). All attempts at replication were successful.
Randomization	For animal experiments, mice were assigned to experimental groups based on genotype and treatment condition; no formal randomization was applied. For human-derived samples and in vitro experiments, randomization was not applicable as samples were obtained from predefined cohorts or established cell lines. Potential confounding variables were minimized by consistent experimental handling and analysis procedures across groups.
Blinding	Investigators were not blinded during animal experiments or in vitro assays because experimental conditions were predefined and objectively assessed using standardized protocols. For analyses involving human-derived samples, samples were provided in a blinded manner with respect to personal identifiers, as described in the Materials and Methods. Data analysis was performed using predefined computational pipelines to minimize bias.

Reporting for specific materials, systems and methods

We require information from authors about some types of materials, experimental systems and methods used in many studies. Here, indicate whether each material, system or method listed is relevant to your study. If you are not sure if a list item applies to your research, read the appropriate section before selecting a response.

Materials & experimental systems

n/a	Involved in the study	
☐	☒	Antibodies
☐	☒	Eukaryotic cell lines
☒	☐	Palaeontology and archaeology
☐	☒	Animals and other organisms
☐	☒	Clinical data
☒	☐	Dual use research of concern
☒	☐	Plants

Methods

n/a	Involved in the study	
☒	☐	ChIP-seq
☐	☒	Flow cytometry
☒	☐	MRI-based neuroimaging

Antibodies

Antibodies used

Immunohistochemistry (mouse brain): anti- Prion protein monoclonal SAF84 antibody 1:200 (SPI Bio, A03208), anti- Iba1 1:2500 (Wako, 019-19741), and anti- GFAP 1:1000 (Dako, Z0334).

Immunofluorescence (mouse brain slices): Primary antibodies: anti- Gpnmb 1:200 (Goat, Biotechne, AF2330), anti- Lgals3 1:300 (Rat, Cedarlane, CL8942AP), and anti- Iba1 1:500 (Rabbit, Wako, 019-19741). Secondary antibodies: anti- goat IgG -AF488 1:1000 (Thermo Fisher Scientific, A11055), anti- rat IgG -AF647 1:1000 (Thermo Fisher Scientific, A21247), and anti- rabbit IgG -AF555 1:1000 (Thermo Fisher Scientific, A31572).
Immunofluorescence (iPSC-derived cells): Primary antibodies: anti- MAP2 1:1000 (chicken, Abcam, ab5392), anti- IBA1 1:500 (rabbit, Abcam, ab178846), anti- GFP 1:1000 (mouse, Proteintech, 66002-1-IG), and anti- GPNMB 1:200 (Biotechne, AF2550). Secondary antibodies: anti- goat IgG AF405 1:1000 (Abcam, ab175664), anti- rabbit IgG AF568 1:1000 (Invitrogen, A10042), anti-chicken IgG AF647 1:1000 (Invitrogen, A78952).

FACS staining (nuclei sorting): anti- mouse TruStrain FcX PLUS CD16/32 antibody 1:10 (Biolegend, 156604), anti- NeuN AF488 1:100 (for neuronal nuclei; Millipore MAB377X), anti- PU.1 -PE 1:50 (for microglia nuclei; Cell Signaling Technologies, 81886S), and anti- Olig2 -AF647 1:2000 (for oligodendrocytes nuclei; Abcam, ab225100), anti- LHX2/LH2 1:500 (for astrocytes nuclei; Abcam, ab219983), anti- rabbit IgG -AF647 1:1000 (Thermo Fisher Scientific, A21244).
Flow cytometry staining (apoptotic bodies): PE-Annexin V (BD Biosciences; 556421)

Immunoblotting: anti- GPNMB 1:1000 (Bio-Techne, AF2330), anti- LGALS3 1:2000 (Abcam, ab2785), anti- SPP1 1:3000 (Abcam, ab11503), anti- VIM 1:3000 (Abcam, ab92547), anti- ATP6V0D2 1:2000 (Novus Biologicals, NBP3-10978), anti- NeuN 1:1000 (Abcam, ab177487), anti- VGLUT1 1:1000 (Abcam, ab77822), anti- VGAT 1:500 (Santa Cruz Biotechnology, sc-393373), anti- Olig2 1:1000 (Abcam, ab109186), anti- Iba1 1:1000 (Wako, 016-20001), anti- GFAP 1:10’000 (Dako, Z0334), anti- SYP 1:1000 (BD Biosciences,

	#611880), anti- PrP POM1 300ng/ml (96), anti- Vinculin 1:5000 (Abcam, ab129002), anti- Actin -HRP 1:10'000 (Sigma-Aldrich, A3854), anti- Rabbit IgG -HRP 1:10'000 (Jackson ImmunoResearch, 111.035.045), anti- Mouse IgG -HRP 1:10'000 (Jackson ImmunoResearch, 115.035.003), anti- mouse IgM -HRP 1:1000 (Zymed, 61-6420), anti- Goat IgG -HRP 1:10'000 (Jackson ImmunoResearch, 705.035.147).<br><br>ELISA (human samples): anti- GPNMB 1 µg/mL (Biotechne, AF2550), biotinylated anti- GPNMB 1 µg/mL (Biotechne, BAF2550)<br>ELISA (mouse samples): anti- GPNMB 1 µg/mL (Biotechne, AF2330), biotinylated anti- GPNMB 1 µg/mL (Biotechne, BAF2330)
Validation	All antibodies were commercially sourced and used according to the manufacturers' recommendations for the indicated species and applications. Antibody specificity was supported by expected staining patterns in tissue immunohistochemistry/immunofluorescence, and by detection of bands at the expected molecular weights in immunoblotting. Where applicable, signal specificity was further supported by concordance across independent antibodies/assays targeting the same proteins and by consistency with previously published studies.<br>For POM1 antibody specificity and optimal usage were established in: Polymenidou M, Moos R, Scott M, Sigurdson C, Shi YZ, Yajima B, et al. The POM monoclonals: a comprehensive set of antibodies to non-overlapping prion protein epitopes. PloS one. 2008;3 (12):e3872.

Eukaryotic cell lines

Policy information about cell lines and Sex and Gender in Research

Cell line source(s)	Mouse cell lines:<br>BV-2 (CellosaurusAccession CVCL_0182), mouse microglial cell line, origin: Cytion catalog number 305156.<br>CAD5 is a subclone of catecholaminergic a-differentiated (CAD) cell line showing particular susceptibility to prion infection (Mahal et al., PNAS 2007) were a kind gift from Charles Weissmann.<br>GT1-7 (CellosaurusAccessionCVCL_0281), mouse hypothalamic neuronal cell line, origin: ATCC catalog number CRL-3782.<br><br>Human cell lines:<br>HEK-293T (CellosaurusAccession CVCL_0063), human embryonic kidney cell line, origin: ATCC catalog number CRL-3216.<br><br>iPSCs:<br>iPSC-derived neural networks (iNets) and macrophage precursors were generated from previously established human iPSC lines as described (Hruska-Plochan M, Wiersma VI, Betz KM, Mallona I, Ronchi S, Maniecka Z, et al. A model of human neural networks reveals NPTX2 pathology in ALS and FTLD. Nature. 2024;626(8001):1073-83. van Wilgenburg B, Browne C, Vowles J, and Cowley SA. Efficient, long term production of monocyte-derived macrophages from human pluripotent stem cells under partly-defined and fully-defined conditions. PloS one. 2013;8(8):e71098.).
Authentication	Cells were not authenticated after reception from vendors and collaborators.
Mycoplasma contamination	Cell lines used in this study were tested for mycoplasma contamination (Mycoplasma lookout, cat: MP0035, Sigma-Aldrich) before performing reported experiments, and resulted negative.
Commonly misidentified lines<br>(See ICLAC register)	No commonly misidentified cell lines listed in the ICLAC register were used in this study.

Animals and other research organisms

Policy information about studies involving animals; ARRIVE guidelines recommended for reporting animal research, and Sex and Gender in Research

Laboratory animals	Laboratory mice (Mus musculus) were used. For Visium spatial transcriptomics experiments, 11-week-old mice were inoculated intraperitoneally with RML6 prions or non-infectious brain homogenate (NBH) and sacrificed at 27 weeks post inoculation (wpi), 30 wpi, or terminal stage (31–34 wpi). Animals were housed in IVC cages under a 12/12 h light–dark cycle (07:00–19:00), at 21 ± 1°C and 50 ± 5% humidity, with HEPA-filtered air and sentinel-based health monitoring as described in the Materials and Methods.
Wild animals	This study did not involve wild animals.
Reporting on sex	Sex of the mice used in this study was not recorded and/or not considered as a biological variable in the experimental design, and no sex-stratified analyses were performed.
Field-collected samples	This study did not involve samples collected from the field.
Ethics oversight	Animal experiments were approved by the Veterinary Office of the Canton Zurich (animal permits ZH243/2018, ZH064/2022 and ZH030/2023) and carried out in compliance with the Swiss Animal Protection Law.<br>For the experiment in Figure 2c, prefrontal cortex cDNA from sCJD patients and age-matched controls was kindly provided by Prof. Legname's laboratory. These samples were previously used in one of his published studies under approved ethical permits (Vanni S, Moda F, Zattoni M, Bistaffa E, De Cecco E, Rossi M, et al. Differential overexpression of SERPINA3 in human prion diseases. Sci Rep. 2017;7(1):15637).

Note that full information on the approval of the study protocol must also be provided in the manuscript.

Clinical data

Policy information about clinical studies

All manuscripts should comply with the ICMJE guidelines for publication of clinical research and a completed CONSORT checklist must be included with all submissions.

Clinical trial registration	n/a
Study protocol	n/a
Data collection	n/a
Outcomes	n/a

Plants

Seed stocks	n/a
Novel plant genotypes	n/a
Authentication	n/a

Flow Cytometry

Plots

Confirm that:

- [x] The axis labels state the marker and fluorochrome used (e.g. CD4-FITC).
- [x] The axis scales are clearly visible. Include numbers along axes only for bottom left plot of group (a 'group' is an analysis of identical markers).
- [x] All plots are contour plots with outliers or pseudocolor plots.
- [x] A numerical value for number of cells or percentage (with statistics) is provided.

Methodology

Sample preparation	For apoptotic body detection, conditioned medium from UV-treated or untreated CAD5 cells was incubated with PE-conjugated Annexin V to label phosphatidylserine-positive vesicles. Samples were washed and resuspended in FACS buffer prior to acquisition.<br>For phagocytosis assays, UV-treated CAD5 cells were conjugated with pHrodo Red SE dye and incubated with BV2 cells. After incubation, cells were washed and resuspended in FACS buffer for analysis.<br>For nuclei sorting, nuclei were isolated from flash-frozen half brains of terminal-stage RML6-infected mice using a sucrose-gradient–based protocol. Isolated nuclei were filtered, blocked with Fc receptor–blocking reagent, stained with fluorophore-conjugated antibodies against NeuN, PU.1, Olig2, or LHX2/LH2, counterstained with Hoechst, and resuspended in FACS buffer prior to sorting.
Instrument	Flow cytometry data acquisition was performed using a BD LSRFortessa flow cytometer (BD Biosciences). Fluorescence-activated nuclei sorting was carried out using a BD FACSAria III Cell Sorter (BD Biosciences).
Software	Flow cytometry data were acquired using BD instrument control software and analyzed using FlowJo v10 (Tree Star). No custom software was used for flow cytometry data analysis.
Cell population abundance	For phagocytosis assays, 10,000 events were acquired per sample, and phagocytosis was quantified as the percentage of pHrodo-positive BV2 cells relative to untreated controls. Experiments were performed in three independent biological replicates, each with three technical replicates, and data points represent the mean of technical replicates.<br>For nuclei sorting, cell population abundance was determined based on fluorescence intensity thresholds for lineage-specific markers, following established gating strategies.

Gating strategy	Initial gating was performed on forward and side scatter parameters to exclude debris. For apoptotic body detection, Annexin V–positive events were identified relative to unstained controls. For phagocytosis assays, pHrodo-positive cells were gated based on fluorescence intensity compared to untreated controls.<br>For nuclei sorting, gating followed the strategy described by Nott et al. (Nat. Protoc, 2021), including selection of Hoechst-positive nuclei and subsequent gating on lineage-specific markers (NeuN, PU.1, Olig2, or LHX2/LH2) to define neuronal, microglial, oligodendrocyte, or astrocytic nuclei populations.

☒ Tick this box to confirm that a figure exemplifying the gating strategy is provided in the Supplementary Information.
