# Supplementary material for: Gpnmb defines a phagocytic state of microglia linked to cell death in prion disease mouse model
Source: Nat Commun. 2026 May 12;17:6138. doi: 10.1038/s41467-026-73003-5 (PMC13365222; doi:10.1038/s41467-026-73003-5)
Supplement: Supplementary file 15 — Source data 2 [file 41467_2026_73003_MOESM15_ESM.docx]

**Gpnmb Defines a Phagocytic State of Microglia Linked to Cell Death in Prion Disease Mouse Model**

Source Data 2 – Uncut Wester Blots of Main Figures

**
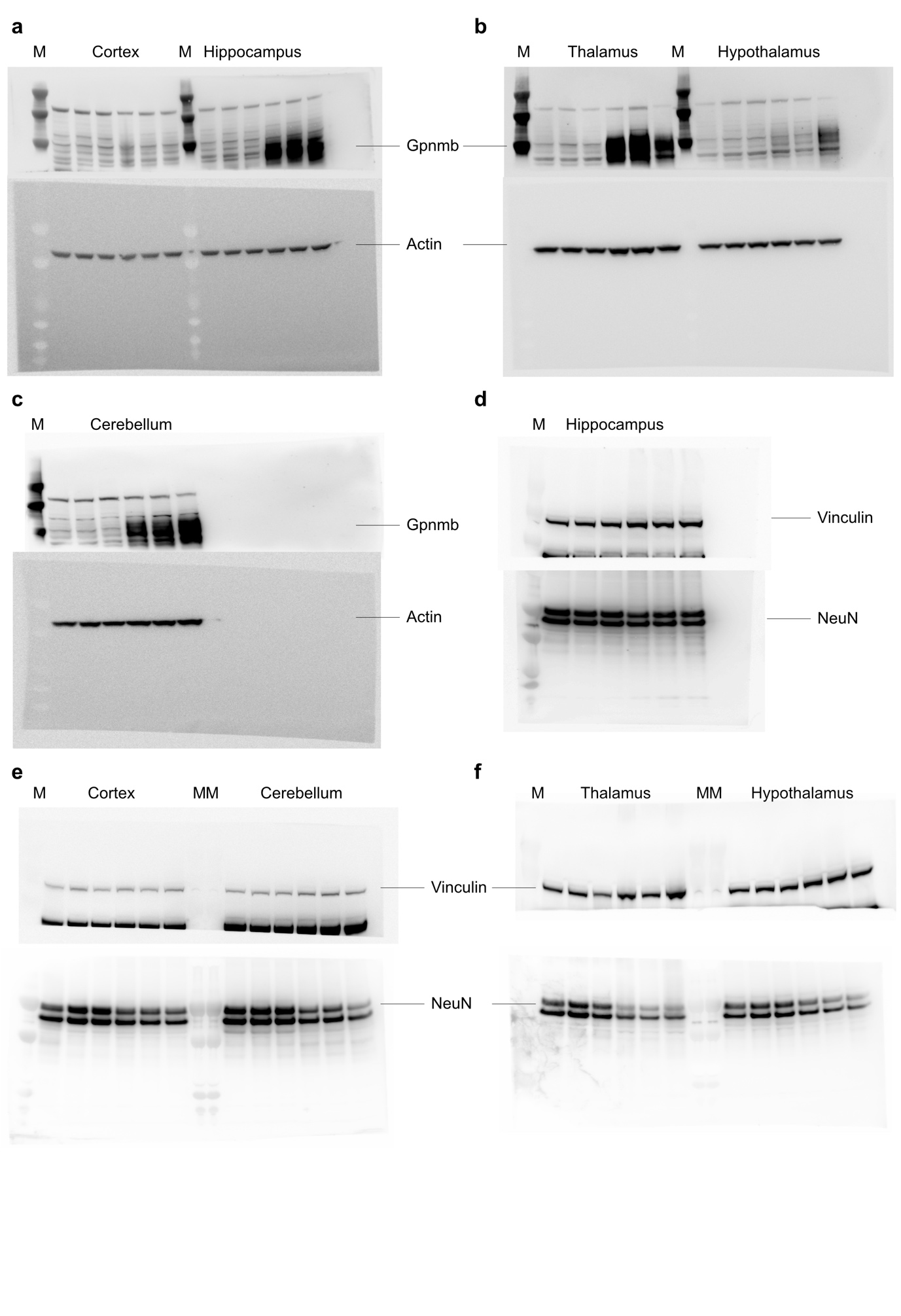
**

**Uncut Western blot of Figure 2b** showing Gpnmb and Actin levels in **a)** cortex and hippocampus, **b)** thalamus and hypothalamus and **c)** cerebellum.

**Uncut Western blots of Figure 5d left panel** showing Vinculin and NeuN in **d)** hippocampus, **e)** cortex and cerebellum and **f)** thalamus and hypothalamus. M represents the molecular weight marker. The membranes were cut in two sections and stained with the targeted antibody.


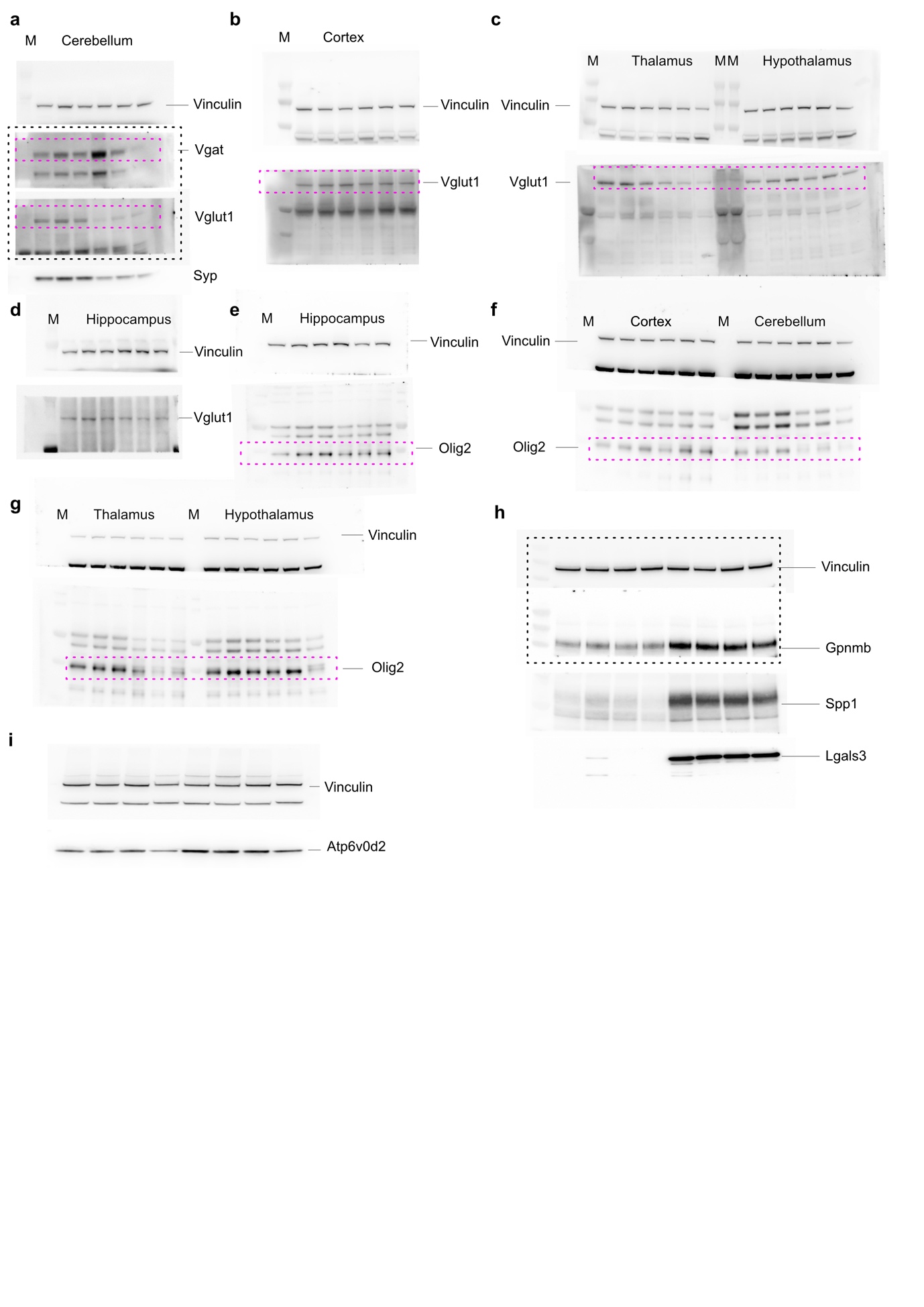


**Uncut Western blot of Figure 5d (middle and right panels)**: **a)** Western blot was divided into three sections and probed for Vinculin (upper section), Vgat and Vglut1 (middle section), and Syp (lower section) in the cerebellum. The black-dotted rectangle indicates the same blot section, initially stained with the Vglut1 antibody, then stripped and re-probed with Vgat. Western blots of Vinculin and Vglut1 **b)** cortex, **c)** thalamus and hypothalamus, **d)** hippocampus. Western blots of Vinculin and Olig2 in **e)** hippocampus, **f)** cortex and cerebellum and **g)** thalamus and hypothalamus

**Uncut Western blots of Figure 6a**: **h)** Gpnmb, Spp1, and Lgals3 were analyzed, with Vinculin used as a loading control. The membrane was divided into three sections. The black-dotted rectangle highlights the same section, where the upper portion was first stained for Gpnmb, then stripped and re-probed for Vinculin. Additionally, uncut Western blot showing **i)** Atp6v0d2 with related Vinculin as loading control on membranes cut in two sections.
