# Supplementary material for: Gpnmb defines a phagocytic state of microglia linked to cell death in prion disease mouse model
Source: Nat Commun. 2026 May 12;17:6138. doi: 10.1038/s41467-026-73003-5 (PMC13365222; doi:10.1038/s41467-026-73003-5)
Supplement: Supplementary file 13 — Transparent Peer Review file [file 41467_2026_73003_MOESM13_ESM.pdf]

nature portfolio

Peer Review File

Gpnmb Defines a Phagocytic State of Microglia Linked to Cell-Death in Prion Disease in Mice

Corresponding Author: Professor Adriano Aguzzi

Parts of this Peer Review File have been redacted as indicated to remove third-party material.

This file contains all reviewer reports in order by version, followed by all author rebuttals in order by version.

Version 0:

Reviewer comments:

Reviewer #1

(Remarks to the Author)
The mechanisms responsible for neurodegeneration observed in prion disease are poorly understood. In this manuscript the authors use an unbiased spatiotemporal transcriptomic approach during disease progression to probe for factors involved in neurodegeneration. The authors examine age-matched RML-infected and mock brain homogenate inoculated animals at the onset of clinical disease and at terminal disease. The authors identify Gpnmb positive subset of microglia enriched in brain areas with the most severe neurodegeneration using a combination of complementary transcriptomic, Western blot, cell culture, and bioinformatic approached to investigate Gpnmb upregulation and activity during prion infection. Additionally, the authors identify a similar upregulation in sCJD cases. These findings are supported by a strong bedrock of data presented in the supplement that further adds to the rigor and conclusions of the authors. Overall, the manuscript addresses an important aspect of prion biology with rigorous, complementary methodology, and the conclusions are supported by the authors. Finally, the discussion is clearly written, and this reviewer appreciates the authors pointing out the limitations of the study.

(Remarks on code availability)

Reviewer #2

(Remarks to the Author)
The study by Caredio et al. investigates how neurodegenerative conditions can drive region-specific microglial states relevant to disease progression. Using spatiotemporal transcriptomic analyses of mice infected with the RML prion strain, the authors identified pronounced neuronal loss and microgliosis in the thalamus and cerebellum, along with the emergence - beginning at 30 weeks post-inoculation - of a Gpnmb$^+$ microglial subpopulation. This state was marked by genes indicating enhanced phagocytic activity and upregulation of lysosomal genes such as Atp6v0d2 and Lgals3. Elevated GPNMB levels were also found in the cerebrospinal fluid of sCJD patients, suggesting potential as a biomarker of disease progression. Mechanistically, the authors hypothesize that GPNMB expression is induced by soluble apoptotic “find-me” signals in both murine BV2 cells and human iPSC-derived microglia exposed to apoptotic neurons, but not by apoptotic bodies or prion accumulation. Based on functional studies in BV2 cells the authors conclude that Gpnmb ablation impairs apoptotic cell clearance, confirming a role in microglial phagocytosis. Collectively, these findings define Gpnmb$^+$ microglia as a phagocytic state that links neural damage to microglial activation and shapes microglial responses during prion disease. This is a well-designed and insightful study that advances understanding of disease-associated microglial states. Nonetheless, several points require clarification or additional consideration to strengthen the conclusions and improve the overall clarity of the findings.
Major points:
1. The novelty of the findings should be presented with more caution. Multiple statements emphasizing novelty are not well justified, as GPNMB$^+$ microglia have already been described in developmental and disease contexts, particularly under conditions involving phagocytosis of lipid-rich material (e.g., PMID: 39992792, PMID: 37433806). This population does not represent a novel microglial state, but rather - as the authors also conclude based on gene set enrichment - one that seems to be associated with lipid/lipoprotein metabolism.
2. The reliance on BV2 cells for some experiments should be interpreted carefully. These cells only partially recapitulate

microglial states and diverge significantly in their responses, particularly to LPS. This limitation should be acknowledged when concluding that LPS does not induce GPNMB expression.
3. Based on the current data, it remains unclear whether GPNMB induction is specific to apoptotic neuron loss. Other stimuli - including Aβ and myelin debris - have been reported to induce GPNMB (e.g., PMID: 39992792, PMID: 37433806). The authors should either experimentally address this or restrict their conclusions to the independence from prion exposure. Presently it would be more accurate to indicate that soluble factors released by damaged neural cells - rather than prion exposure or neuronal loss per se - can drive GPNMB induction.
4. In the immunofluorescence and early-stage prion ST data, much of the Gpnmb signal appears localized to the corpus callosum or cerebellar white matter. As the authors also report oligodendrocyte loss during prion pathology, it remains unclear whether GPNMB$^+$ microglia emerge as a response to neuronal degeneration or to axonal/oligodendrocyte pathology.
5. Relatedly, the limitations of spatial transcriptomic (ST) resolution should be acknowledged. Despite deconvolution, spot-level analysis of the applied assay cannot fully resolve single cells. Markers such as Serpina3n and C4b are also associated with disease-associated oligodendrocyte states (DAO), which complicates interpretation.
Minor points:
1. Reference 79 refers to oligodendrocytes rather than Schwann cells, which are restricted to the peripheral nervous system.
2. The annotation of the re-analyzed Slota et al. dataset appears to include Schwann cells. However, the original study focused on hippocampus and cortex, which do not contain Schwann cells. In that work, these cells were classified as perivascular macrophages, and this should be clarified to avoid misinterpretation.

(Remarks on code availability)

Reviewer #3

(Remarks to the Author)
This manuscript "GPNMB Defines a Phagocytic State of Microglia Linked to Neuronal Loss in Prion Disease" by Caredio et al. attempts to address a novel microglial subtype observed during prion disease and assign function and relevance. The authors present clear and original evidence characterising GPNMB+ microglia and perform key experiments to determine the potential function and also activation factors stimulating these cells. The role of microglia in neurodegenerative diseases and prion disease in particular has been clarified recently, however results given within this manuscript are not presented with full recognition of prior findings. The reviewer also feels critical key experiments are missing in order to prove the authors claim regarding a direct link between emergence of microglial subtype and neuron loss.

Major Comments.
In the introduction the authors state that "Prion-infected, microglia-depleted mouse brains exhibit increased prion seeding activity and spread across the brain." Refs 18-20. Also supported by similar findings in the study by Carroll et al 2018. This finding is not supported however by the observation of unchanged prion accumulation in completely microglia-deficient animals as detailed in (Bradford et al 2022). Indicating that active microglial deletion, most likely apoptotic, during prion disease results in enhanced prion replication. Damisah et al 2020 have shown upregulated astrocyte phagocytosis following active microglial depletion which may be relevant to this observed effect and subsequent interpretations of data later in the presented manuscript.

Whilst the authors present convincing data on the activation and potential function of GPNMB+ microglia the link between the observation of these cells and neuron loss is based on circumstantial evidence of relative timing and proximity of these two observations during in vivo prion disease. These are not convincing and the reviewer requires direct evidence that induction of neuronal apoptosis in vivo stimulates GPNMB+ microglia. These could be undertaken for example via injection of ibotenic acid into the CNS or study of focal induced stroke models for example. At the terminal stage of prion disease apoptosis of various cell types including neurons will be underway, the authors also mention loss of oligodendrocytes and numerous other known pathological changes such as loss of myelin, loss of other neuronal compartments such as dendrites and axons, and also the region-specific activation of astrocytes (Bradford et al. 2019).

Furthermore the observation of unaltered neuron loss in prion-infected microglia-deficient mice (Bradford et al 2022, Figure 3) calls into question the relevance of these observations and limiting them more towards a microglia response to prion infection most likely prompted by apoptotic loss of cells which may include neurons, which is yet to be proven in vivo.

The authors concluding that neuronal loss.. as a key regulator of microglial responses to prion infection. The reviewer disagrees as neuronal loss is a late stage event and microglial activation and proliferation are stimulated by as yet undefined factors well before this. At most this is a minor polarization or subtype of microglia. Though still relevant as recent studies have suggested that balance between microglial subtypes could regulate progression in other neurodegenerative disorders. This is yet to be proven in prion disease where microglia appear to be neuroprotective in action but not essential for the process of prion-induced neurodegeneration.

The reviewer reccomends major revision including more balanced analysis and conclusions drawn from data in light of current published body of evidence regarding microglial activity in prion disease and inclusion of essential in vivo data directly linking neuron apoptosis to GPNMB+ microglia appearance.

Carroll JA, Race B, Williams K, Striebel J, Chesebro B. Microglia Are Critical in Host Defense against Prion Disease. J Virol. 2018 Jul 17;92(15):e00549-18. doi: 10.1128/JVI.00549-18. PMID: 29769333; PMCID: PMC6052316

Bradford, B. M., McGuire, L. I., Hume, D. A., Pridans, C., & Mabbott, N. A. (2022). Microglia deficiency accelerates prion disease but does not enhance prion accumulation in the brain. Glia, 70(11), 2169–2187. https://doi.org/10.1002/glia.24244

Eyiyemisi C. Damisah et al. ,Astrocytes and microglia play orchestrated roles and respect phagocytic territories during neuronal corpse removal in vivo.Sci. Adv.6,eaba3239(2020).DOI:10.1126/sciadv.aba3239

Bradford BM, Wijaya CAW and Mabbott NA (2019) Discrimination of Prion Strain Targeting in the Central Nervous System via Reactive Astrocyte Heterogeneity in CD44 Expression. Front. Cell. Neurosci. 13:411. doi: 10.3389/fncel.2019.00411

(Remarks on code availability)

Version 1:

Reviewer comments:

Reviewer #2

(Remarks to the Author)
The authors have satisfactorily addressed my concerns with additional experiments and clear revisions. These changes strengthen the conclusions and improve the overall clarity and rigor of the manuscript.

(Remarks on code availability)

Reviewer #3

(Remarks to the Author)
The revised manuscript "Gpnmb Defines a Phagocytic State of Microglia Linked to Cell-Death in Prion Disease" provides a much more balanced and conclusive analysis of their findings and benefits greatly from additional material and work undertaken. The authors have definitively addressed all previous major concerns by providing further independent confirmation from other models and defined more specifically their results. In so doing they present data on a general mechanism of induction of a specific microglial state (GPNMB+) with much broader implications for neurodegenerative conditions generally.

(Remarks on code availability)

Open Access This Peer Review File is licensed under a Creative Commons Attribution 4.0 International License, which permits use, sharing, adaptation, distribution and reproduction in any medium or format, as long as you give appropriate credit to the original author(s) and the source, provide a link to the Creative Commons license, and indicate if changes were made.
In cases where reviewers are anonymous, credit should be given to 'Anonymous Referee' and the source.
The images or other third party material in this Peer Review File are included in the article's Creative Commons license, unless indicated otherwise in a credit line to the material. If material is not included in the article's Creative Commons license and your intended use is not permitted by statutory regulation or exceeds the permitted use, you will need to obtain permission directly from the copyright holder.
To view a copy of this license, visit https://creativecommons.org/licenses/by/4.0/

Full revision

Reviewer #1

Remarks to the authors:

The mechanisms responsible for neurodegeneration observed in prion disease are poorly understood. In this manuscript the authors use an unbiased spatiotemporal transcriptomic approach during disease progression to probe for factors involved in neurodegeneration. The authors examine age-matched RML-infected and mock brain homogenate inoculated animals at the onset of clinical disease and at terminal disease. The authors identify Gpnmb positive subset of microglia enriched in brain areas with the most severe neurodegeneration using a combination of complementary transcriptomic, Western blot, cell culture, and bioinformatic approached to investigate Gpnmb upregulation and activity during prion infection. Additionally, the authors identify a similar upregulation in sCJD cases. These findings are supported by a strong bedrock of data presented in the supplement that further adds to the rigor and conclusions of the authors. Overall, the manuscript addresses an important aspect of prion biology with rigorous, complementary methodology, and the conclusions are supported by the authors. Finally, the discussion is clearly written, and this reviewer appreciates the authors pointing out the limitations of the study.

Major comments:

[no major comments presented]

None

Minor comments:

[no minor comments presented]

None

Reviewer #2

Remarks to the authors:

The study by Caredio et al. investigates how neurodegenerative conditions can drive region-specific microglial states relevant to disease progression. Using spatiotemporal transcriptomic analyses of mice infected with the RML prion strain, the authors identified pronounced neuronal loss and microgliosis in the thalamus and cerebellum, along with the emergence - beginning at 30 weeks post-inoculation - of a Gpnmb$^+$ microglial subpopulation. This state was marked by genes indicating enhanced phagocytic activity and upregulation of lysosomal genes such as Atp6v0d2 and Lgals3. Elevated GPNMB levels were also found in the cerebrospinal fluid of sCJD patients, suggesting potential as a biomarker of disease progression. Mechanistically, the authors hypothesize that GPNMB expression is induced by soluble apoptotic “find-me” signals in both murine BV2 cells and human iPSC-derived microglia exposed to apoptotic neurons, but not by apoptotic bodies or prion accumulation. Based on functional studies in BV2 cells the authors conclude that Gpnmb ablation impairs apoptotic cell clearance, confirming a role in microglial phagocytosis. Collectively, these

findings define Gpnmb$^+$ microglia as a phagocytic state that links neural damage to microglial activation and shapes microglial responses during prion disease.
This is a well-designed and insightful study that advances understanding of disease-associated microglial states. Nonetheless, several points require clarification or additional consideration to strengthen the conclusions and improve the overall clarity of the findings.

Major comments:

1) The novelty of the findings should be presented with more caution. Multiple statements emphasizing novelty are not well justified, as GPNMB$^+$ microglia have already been described in developmental and disease contexts, particularly under conditions involving phagocytosis of lipid-rich material (e.g., PMID: 39992792, PMID: 37433806). This population does not represent a novel microglial state, but rather - as the authors also conclude based on gene set enrichment - one that seems to be associated with lipid/lipoprotein metabolism.

GPNMB$^+$ microglia have been described previously in both developmental and disease contexts, particularly involving phagocytosis of lipid-rich material such as myelin debris (e.g. Liut et al. (1), Androvic et al. (2)). We have removed statements implying discovery of a previously unrecognized microglial subtype.
The novelty of our findings lies in identifying a unifying upstream principle governing Gpnmb induction. Through in vivo and in vitro experiments, human co-cultures, and transcriptomic analyses, we now show that Gpnmb is induced by soluble cues associated with cell death, independent of the cellular identity of the dying cell (neuronal, oligodendroglial, or microglial) but not by direct exposure to protein aggregates or inflammatory stimuli. Our data therefore position Gpnmb within a broader damage-responsive, phagocytosis-associated microglial program.
Consistent with our data and prior literature (2, 3), we find that Gpnmb induction co-segregates with lipid and lipoprotein metabolism pathways. Our study highlights the necessity of rigorously controlling for treatment-induced cell death when interpreting microglial activation states, as microglial Gpnmb upregulation appears to arise as a secondary consequence of cellular toxicity. By dissociating direct sensing from injury-driven effects, our work reconciles disparate observations across developmental, neurodegenerative, and injury models within a single mechanistic paradigm.

2) The reliance on BV2 cells for some experiments should be interpreted carefully. These cells only partially recapitulate microglial states and diverge significantly in their responses, particularly to LPS. This limitation should be acknowledged when concluding that LPS does not induce GPNMB expression.

We have now extended our analysis to a human neuron-primary-like microglia co-culture system, where LPS likewise failed to induce GPNMB expression, confirming that our conclusion does not rely on BV2-specific behavior.

3) Based on the current data, it remains unclear whether GPNMB induction is specific to apoptotic neuron loss. Other stimuli - including Aβ and myelin debris - have been reported to induce GPNMB (e.g., PMID: 39992792, PMID: 37433806). The authors should either experimentally address this or restrict their conclusions to the independence from prion exposure. Presently it would be more accurate to indicate that soluble factors released by damaged neural cells - rather than prion exposure or neuronal loss per se - can drive GPNMB induction.

Liu et al. (PMID: 3999279 (1) reported increased microglial GPNMB after hippocampal injection of Aβ, suggesting that other aggregates beyond prions may induce its expression.

However, in that study GPNMB upregulation followed injection of Aβ monomers – not aggregated or fibrillar species (Fig. 1L–M) – and cell death from high local protein concentration was not assessed. We therefore interpret these findings as consistent with our model: GPNMB induction reflects tissue damage and release of death-associated cues, rather than direct sensing of amyloid. In this framework, Aβ monomer load may act indirectly by causing neural injury, which then triggers a phagocytic Gpnmb-upregulated microglia response.

To directly test this, we treated BV2 microglia-like cells with increasing concentrations of recombinant $A\beta_{1-42}$ fibrils (0, 70, 200, 500 nM). Fibril formation was confirmed by transmission electron microscopy (Supplementary Fig. 16a). After 48 h of exposure (matching the incubation time used for apoptotic body-conditioned medium) no increase in Gpnmb expression was observed under any condition (Supplementary Figure 16b-c). To exclude confounding effects from treatment-induced toxicity, we performed parallel cell-viability assays, which revealed no significant differences in viability across conditions (Supplementary Figure 16d).

To test whether this finding holds in physiologically relevant systems, we performed the same Aβ fibril treatments in human Neurons–primary-like microglia (iNets-pMG) co-cultures. After 48 h of exposure, GPNMB levels in microglia remained unchanged across all Aβ conditions (Supplementary Figure 17a and c) and no neuronal loss was detected (Supplementary Figure 17b and d), in line with Dolan et al. 2023 (4) reporting that GPNMB was induced in response to apoptotic neurons and myelin debris, but not to Aβ fibrils or synaptosomes. We argue that Liu et al. 2025 actually supports our claim that cell death is the main driver of GPNMB induction in microglia as shown in Figure 1N (1) and that GPNMB expression is required for efficient microglia phagocytosis.

[REDACTED]

Figure 1 from Liu et al. 2025. (L) Experimental schematic showing infusion of fluorescent Aβ (FITC-Aβ) or debris (CFSE debris) into the hippocampus. (M) Representative confocal images of co-labeling of Iba1, Aβ, and GPNMB 24 and 48 h post Aβ infusion. Scale bar: 50 µm. (N) Representative confocal images of co-labeling of Iba1, debris, and GPNMB 16, 24, 36, and 48 h post debris infusion. Scale bar: 50 µm. (O–Q) Statistics of the percentage of Iba1+GPNMB+ cells among GPNMB+ cells (O), Iba1+GPNMB+debris+ cells among Iba1+GPNMB+ cells (P), and Iba1+GPNMB+debris+ cells among Iba1+debris+ cells (Q) 48 h after debris infusion. Data are represented as mean ± SEM.

If Gpnmb induction reflected a direct microglial response to amyloid exposure, its upregulation should be detectable at earlier disease stages, when amyloid species are already present but overt neuronal loss is minimal. However, across multiple independent single-cell datasets, including the time-resolved AD dataset that we have reanalysed here (Supplementary Figure 18) (5), microglia expressing classical amyloid-responsive markers (e.g. *Apoe*, *Trem2*, *Clec7a*) are already detectable at early timepoints, whereas *Gpnmb*-positive microglia emerge only at later stages, coinciding with active neuronal loss. These temporal dynamics support our conclusion that Gpnmb upregulation is coupled to ongoing neuronal damage and phagocytic engagement, rather than to amyloid sensing per se.

We also revised our original interpretation and now conclude that GPNMB induction is not specific to neuronal loss. Androvic et.al [PMID: 37433806] (2) found that focal myelin loss is sufficient to elicit Gpnmb-positive microglia within lesions, indicating that this response extends beyond neuron-specific injury. We update our framework to propose a more general mechanism whereby cell death-associated signals, independent of cellular identity, drive microglial Gpnmb upregulation.

Accordingly, conditioned medium from dying microglia was sufficient to induce Gpnmb expression in naïve BV2 cells, demonstrating that soluble factors released during cell death alone are sufficient to trigger this response (Supplementary Figure 13f). Together with our apoptotic neuron and myelin debris data (2), these findings support a unifying framework in which Gpnmb induction reflects engagement of microglia with dying or damaged cellular material, rather than a response to a specific cell type or aggregate species. In line with this refined conceptual framework, we have revised the title from "*Gpnmb Defines a Phagocytic State of Microglia Linked to Neuronal Loss in Prion Disease*" to "*Gpnmb Defines a Phagocytic State of Microglia Linked to Cell-Death in Prion Disease*".

4) In the immunofluorescence and early-stage prion ST data, much of the Gpnmb signal appears localized to the corpus callosum or cerebellar white matter. As the authors also report oligodendrocyte loss during prion pathology, it remains unclear whether GPNMB$^+$ microglia emerge as a response to neuronal degeneration or to axonal/oligodendrocyte pathology.

The spatial enrichment of Gpnmb signal within white matter regions makes it difficult to attribute the emergence of GPNMB$^+$ microglia exclusively to neuronal degeneration. This critique prompted additional experimentation, which ultimately led us to revise our interpretation. We now propose that microglial Gpnmb upregulation is triggered by soluble cues associated with local cell death, irrespective of cellular identity, including neuronal, oligodendroglial, or other neural cells. To address this issue more broadly, we complemented our spatial and immunofluorescence analyses with mechanistic experiments demonstrating that conditioned medium derived from dying cells (including non-neuronal sources) suffices to induce Gpnmb expression in microglia (Supplementary Figure 13f). Together with published evidence showing GPNMB$^+$ microglia in settings of myelin loss, these data support a unified framework in which GPNMB marks a damage-responsive, phagocytosis-associated microglial program, rather than a response to a specific degenerating cell type.

Accordingly, we have revised the manuscript to remove statements implying neuronal specificity and to emphasize that GPNMB induction reflects engagement with local tissue damage in regions of active neurodegeneration, including white matter tracts.

5) Relatedly, the limitations of spatial transcriptomic (ST) resolution should be acknowledged. Despite deconvolution, spot-level analysis of the applied assay cannot fully resolve single cells. Markers such as Serpina3n and C4b are also associated with disease-associated oligodendrocyte states (DAO), which complicates interpretation.

We now acknowledge this limitation in the Discussion: "*Indeed in the discussion I have now included this: In addition, current spatial transcriptomic approach is limited by spot-level resolution, which, even with deconvolution, cannot fully resolve single-cell states in densely populated regions. This is particularly relevant for markers such as Serpina3n and C4b, which are also associated with disease-associated oligodendrocyte (DAO) states and may complicate cell-type attribution. Future studies integrating higher-resolution spatial methods with single-cell transcriptomics and orthogonal functional validation will be required to precisely define cell-type–specific roles of Gpnmb in neurodegenerative tissue remodeling.*"

Minor comments:

1) Reference 79 refers to oligodendrocytes rather than Schwann cells, which are restricted to the peripheral nervous system.

The sentence was corrected according to reviewer's comment: *"As in our immunofluorescence, its co-upregulation with Lgals3 has been observed in proliferative region-associated microglia, which engulf oligodendrocytes in developing white matter (6, 7), and in TGFβ-signaling-deficient microglia, where enhanced phagocytic activity facilitates the removal of dying oligodendrocytes during demyelination (7)."*

2) The annotation of the re-analyzed Slota et al. dataset appears to include Schwann cells. However, the original study focused on hippocampus and cortex, which do not contain Schwann cells. In that work, these cells were classified as perivascular macrophages, and this should be clarified to avoid misinterpretation.

We thank the reviewer for pointing this out. We agree that Schwann cells are not expected in hippocampal or cortical parenchyma, as analyzed in the original Slota et al. study (8), and that cells previously labeled as "Schwann cells" in our re-analysis most likely correspond to perivascular macrophages, consistent with the original annotation. In the revised manuscript, we have corrected this classification and now refer to these cells as perivascular macrophages in Supplementary Figure 8.

Reviewer #3

Remarks to the authors:

This manuscript "GPNMB Defines a Phagocytic State of Microglia Linked to Neuronal Loss in Prion Disease" by Caredio et al. attempts to address a novel microglial subtype observed during prion disease and assign function and relevance. The authors present clear and original evidence characterising GPNMB+ microglia and perform key experiments to determine the potential function and also activation factors stimulating these cells. The role of microglia in neurodegenerative diseases and prion disease in particular has been clarified recently, however results given within this manuscript are not presented with full recognition of prior findings. The reviewer also feels critical key experiments are missing in order to prove the authors claim regarding a direct link between emergence of microglial subtype and neuron loss.

Major comments:

1) In the introduction the authors state that "Prion-infected, microglia-depleted mouse brains exhibit increased prion seeding activity and spread across the brain." Refs 18-20. Also supported by similar findings in the study by Carroll et al 2018. This finding is not supported however by the observation of unchanged prion accumulation in completely microglia-deficient

animals as detailed in (Bradford et al 2022). Indicating that active microglial deletion, most likely apoptotic, during prion disease results in enhanced prion replication. Damisah et al 2020 have shown upregulated astrocyte phagocytosis following active microglial depletion which may be relevant to this observed effect and subsequent interpretations of data later in the presented manuscript.

The contribution of microglia to prion disease progression remains complex and, in some respects, model-dependent. Various studies report either neuroprotective (9) or neurotoxic effects of microglia in prion disease (10, 11). These discrepancies may reflect differences in microglial depletion strategies, timing, and the coexistence of functionally distinct microglial subpopulations whose relative abundance may lead to divergent overall outcomes. We now highlight this point in the Introduction. We have therefore restructured the Introduction to accurately reflect the current state of the literature, incorporating the suggested references (12-14) and explicitly acknowledging the coordinated roles of microglia and astrocytes in phagocytosis of apoptotic neurons, as well as microglial modulation of astrocytic responses during prion disease:
"Prion-infected, microglia-depleted mouse brains exhibit increased neurotoxicity and accelerated disease progression, suggesting a role for microglia in prion clearance and containment (9, 12). Recent studies also indicate that microglia and astrocytes can cooperate in a highly coordinated manner to maintain tissue homeostasis in PrD, particularly by jointly phagocytosing apoptotic neurons, with microglia actively modulating astrocyte activity (10, 11)."
2) Whilst the authors present convincing data on the activation and potential function of GPNMB$^+$ microglia the link between the observation of these cells and neuron loss is based on circumstantial evidence of relative timing and proximity of these two observations during in vivo prion disease. These are not convincing and the reviewer requires direct evidence that induction of neuronal apoptosis in vivo stimulates GPNMB+ microglia. These could be undertaken for example via injection of ibotenic acid into the CNS or study of focal induced stroke models for example. At the terminal stage of prion disease apoptosis of various cell types including neurons will be underway, the authors also mention loss of oligodendrocytes and numerous other known pathological changes such as loss of myelin, loss of other neuronal compartments such as dendrites and axons, and also the region-specific activation of astrocytes (Bradford et al. 2019).

We agree that, in the context of prion disease alone, correlations based on relative timing and spatial proximity are insufficient to establish a causal link between cell-death and the emergence of GPNMB$^+$ microglia. We now address this concern with two approaches:

1. We have now included independent in vivo models of acute, spatially restricted cell death, thereby isolating apoptotic injury from the multifactorial pathology of prion disease. We employed a middle cerebral artery occlusion (MCAO) stroke model by injecting human alpha-thrombin, a well-established paradigm of focal ischemic neuronal death (15). In this setting, Gpnmb$^+$ microglia emerge specifically within infarcted regions and co-localize with established phagocytic markers such as Lgals3 (Figure 6f-h).
2. To further support this observation with spatial transcriptomic evidence, we analysed an independent, publicly available spatial transcriptomics dataset (http://www.strokemap.cn) (16), generated using a distinct stroke-induction approach (Rose Bengal-based photothrombotic stroke strategy). Consistent with our immunofluorescence data, this dataset shows strong enrichment of Gpnmb expression in peri-infarct regions, with markedly lower expression in non-infarcted tissue (Figure 6i).

These complementary approaches decouple apoptotic injury from the complex and progressive pathological features of terminal prion disease, including oligodendrocyte loss, myelin degeneration and axonal damage.
Together, our data provide direct in vivo evidence that focal cell death is sufficient to induce Gpnmb expression in microglia, supporting a model in which GPNMB marks a damage-responsive, phagocytosis-associated microglial program rather than a prion-specific response.

3) Furthermore, the observation of unaltered neuron loss in prion-infected microglia-deficient mice (Bradford et al 2022, Figure 3) calls into question the relevance of these observations and limiting them more towards a microglia response to prion infection most likely prompted by apoptotic loss of cells which may include neurons, which is yet to be proven in vivo.

The observation of unaltered hippocampal CA1 neuronal density (despite reduced, but not significantly) in microglia-deficient ME7-infected mice (Bradford et al. 2022) (14) does not directly impact the interpretation of our findings. We do not propose that GPNMB$^+$ microglia are required to limit neuronal loss, nor that microglial depletion should necessarily exacerbate neuronal degeneration. Rather, our central conclusion is that Gpnmb induction reflects a microglial state in response to ongoing cell death rather than to prion load directly, independent of whether microglia modulate the overall extent of neuronal loss.
Importantly, neuronal density measurements restricted to CA1 at terminal disease stages do not exclude earlier or other brain region-specific apoptosis, including in non-neuronal cells. This is particularly relevant given that regional vulnerability differs substantially across prion strains and experimental paradigms. Whereas Bradford et al. employed intracerebral inoculation of ME7 strain, we used intraperitoneal, which preferentially affects thalamic and cerebellar regions (17). Such differences in strain tropism and route of infection produce distinct neuroanatomical degeneration patterns and limit direct comparison of hippocampal-only endpoints.
Crucially, our conclusions do not rely solely on correlative data in prion disease alone. We now provide direct in vivo evidence that focal induction of cell-death upon ischemic injury is sufficient to elicit Gpnmb$^+$ microglia, thereby demonstrating that cell death itself is sufficient to trigger this microglial program, independent of prion strain, route of inoculation, or disease-specific pathology.

[REDACTED]

Figure 3 Bradford et al 2022. Microglia-deficiency effects on prion-specific vacuolation and prion accumulation. (a) Hematoxylin and eosin stained hippocampus CA1 of terminal prion infected or age-matched uninfected Csf1rWT or Csf1rΔFIRE mice. Scale bars = 200 µm. (b) Hippocampal CA1 pyramidal cell density in terminal prion infected Csf1rWT or Csf1rΔFIRE mice. Student's t-test. (c) Assessment of neuronal condition expressed as percentage of total neurons pyknotic in terminal prion infected Csf1rWT or Csf1rΔFIRE mice. Student's t-test.

4) The authors concluding that neuronal loss.. as a key regulator of microglial responses to prion infection. The reviewer disagrees as neuronal loss is a late stage event and microglial activation and proliferation are stimulated by as yet undefined factors well before this. At most this is a minor polarization or subtype of microglia. Though still relevant as recent studies have suggested that balance between microglial subtypes could regulate progression in other neurodegenerative disorders. This is yet to be proven in prion disease where microglia appear to be neuroprotective in action but not essential for the process of prion-induced neurodegeneration.

Microgliosis is a well-established early event in prion disease, occurring well before overt neuronal loss. Accordingly, in Figure 4c we observe robust microglial expansion at early time points, yet Gpnmb expression remains unchanged, indicating that Gpnmb induction is not a feature of early microglial activation.

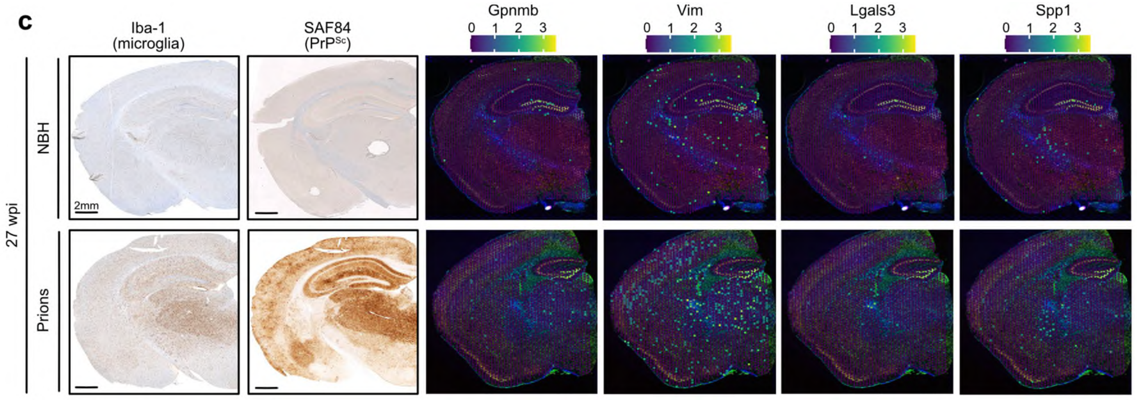

Figure 4. Single-cell and spatial association of Gpnmb with disease-associated microglial markers in prion disease. c) Immunohistochemical (IHC) staining of brain sections at 27 wpi (scale bar: 2 mm) shows the microglial marker Iba-1 and PrP^{Sc} (SAF84), with spatial transcriptomics data illustrating the expression of *Gpnmb*, *Vim*, *Lgals3* and Spp1.

We revised our interpretation to state that cell death – rather than neuronal loss specifically – drives the emergence of a distinct, late-stage *Gpnmb*$^+$ microglial state. We now clarify that this program reflects a cell death-associated response that occurs only at advance disease stages and is not equivalent to global microglial activation. We further emphasize that neuronal loss is a late feature of prion disease, as in all neurodegenerative disorders, and have now revised the manuscript to make this temporal distinction even more explicit, thereby distinguishing apoptosis-associated *Gpnmb*-induction from earlier activation states.
To address this point, we have rephrased the abstract and multiple sections of the Discussion to emphasize that *Gpnmb*$^+$ microglia represent a late-emerging, apoptosis-driven phagocytic state within the broader spectrum of activated microglia. The revised abstract now states: "*This work defines Gpnmb*$^+$ *microglia as an apoptosis-driven phagocytic state that links cell death to microglial activation and reveals a generalizable mechanism by which microglia respond to cell loss.*"
In the Discussion, we now state that Gpnmb-associated transcriptional changes emerge only at terminal stages, when prion deposition and gliosis are already widespread, pointing to secondary consequences of tissue injury rather than direct responses to prion replication. We further show that Gpnmb$^+$ microglia localize to regions with the highest neuronal and oligodendroglial loss and exhibit transcriptional programs enriched for apoptotic cell recognition, lipid metabolism, and lysosomal function, features consistent with phagocytic stress rather than early microglial activation.
The balance between microglial subtypes may influence disease progression, as suggested in other neurodegenerative disorders. Our data provide causal in vivo evidence that tissue degeneration and apoptotic burden are sufficient to induce the Gpnmb$^+$ microglial state. This

does not imply that microglia are essential drivers of prion-induced neurodegeneration, but rather that Gpnmb^{+} microglia represent a context-dependent, late-stage response to extreme clearance demand.

Minor comments:

[no minor comments presented]

none

Modified and/or added figures in the resubmitted manuscript were the following:

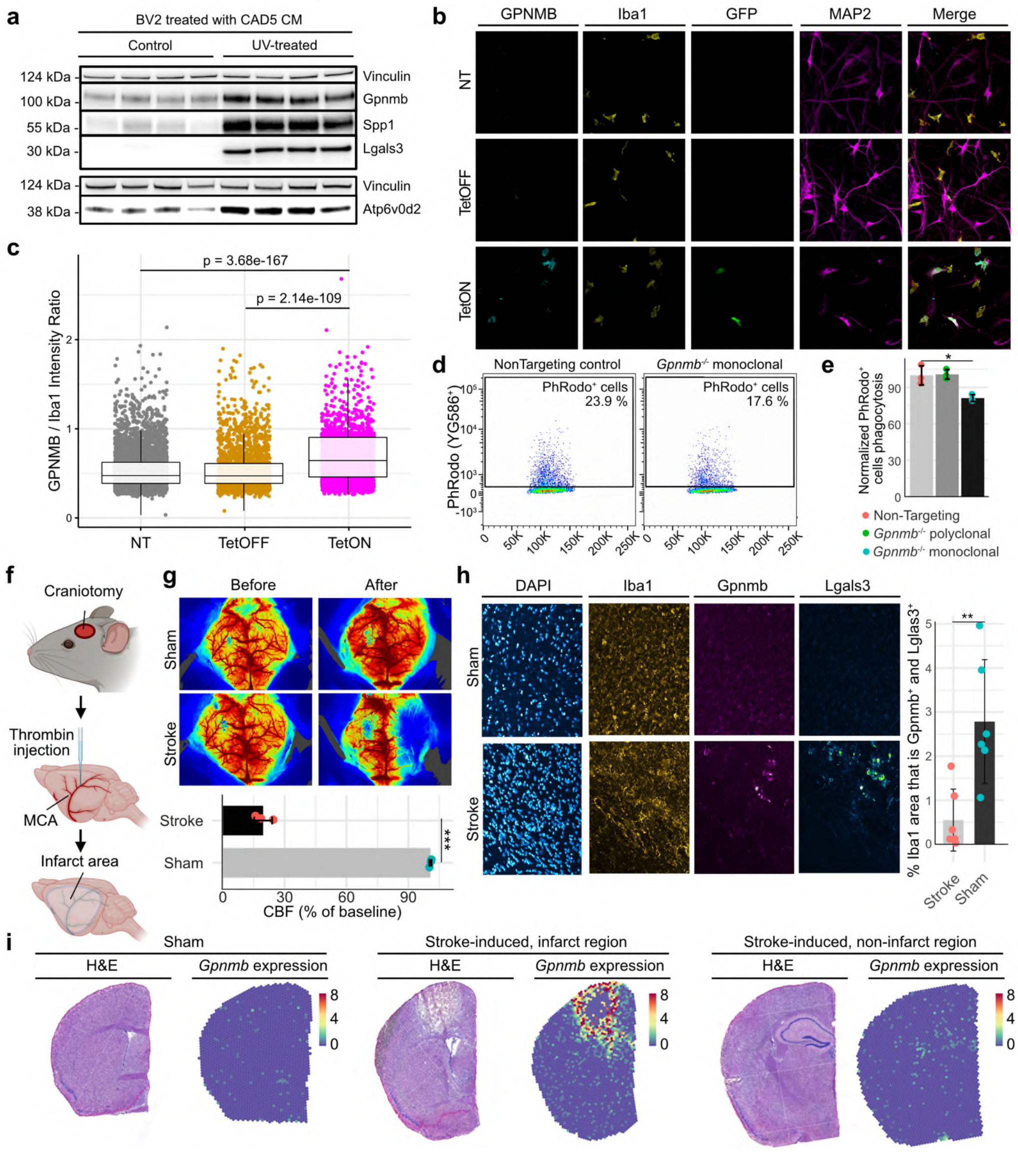

Figure 6. Gpnmb expression associated with apoptotic sensing and ischemic injury in microglia. a) Western blot of BV2 cells treated with conditioned medium (CM) from UV-treated CAD5 cells to generate apoptotic bodies, and conditioned medium from healthy CAD5 cells for control. Gpnmb, Spp1, Lgals3 and Atp6v0d2 protein levels compared to the control protein Vinculin. **b)** Gpnmb (cyan), Iba1 (yellow, microglia marker), EGFP (green, labeling DOX-dependent expression), and MAP2 (magenta, neuronal marker). Upper row corresponds to non-treated condition (NT). Middle row corresponds to the TetOFF condition. Lower row corresponds to the TetON condition, where doxycycline is present to induce EGFP expression inducing neuronal death. **c)** Quantification of Gpnmb mean fluorescence intensity normalized to Iba1 mean fluorescence intensity across NT, DOX-OFF and DOX-ON conditions. Individual data points represent single microglia cells. Pairwise comparisons were performed using two-sided Wilcoxon rank-sum tests with Benjamini-Hochberg correction. **d)** Flow cytometry analysis of phagocytic activity in BV2 microglial cells. Cas9-expressing BV2 cells were transduced with either a non-targeting plasmid (control) or single guide RNA targeting *Gpnmb* gene, followed by selection of a monoclonal population. Cells were incubated with Phrodo-labeled apoptotic CAD5 cells. **e)** Quantification of phagocytic activity (Phrodo^{+} cells) in non-targeting control, *Gpnmb*-depleted BV2 polyclonal, and *Gpnmb*-depleted monoclonal cells (*$p < 0.05$). **f)** Schematic of the middle cerebral artery occlusion (MCAO) stroke model. **g)** Cerebral blood flow (CBF) measurements before and after MCAO, displayed as heatmaps (laser speckle imaging) and quantified relative to baseline. **h)** Immunofluorescence staining of sham and stroke brain sections showing DAPI (nuclei), Iba1^{+} (microglia), and Gpnmb and Lgals3 expression, with related triple pixel colocalization quantification. **i)** Representative H&E-stained brain sections from sham animals and stroke-induced infarct and non-infarct regions, overlaid with spatial maps of Gpnmb expression intensity. Statistical significance (*$p < 0.05$, **$p < 0.01$, ***$p < 0.005$, ****$p < 0.001$) is indicated by asterisks.

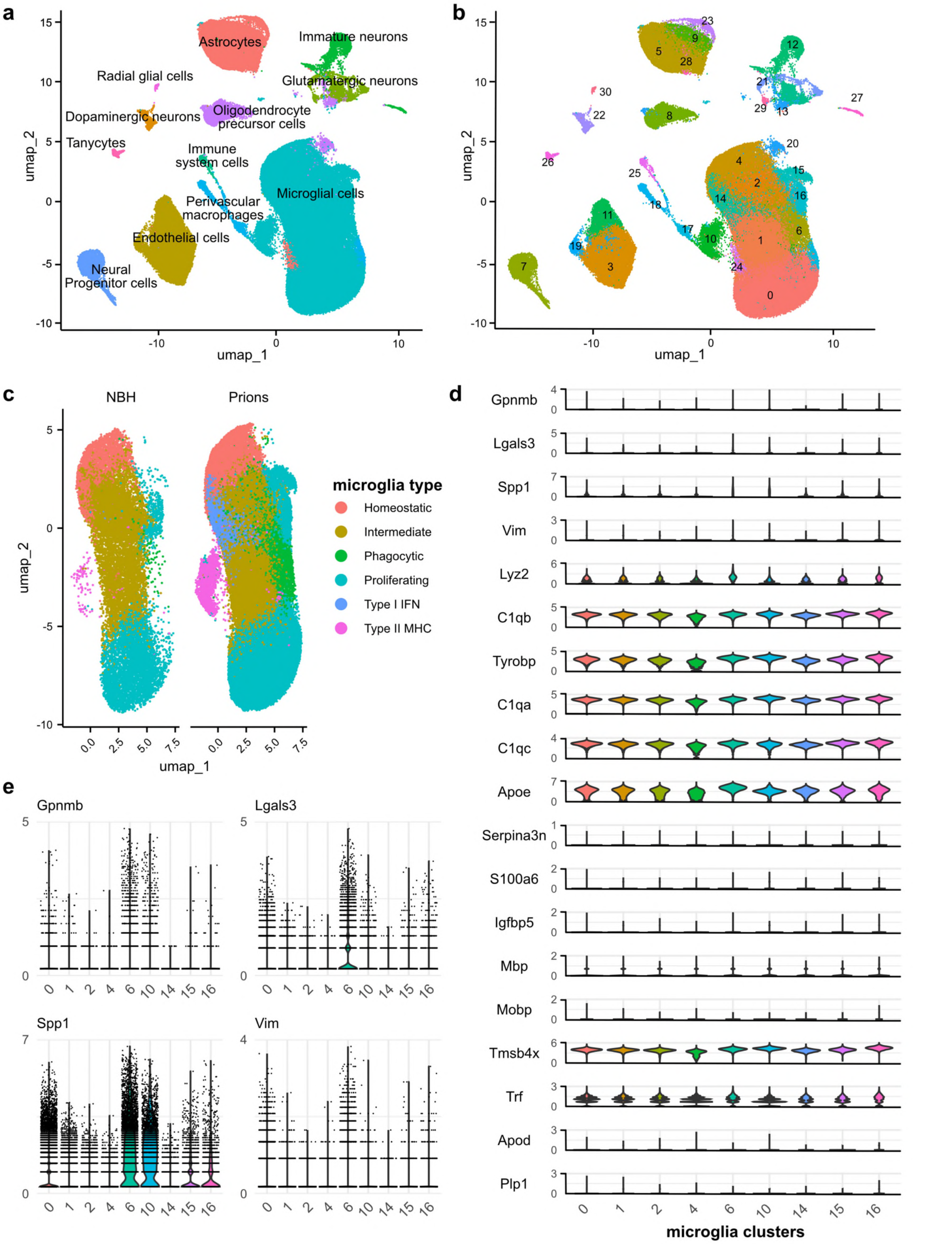

Supplementary Figure 8. This figure presents a reanalysis of single-cell RNA sequencing data of terminally ill prion infected mice from Slota et al., 2022. **a)** UMAP plots illustrating the distribution of major CNS cell types, including microglia, astrocytes, neurons, oligodendrocyte precursor cells and others. **b)** UMAP plots showing clustering of major CNS cell types into distinct subtypes, with labels indicating specific clusters. **c)** Comparative UMAP plots of microglial cells from NBH and prion-infected conditions, categorized into homeostatic, intermediate, phagocytic, proliferating, type I IFN and type II MHC microglia. **d)** Violin plots displaying the expression levels of the 19 genes obtained from the overlap of the two prion-related replicates by using STdeconvolve statistical model. **e)** Dot plots showing gene expression levels for *Gpnmb*, *Lgals3*, *Spp1* and *Vim* across microglial clusters.

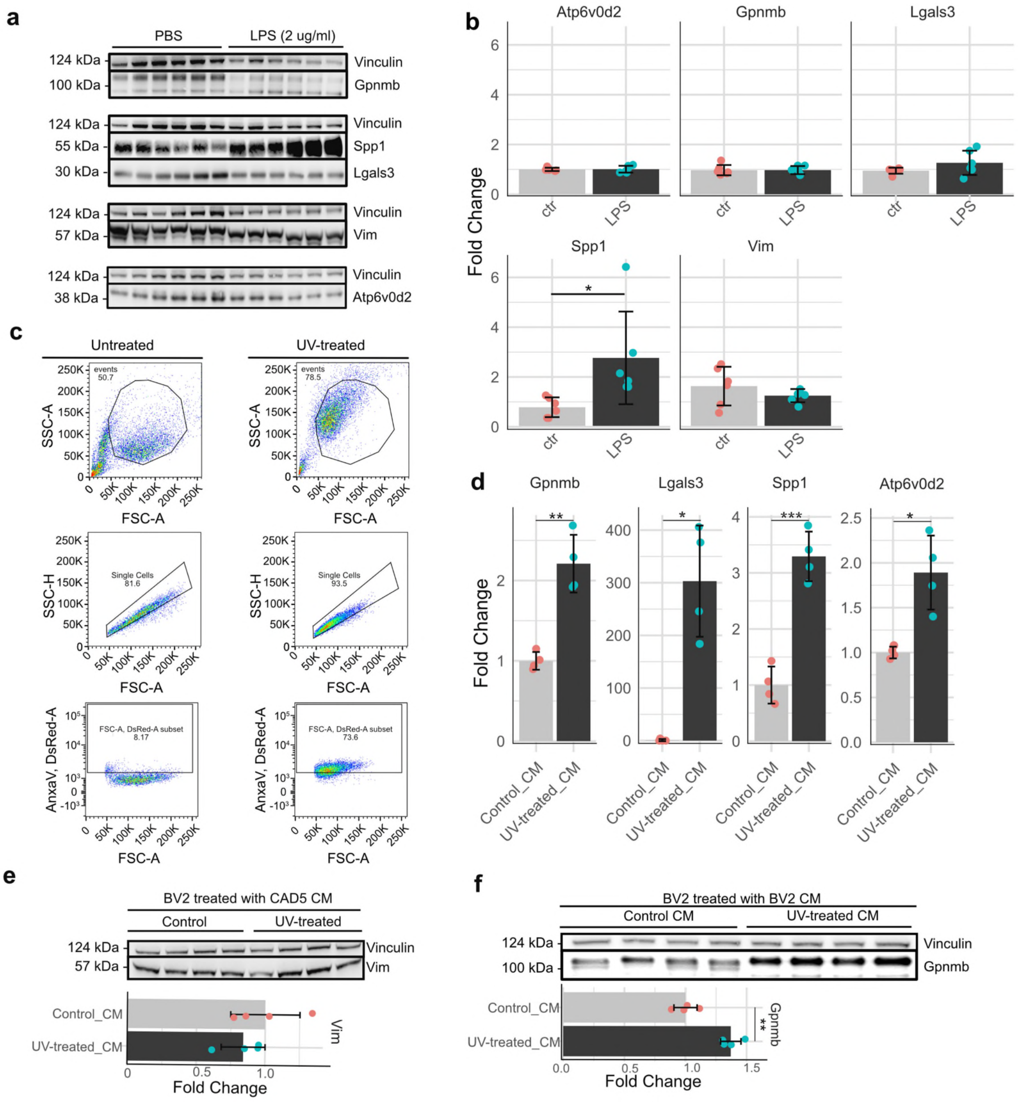

Supplementary Figure 13. a) Western blot of BV2 cells treated with PBS or LPS (2 μg/ml) for 24 hours, probing Gpnmb, Spp1, Lgals3, Vim, Atp6v0d2 and Vinculin. **b)** Densitometric analysis of panel E, showing fold change in protein expression (PBS vs. LPS). **c)** FACS analysis of Annexin-V (AnxaV) in UV-treated vs. untreated CAD5 cells confirms apoptosis. **d)** Densitometric analysis of Western blot (Figure 6A) in BV2 cells cultured with CM from UV-treated and untreated CAD5 cells. **e)** Western blot of BV2 cells treated with CM from UV-treated CAD5 cells, probing Vim vs. Vinculin with related densitometric analysis. **f)** Western blot of BV2 cells treated with CM from UV-treated BV2 cells, probing Gpnmb vs. Vinculin with related densitometric analysis. Significant differences between groups are indicated by asterisks (* $p<0.05$, ** $p<0.01$, *** $p<0.001$).

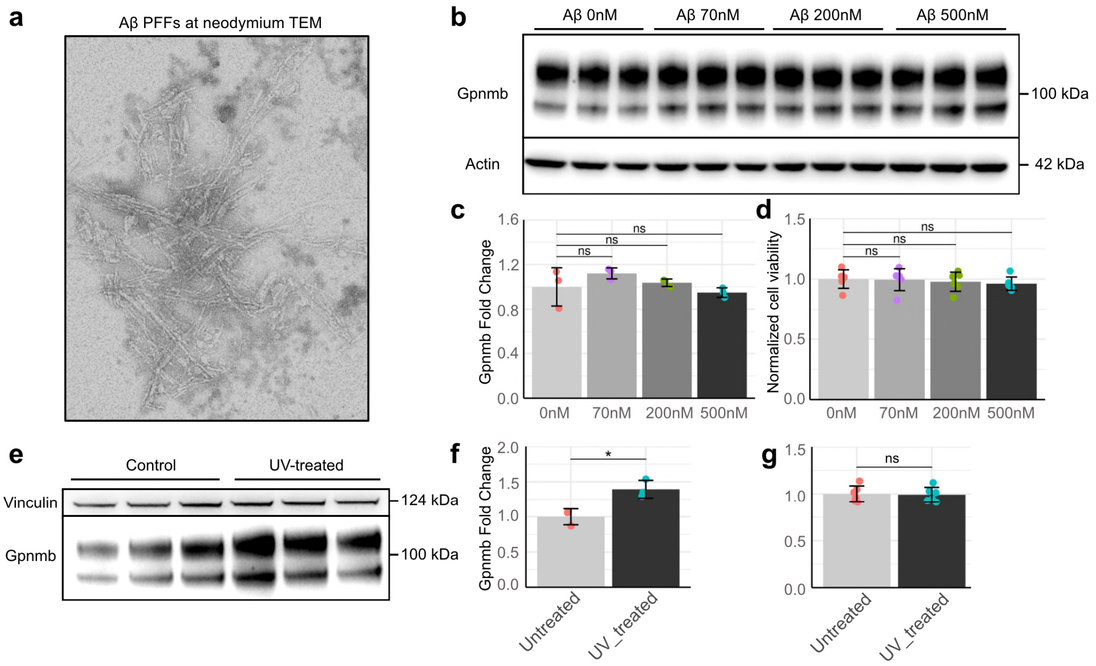

Supplementary Figure 16 a) Transmission electron microscopy (TEM) image of amyloid-β (Aβ) pre-formed fibrils (PFFs) generated under shaking conditions. **b)** Immunoblot analysis of Gpnmb expression in cells treated with increasing concentrations of Aβ fibrils (0–500 nM). Actin was used as a loading control. **c)** Densitometric quantification of Gpnmb protein levels normalized to Actin across Aβ concentrations. Data are shown as mean ± SEM. **d)** Cell viability assessed by CellTiter-Glo following treatment with increasing concentrations of Aβ fibrils, expressed relative to the 0 nM condition. **e)** Immunoblot of Gpnmb in BV2 cells cultured in control or UV-treated conditioned medium. Vinculin was used as a loading control. **f)** Quantification of Gpnmb protein levels normalized to Vinculin. **g)** Cell viability assessed by CellTiter-Glo in cells cultured in control or UV-treated conditioned medium, expressed relative to control. Significant differences between groups are indicated by asterisks (* $p<0.05$, ** $p<0.01$, *** $p<0.001$).

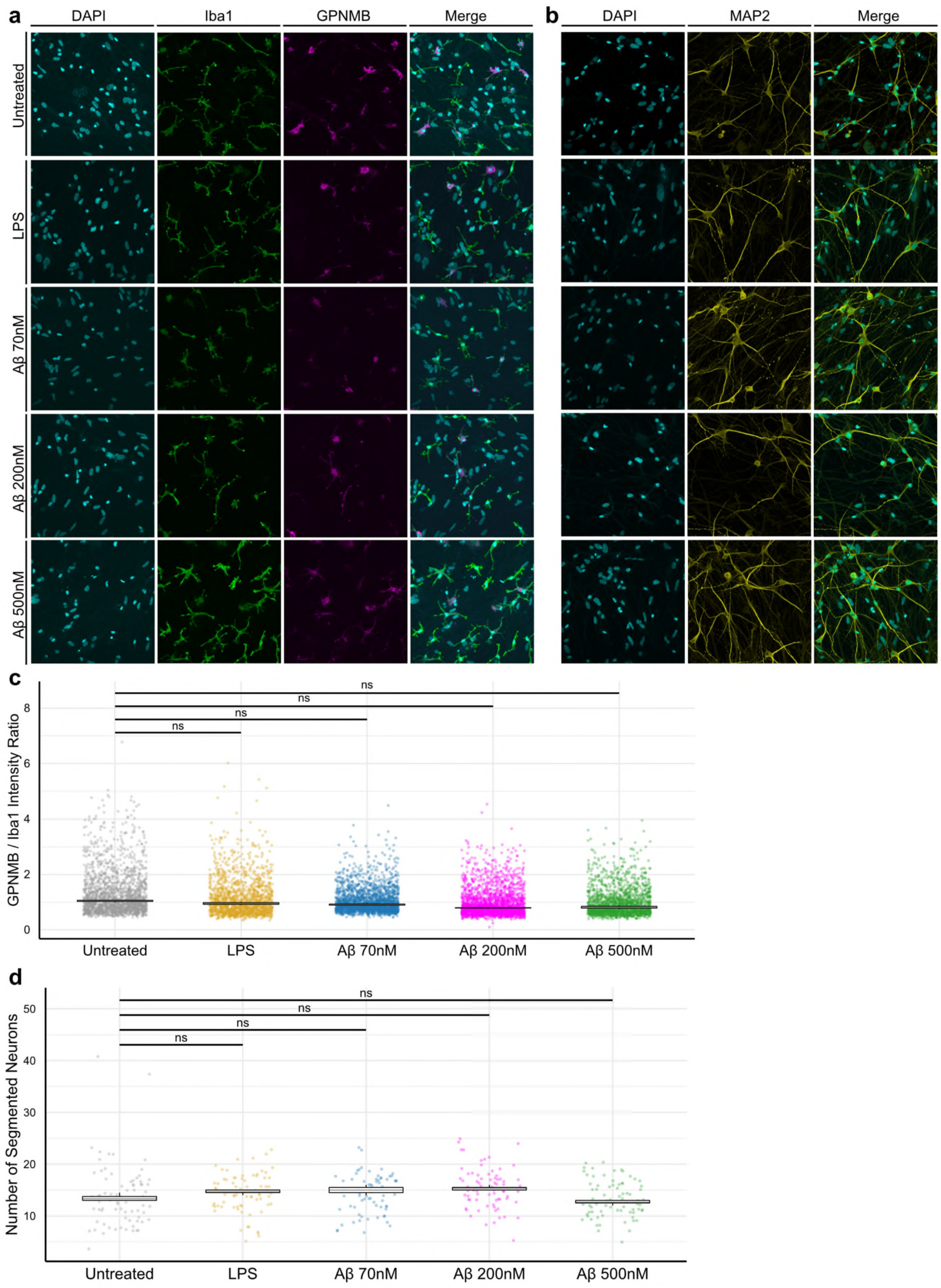

Supplementary Figure 17. **a**) Representative immunofluorescence images of cultured cells stained for DAPI (nuclei), Iba1 (microglia), and GPNMB under untreated conditions, LPS stimulation, or after treatment with amyloid-β (Aβ) at 70 nM, 200 nM, or 500 nM. Merged images show cellular localization. **b)** Immunofluorescence staining for DAPI and MAP2 to visualize neuronal density and morphology across the same treatment conditions, with merged images shown. The same wells shown in (a) were subsequently re-stained for MAP2 and re-imaged using the same regions of interest. Due to remounting of the plate on the microscope, minor positional shifts occurred between acquisitions; therefore, DAPI was re-imaged to document the imaging field. Neuronal MAP2 staining was not acquired during the initial imaging shown in (a), but all images derive from the same wells. **c)** Quantification of the GPNMB/Iba1 intensity ratio across untreated (from a panel), LPS-treated, and Aβ-treated cultures (70, 200, and 500 nM). Each dot represents an individual measurement, while boxplots indicate the distribution of replicate-level median values (n = 3 biological replicates per condition). Statistical analysis was performed using Wilcoxon rank-sum tests on replicate medians, comparing each condition against the untreated control, followed by Benjamini-Hochberg (BH) correction for multiple testing. **d)** Quantification of the number of segmented neurons per ROI across the same experimental conditions (from b panel). Individual dots represent single ROIs, and boxplots summarize replicate-level median values (n = 3 biological replicates per condition). Statistical comparisons were carried out as in (c), using replicate-level Wilcoxon tests versus untreated controls with BH correction.

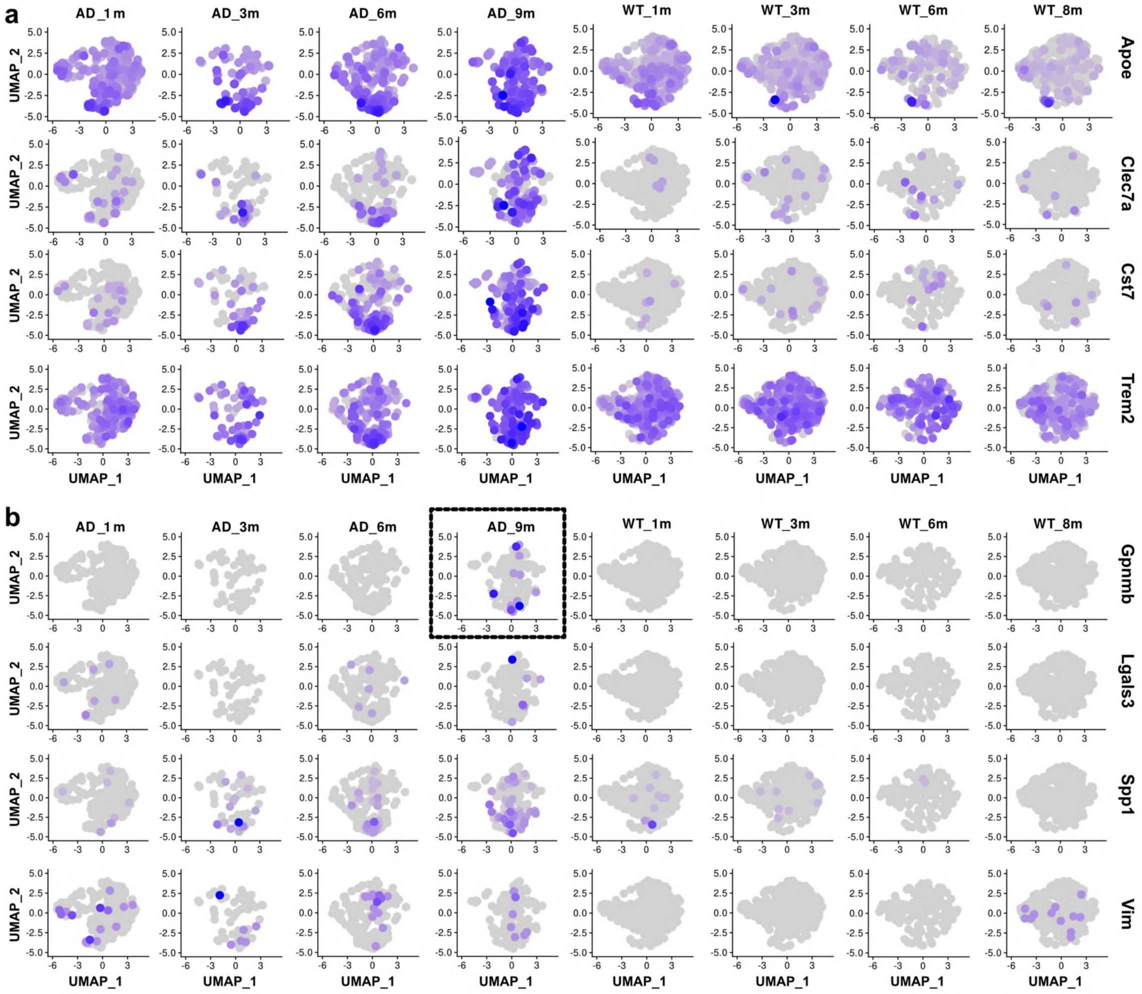

Supplementary Figure 18. UMAP visualization of a re-analysed single-cell RNA-sequencing dataset from Keren-Shaul et al., Cell (2017), profiling microglia from Alzheimer's disease (AD) model mice (5xFAD) and age-matched wild-type (WT) controls across multiple ages, showing **a)** Microglial UMAPs colored by expression of canonical DAM and microglial markers (*Apoe*, *Clec7a*, *Cst7*, and *Trem2*) across disease and control conditions, illustrating the progressive activation of DAM-like transcriptional programs in AD but not WT microglia, and **b)** UMAPs showing expression of *Gpnmb*, *Lgals3*, *Spp1*, and *Vim* genes, with *Gpnmb* expression restricted to a small subset of microglia at late AD stages (boxed).

References

1. Liu M, Zhu J, Zheng J, Han X, Jiang L, Tong X, et al. GPNMB and ATP6V1A interact to mediate microglia phagocytosis of multiple types of pathological particles. *Cell Reports.* 2025;44(3).
2. Androvic P, Schifferer M, Perez Anderson K, Cantuti-Castelvetri L, Jiang H, Ji H, et al. Spatial Transcriptomics-correlated Electron Microscopy maps transcriptional and ultrastructural responses to brain injury. *Nat Commun.* 2023;14(1):4115.
3. van der Vliet D, Di X, Shamorkina TM, Pavlovic A, van der Vliet IACM, Zeng Y, et al. Foamy microglia link oxylipins to disease progression in multiple sclerosis. *bioRxiv.* 2024:2024.10.18.619040.
4. Dolan MJ, Therrien M, Jereb S, Kamath T, Gazestani V, Atkeson T, et al. Exposure of iPSC-derived human microglia to brain substrates enables the generation and manipulation of diverse transcriptional states in vitro. *Nat Immunol.* 2023;24(8):1382-90.
5. Keren-Shaul H, Spinrad A, Weiner A, Matcovitch-Natan O, Dvir-Szternfeld R, Ulland TK, et al. A Unique Microglia Type Associated with Restricting Development of Alzheimer's Disease. *Cell.* 2017;169(7):1276-90.e17.
6. Li Q, Cheng Z, Zhou L, Darmanis S, Neff NF, Okamoto J, et al. Developmental Heterogeneity of Microglia and Brain Myeloid Cells Revealed by Deep Single-Cell RNA Sequencing. *Neuron.* 2019;101(2):207-23.e10.
7. Zhu K, Min J-H, Joshua V, Liu Y, Pieber M, Suerth V, et al. Disrupting microglial TGF-β signaling triggers region-specific pathology in the spinal cord. *bioRxiv.* 2023:2023.04.24.538074.
8. Slota JA, Sajesh BV, Frost KF, Medina SJ, and Booth SA. Dysregulation of neuroprotective astrocytes, a spectrum of microglial activation states, and altered hippocampal neurogenesis are revealed by single-cell RNA sequencing in prion disease. *Acta Neuropathologica Communications.* 2022;10(1):161.
9. !!! INVALID CITATION !!! (12, 13).
10. Liu Y, Guo J, Matoga M, Korotkova M, Jakobsson P-J, and Aguzzi A. NG2 glia protect against prion neurotoxicity by inhibiting microglia-to-neuron prostaglandin E2 signaling. *Nature Neuroscience.* 2024;27(8):1534-44.
11. Zhang J, Velmeshev D, Hashimoto K, Huang Y-H, Hofmann JW, Shi X, et al. Neurotoxic microglia promote TDP-43 proteinopathy in progranulin deficiency. *Nature.* 2020;588(7838):459-65.
12. Carroll JA, Race B, Williams K, Striebel J, and Chesebro B. Microglia Are Critical in Host Defense against Prion Disease. *J Virol.* 2018;92(15).
13. Damisah EC, Hill RA, Rai A, Chen F, Rothlin CV, Ghosh S, et al. Astrocytes and microglia play orchestrated roles and respect phagocytic territories during neuronal corpse removal in vivo. *Sci Adv.* 2020;6(26):eaba3239.
14. Bradford BM, McGuire LI, Hume DA, Pridans C, and Mabbott NA. Microglia deficiency accelerates prion disease but does not enhance prion accumulation in the brain. *Glia.* 2022;70(11):2169-87.
15. El Amki M, Lerouet D, Coqueran B, Curis E, Orset C, Vivien D, et al. Experimental modeling of recombinant tissue plasminogen activator effects after ischemic stroke. *Experimental Neurology.* 2012;238(2):138-44.
16. Han B, Zhou S, Zhang Y, Chen S, Xi W, Liu C, et al. Integrating spatial and single-cell transcriptomics to characterize the molecular and cellular architecture of the ischemic mouse brain. *Science Translational Medicine.* 2024;16(733):eadg1323.
17. Erdenebat T, Komatsu Y, Uwamori N, Tanaka M, Hoshika T, Yamasaki T, et al. Excitatory neuron-prone prion propagation and excitatory neuronal loss in prion-infected mice. *Front Mol Neurosci.* 2024;17:1498142.
